# Location-specific psychosocial and environmental correlates of physical activity and sedentary time in young adolescents: preliminary evidence for location-specific approaches from a cross-sectional observational study

**DOI:** 10.1186/s12966-022-01336-7

**Published:** 2022-08-26

**Authors:** Adrian Ortega, Carolina M. Bejarano, Christopher C. Cushing, Vincent S. Staggs, Amy E. Papa, Chelsea Steel, Robin P. Shook, Terry L. Conway, Brian E. Saelens, Karen Glanz, Kelli L. Cain, Lawrence D. Frank, Jacqueline Kerr, Jasper Schipperijn, James F. Sallis, Jordan A. Carlson

**Affiliations:** 1 Clinical Child Psychology Program, University of Kansas, 2005 Dole Human Development Center, 1000 Sunnyside Ave, Lawrence, Kansas, USA; 2 Center for Children’s Healthy Lifestyles and Nutrition, Children’s Mercy, Kansas, MO USA; 3 Cincinnati Children’s Hospital Medical Center, Division of Behavioral Medicine & Clinical Psychology, Cincinnati, USA; 4 Schiefelbusch Institute for Lifespan Studies, University of Kansas, Lawrence, Kansas, USA; 5 Biostatistics & Epidemiology, Health Services & Outcomes Research, Children’s Mercy, Kansas, MO USA; 6 School of Medicine, University of Missouri-Kansas City, Kansas City, MO USA; 7 Herbert Wertheim School of Public Health and Human Longevity Science, University of California, La Jolla, San Diego, California USA; 8 Department of Pediatrics, University of Washington & Seattle Children’s Research Institute, Seattle, Washington USA; 9 Perelman School of Medicine and School of Nursing, University of Pennsylvania, Philadelphia, PA USA; 10 School of Community and Regional Planning, University of British Columbia, Vancouver, British Columbia Canada; 11 Department of Sports Science and Clinical Biomechanics, University of Southern Denmark, Odense, Denmark; 12 Mary MacKillop Institute for Health Research, Australian Catholic University, Melbourne, Australia

**Keywords:** Obesity, Global positioning systems, Built environment, Psychosocial, Multilevel

## Abstract

**Background:**

A better understanding of the extent to which psychosocial and environmental correlates of physical activity are specific to locations would inform intervention optimization.

**Purpose:**

To investigate cross-sectional associations of location-general and location-specific variables with physical activity and sedentary time in three common locations adolescents spend time.

**Methods:**

Adolescents (*N* = 472,M_age_ = 14.1,SD = 1.5) wore an accelerometer and global positioning systems (GPS) tracker and self-reported on psychosocial (e.g., self-efficacy) and environmental (e.g., equipment) factors relevant to physical activity and sedentary time. We categorized each survey item based on whether it was specific to a location to generate psychosocial and environmental indices that were location-general or specific to either school, non-school, or home location. Physical activity (MVPA) and sedentary time were based on time/location match to home, school, or all “other” locations. Mixed-effects models investigated the relation of each index with location-specific activity.

**Results:**

The location-general and non-school physical activity psychosocial indices were related to greater MVPA at school and “other” locations. The school physical activity environment index was related to greater MVPA and less sedentary time at school. The home activity environment index was related to greater MVPA at home. The non-school sedentary psychosocial index was related to less sedentary time at home. Interactions among indices revealed adolescents with low support on one index benefited (i.e., exhibited more optimal behavior) from high support on another index (e.g., higher scores on the location-general PA psychosocial index moderated lower scores on the home PA environment index). Concurrent high support on two indices did not provide additional benefit.

**Conclusions:**

No psychosocial or environment indices, including location-general indices, were related to activity in all locations. Most of the location-specific indices were associated with activity in the matching location(s). These findings provide preliminary evidence that psychosocial and environmental correlates of activity are location specific. Future studies should further develop location-specific measures and evaluate these constructs and whether interventions may be optimized by targeting location-specific psychosocial and environmental variables across multiple locations.

**Supplementary Information:**

The online version contains supplementary material available at 10.1186/s12966-022-01336-7.

## Introduction

Adolescents engage in suboptimal levels of physical activity, with fewer than 25% of US teenagers meeting the national guidelines of 60 minutes of moderate-to-vigorous physical activity (MVPA) each day [1]. These low prevalence rates pose a significant public health threat given that low physical activity during adolescence confers physical and psychosocial health risks, can track into adulthood, and are related to future health issues such as cardiovascular disease, diabetes, cancer, and obesity [2–5]. For sedentary time, although findings have been somewhat mixed when using device-based measures, some evidence suggests that high sedentary time is associated with poor health in youth even when accounting for differences in MVPA [6]. Although physical activity and sedentary time are interrelated, research supports using separate intervention strategies that purposely target each of these behaviors [7, 8].

Correlates and determinants of adolescent physical activity and sedentary time span multiple levels of influence. Ecological models of health behavior emphasize the role of individual-level variables, environmental variables, and interactions among multilevel variables, in shaping physical activity [9–11]. For example, extant research indicates individual-level variables such as physical activity self-efficacy, social support, decisional balance, and enjoyment are related to overall physical activity in youth [12–14]. Research on environmental correlates has documented benefits of access to outdoor locations that include walkable areas, greenspaces, and safe environments for supporting higher physical activity [15–19]. Accordingly, many physical activity-related interventions in youth target individual (e.g., self-efficacy, motivation) and/or environment variables (e.g., classroom environment) [20–22].

Interventions for improving physical activity in youth generally produce small changes in objectively-assessed MVPA [21, 23]. One possible reason is because interventions do not commonly provide strategies for improving or sustaining physical activity across multiple settings/locations. One hypothesis is that interventions with location-general approaches (i.e., strategies not adapted to or not specific to a certain location), or approaches linked to only one or two locations, may have limited success across multiple locations due to location-specific barriers. For example, location-specific strategies to increase environmental supports for school-based physical activity likely do not impact home-based activity, and location-general strategies for increasing self-efficacy for reducing sedentary time may be overridden by a school environment that is highly supportive of sedentary time. However, prior to designing interventions that improve environments across various locations or seek to augment psychosocial factors across different locations, it is important to understand whether location-general and location-specific factors differ in their associations with location-specific activity. Few studies [24] have examined location-general and location-specific influences on physical activity and sedentary time within different locations. Therefore, more research on the associations of location-general and location-specific psychosocial attributes and environmental features in relation to adolescents’ physical activity and sedentary behavior could inform more targeted and tailored interventions for sustaining physical activity as well as interventions for reducing sedentary behavior across locations that play a large role in adolescents’ lives, such as their homes and schools.

Most studies that differentiated between location-general and location-specific influences primarily focused on environmental variables, such as assessing home neighborhood features and examining their association with location-specific (e.g. physical activity that occurs in the neighborhood) and overall physical activity accumulation [25]. Only one study could be found that investigated location-specific psychosocial correlates of location-specific physical activity [24]. Ommundsen and co-authors [24] found that location-specific psychosocial variables such as teacher support were strongly related to youth-reported school physical activity, while parental support for youth physical activity was associated with youth-reported leisure-time physical activity (i.e., outside of the school setting) but not school-located physical activity. Physical activity enjoyment, peer social support, and perceived competence were assessed in general, not specific to a location, and these non-location-specific variables were related to both youth-reported school and leisure-time physical activity [24]. Relatedly, Cushing and co-investigators [26] and Dunton [27] discussed how psychosocial attributes can demonstrate dynamic, time-varying properties within individuals, suggesting that youth may be differentially motivated to engage in exercise across different contexts (e.g., feeling efficacious about physical activity at home, but not at school). Limitations of the aforementioned studies included capturing a small range of location-specific constructs and relying on self-reported rather than device-based measures of physical activity across locations (e.g., Global Positioning System (GPS)).

There is also a gap in the understanding of how psychosocial and environmental variables interact in relation to adolescents’ activity [28]. Previous research has primarily been limited to the investigation of interactions between psychosocial and home neighborhood environment variables, [29–34] with less attention to interactions within other environments (e.g., school) and interactions between location-general and location-specific variables. Such information would additionally support a more holistic model of the complexity of variables influencing adolescents’ activity patterns.

The purposes of the present analyses (using historical data) were to investigate the associations of both location-general (i.e., across locations or not specific to any location such as one’s overall self-efficacy) and location-specific psychosocial and environmental variables (i.e., measures that are specific to a location such as exercise equipment at home) with adolescents’ physical activity and sedentary time at home, school, and all “other” locations. In other words, we were interested in comparing how measures that are general versus measures that are specific to a location relate to physical activity and sedentary time across and within locations. It was hypothesized that location-specific psychosocial and environmental variables would be consistently associated with location-specific activity in the matching location and not in the mismatching location (e.g., location-specific school factors would be associated with activity at school but not with activity at home). To evaluate this hypothesis, we determined the frequency of matches and mismatches among the observed location-specific psychosocial/environmental and activity associations. Location-general psychosocial variables were not expected to generalize across all locations, so, it was hypothesized that the location-general variables would not be significantly associated with location-specific activity across *all* locations. To better understand the interplay between multilevel variables, this study also explored interactions between levels of influence (i.e., psychosocial and environmental), location-general and location-specific variables, and location-specific variables linked to different locations (e.g., non-school physical activity psychosocial variables and school physical activity environment variables). That is to say that we explored how these general versus location-specific measures moderate the impact of each other on physical activity and sedentary time. Taken together, the findings were expected to inform ecological models of health behavior [9] as well as whether, where, and which approaches might be needed for testing more optimized multilevel and multi-location interventions. This exploratory study fills gaps in previous literature on location-specific psychosocial and environmental correlates of physical activity by utilizing device-based measures of physical activity and location and capturing a broader range of explanatory constructs.

## Methods

### Participants and procedures

Present analyses involved historical data collected from the cross-sectional, observational Teen Environment and Neighborhood (TEAN) Study [35]. Participants 12–16 years of age and one of their parents were recruited from 447 census block groups spanning the Baltimore, MD-Washington, DC and Seattle-King County, WA metropolitan areas from 2009 to 2011. Recruitment was balanced by season and evenly stratified across four quadrants representing combinations of neighborhoods (defined as census block groups) that were high or low neighborhood walkability and high or low median household income [35]. Data collection took place during the school year only.

Potential participants were identified through a purchased list from a marketing company and were contacted by phone to gauge their interest in the study and complete eligibility screening. Adolescents were excluded from the study if they had any physical, medical, or cognitive limitations that would affect their physical activity or impact their ability to complete measures. Eligible and interested adolescents were instructed to wear an accelerometer and GPS tracker for seven days during waking hours. A total of 928 adolescents participated. Present analyses excluded adolescents who did not receive a GPS tracker or record any GPS data (*n* = 130), whose home address was not available in the geocoding database (*n* = 29; e.g., P.O. Box or otherwise failed to geocode), or who did not provide their school’s name/address or were homeschooled (*n* = 93). Adolescents who did not wear both devices for ≥1 valid school day and ≥ 1 valid weekend day (*n* = 204) were also excluded to improve the likelihood that data were representative of a typical week of activity. Valid days were defined as those with ≥8 hours of concurrent data from both devices. Valid school days were operationalized as weekdays during which the participant spent ≥200 minutes at their school as measured by the GPS. The present analyses included 472 adolescents.

### Measures

#### Demographics and anthropometrics

Adolescent-parent dyads self-reported demographic information including respective age, sex, and race/ethnicity (dichotomized as white non-Hispanic versus non-white or Hispanic). The parent reported the highest education attained by any adult in the household (dichotomized as college degree versus less education); parental marital status (married/living with partner versus other); and the approximate annual household income. Adolescents self-reported their height and weight using provided instructions or, when available, reported anthropometric measurements taken at a clinic or school within the previous month.

#### GPS and location assignment

Adolescents wore a GlobalSat DG-100 GPS tracker (GlobalSat, New Taipei City, Taiwan), with latitude and longitude collected every 30 seconds. Adolescents were instructed how to wear the device and given directions to charge the device each night. Adolescents’ home and school addresses were geocoded in ArcGIS (ESRI, Inc., Redlands, CA). Consistent with previous studies [15, 36], we classified each adolescents’ home and school locations by creating a 50-m circular buffer around the point resulting from geocoding the home address and a 15-m buffer around the geocoded school parcel respectively. Each participant’s GPS points were overlayed with their location polygons (e.g., home buffer) to determine time and activity within each location. These spatial analyses were performed in PostgreSQL (PostgreSQL Global Development Group, Berkeley, CA) to categorize each GPS point by the following locations: at home (within the home buffer), at school (within the school buffer), or all “other” locations (i.e., any location other than the home and school buffers). Transport/trips outside the home and school locations were classified as part of the “other” location.

#### Physical activity and sedentary time

Adolescents wore ActiGraph accelerometers (models: 7164, 87.8%; GT1M, 8.0%; GT3X, 3.4%) on a belt, with the accelerometer positioned on their hip. We defined non-wear periods as 30+ minute bouts of consecutive epochs with 0 accelerometer counts and subsequently excluded these periods from analyses. The Evenson cut points [37] were applied to activity counts on the vertical axis within each 30-second epoch to classify MVPA, and the common cut point of ≤100 counts per minute was used to classify sedentary time.

#### Integration of GPS and accelerometer data

The integration and processing of the GPS and accelerometer data has been previously described [36]. In brief, the accelerometer and GPS devices were synchronized by time during initialization. Epochs with periods of missing GPS data or accelerometer non-wear time were removed from the dataset during data processing. The remaining GPS and accelerometer data were linked by nearest time stamp using the Personal Activity and Location Measurement System (PALMS) Version 4 (Center for Wireless and Population Health Systems, La Jolla, CA) and then processed to create overall and location-specific MVPA and sedentary time variables for each participant. The PALMS systems also performed some filtering of invalid GPS fixes caused by satellite interference as described PALMS User Guide in Additional file 1. For the school location, variables were derived for school days only (e.g., average minutes/day of MVPA across school days). For the home and “other” locations, variables were derived for a “weighted week”, calculated as ([mean daily values across school days*5] + [mean daily values across non-school days*2]) ÷ 7, to generate an average minutes/day of MVPA and sedentary time in these locations, similar to previous protocols [15]. If participants did not spend ≥30 minutes/day in a location on average across days, the activity variables for those locations were set to missing. This location-specific time requirement aimed to increase the likelihood the data were representative of the adolescent’s typical activity in the location.

#### Psychosocial and environmental variables

Adolescents self-reported on psychosocial and environment variables using previously validated scales about physical activity and sedentary behavior that were based on Social Cognitive Theory as well as the Transtheoretical Model of Health Behavior [38]. The psychosocial constructs included self-efficacy for, social support for, decisional balance of, and enjoyment of physical activity and sedentary behavior (although some sedentary scales referred to reducing sedentary behaviors). These scales were developed for adolescents and have been evaluated among adolescents for test-retest reliability and construct validity [39]. Environment scales included measures of the perceived school physical activity environment [40], personal electronics (sedentary environment) [41], and the home physical activity environment, which have all been previously evaluated with adolescents [41–44]. Most items were Likert scales with response formats of agreement/disagreement or frequency (see Supplementary Table 1 in Additional file 2), although some items were checklists (e.g., endorsing yes/no on different types of screens in the bedroom) and two items captured minutes of physical activity opportunities in school.

**Fig. 1:**
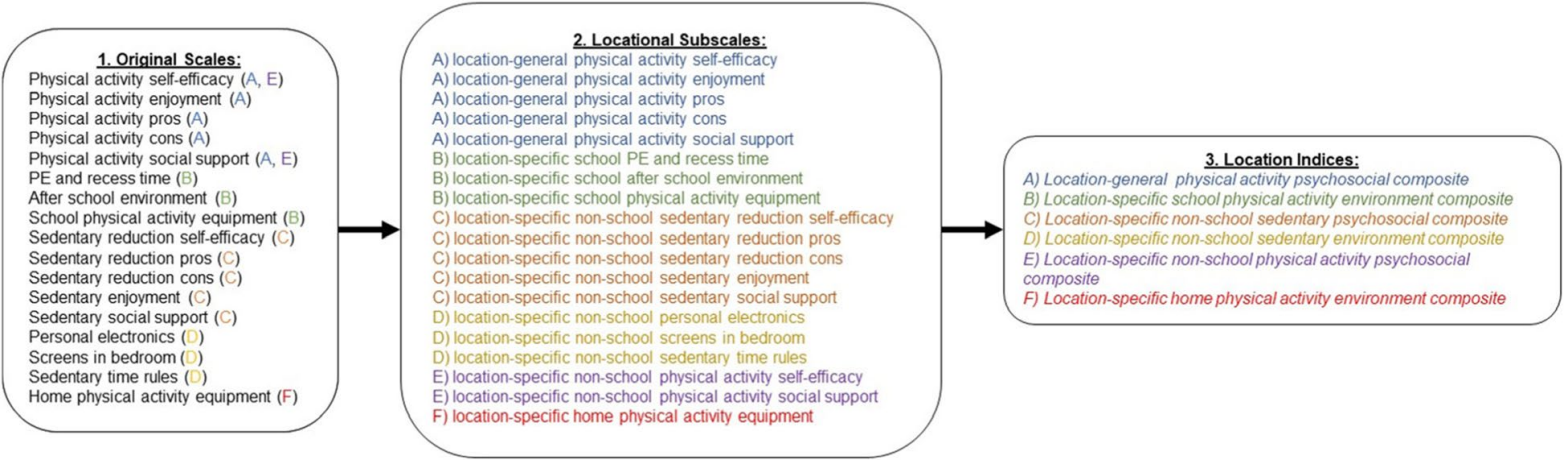
Process of creating locational subscales and indices from original scales. Note. Items within the 17 previously-validated (original) scales shown in Box 1 were examined for their location specificity and assigned accordingly to the most relevant location-general or location-specific subscale in Box 2, which were created specifically for the present analyses. Subscales were then combined by location within activity type (physical activity or sedentary) to form the final indices in Box 3. The letters in the parenthesis following the original scales in Box 1 indicate the subscales and indices to which items from the original scale were assigned. Appendix Table 6 shows the item content for all items from the original scales in Box 1 as well as each item’s assigned subscale

The original scales were not already grouped by location (e.g., home, school), so we undertook a process of creating the locational-specific subscales and indices for the present analyses, as illustrated in Fig. 1. We first grouped the items within each of the original scales a priori into subscales categorized by the locational specificity of the item content based on consensus across members of the research team. We used the content of the item to determine the most relevant location(s) to which the item related. For example, the item *“How sure are you that you can get up early, even on weekends, to do physical activity?”,* which was from the original physical activity self-efficacy scale, was categorized in the non-school physical activity self-efficacy subscale because the item queries about activity that would occur outside of school hours. We summed the item scores within each subscale to calculate the subscale scores. All items within each subscale used the same response format/scale, so each item was equally weighted in the subscale score. This process resulted in 5 location-general subscales (items that were not specific to any location), 3 location-specific school subscales (items specific to school), 10 location-specific non-school subscales (items that could be linked to the home or at least one other specific location that was not school), and 1 location-specific home subscale (items specific to the home location). Of these 19 subscales, 12 assessed psychosocial factors and 7 assessed environmental factors.

These subscales were then combined by transforming the values to z-scores and averaging the z-scores to create six final indices representing location-general and location-specific psychosocial and environmental features for physical activity and sedentary time. The z-score approach was used so that each subscale would be equally weighted in the index scores. Analyzing composite indices was expected to produce more robust associations with outcomes than single items or subscales. The six emerging indices included a (A) *general physical activity psychosocial index,* reflecting individual attributes for increasing physical activity not specific to any location; (B) *school physical activity environment index,* representing the quality of adolescents’ perceived school environment for supporting physical activity at school*;* (C) *non-school sedentary psychosocial index,* capturing individual attributes for reducing sedentary time in locations outside school, (D) *non-school sedentary environment index,* indicating the quality of adolescents’ environment outside of school for reducing sedentary behavior; (E) *non-school physical activity psychosocial index,* assessing adolescents’ psychosocial variables for physical activity particular to home and other locations; and (F) *home physical activity environment index,* reflecting home equipment for physical activity. More information on the items and scales comprising each index is included in Supplementary Table 1 (see Additional file 2). Descriptive statistics for psychosocial and environmental subscales are displayed in Supplementary Table 2 in Additional file 3. The allocation of items to subscales and subscales to indices was mutually exclusive, with no item belonging to more than one subscale and no subscale belonging to more than one index. Prior to creating subscales, items were reverse scored as needed to reflect positive valences toward promoting more physical activity and less sedentary time.

### Data analytic plan

Sample characteristics were summarized using descriptive statistics. All models were mixed-effects linear regression models, fitted with the “MIXED” command in SPSS version 24 (IBM SPSS Statistics, IBM Corporation). A random intercept was included to account for the nesting of participants within census block groups, as the participant was the unit of analysis. Location-specific (school, home, “other” location) MVPA and sedentary time were investigated as dependent variables in separate models, regressed first on individual-level sociodemographic variables (age, sex, race/ethnicity, and parent education entered into the same model) and then on each of the 6 psychosocial and environmental indices in separate models. Another set of models regressed each overall and location-specific MVPA and sedentary variable on each of the 19 psychosocial and environmental subscales (one model per each dependent variable – subscale combination), which was provided for supplemental information. Although the study hypotheses revolved around location-specific MVPA and sedentary time, overall MVPA and sedentary time (across locations) were investigated as additional dependent variables using the aforementioned modelling approach to contextualize the location-specific findings. A final set of models investigated interactions between the psychosocial and environmental indices, location-general and location-specific indices, and location-specific indices in different locations in explaining adolescents’ MVPA and sedentary time.

All models were adjusted for study design variables (neighborhood walkability and income categories); the adolescent’s age, sex, and race/ethnicity; parent education; ActiGraph model; number of school and non-school days of device wear; and average minutes/day of wear time in the respective location. All dependent and independent variables were converted to z scores to have a mean of zero (i.e., mean center) and standard deviation of one to derive standardized regression coefficients, facilitate comparison of effect sizes across variables and models, and create orthogonalized interaction terms. Benchmarks for interpreting the magnitude of the standardized regression coefficients were small (β = .10), small-to-moderate (β = .20), and moderate (β = .30) [45].

We labeled significant associations (*P* < .05) between a location-specific index and the corresponding location-specific activity variable (e.g., school environment with school MVPA) as “matches” and significant associations between a location-specific index and a non-corresponding location activity variable as “mismatches”. We did not label any associations involving overall activity variables because we did not have hypotheses around overall activity. In the results section, we counted the frequency of matches and mismatches for the significant location-specific associations to evaluate our primary hypothesis. For interactions, we probed those with *P*-values ≤0.10 to determine the pattern and direction of association for each independent variable of interest at different levels of the other independent variable of interest. This more liberal *p*-value was selected for probing interactions because power to detect interactions is lower than for detecting main effects [46], and we sought to minimize risk for Type II error when investigating group differences. Plots were created by calculating the value of the dependent variable based on the regression equation using values for the continuous independent variables comprising the interaction that reflected 1 SD above and below mean, with all continuous and dichotomous covariates mean centered. Original metrics (e.g., minutes/day) of the activity variables were used when probing interactions. We centered the Y axis of these plots at the mean value for the dependent variable and adjusted the axis bounds to reflect + 1 SD and − 1 SD below the mean.

## Results

### Sample characteristics and descriptive statistics

On average, adolescents were 14.1 years old (*SD* = 1.5). See Table 1 for a more detailed description of demographic characteristics of the current sample. Participants simultaneously wore the accelerometer/GPS for 5.19 (*SD* = 1.30) days on average. Over 96% of participants had ≥3 wear days. Participants’ mean MVPA was 22.9 (*SD* = 15.0), 6.6 (*SD* = 7.1), 15.9 (*SD* = 16.1), and 39.5 (*SD* = 21.5) minutes/day at school, at home, in “other” locations, and overall, respectively. Participants’ mean sedentary time was 305.2 (*SD* = 85.2), 135.2 (*SD* = 98.6), 135.7 (*SD* = 96.4), and 483.6 (*SD* = 80.5) minutes/day at school, at home, in “other” locations, and overall, respectively. MVPA and sedentary time were moderately and negatively correlated within each location and overall (e.g., increases in home MVPA correlated with decrease in home sedentary time; Supplementary Table 3 in Additional file 4). Adolescent sex, parental marital status, parental education, neighborhood walkability, and family income were mostly comparable between this analysis sample and the full TEAN sample (*N* = 928). However, this study’s sample comprised a significantly greater proportion of White non-Hispanic youth compared the full TEAN sample (71.3% vs. 66.3%, *P* = .006). Demographics for the full TEAN sample can be found in previous papers [35].

**Table 1:** Demographic Characteristics

	*N*	Mean (SD) or %
Age	472	14.12 (1.47)
Sex		
Male	233	49.4
Female	239	50.6
Race/Ethnicity		
Non-white or Hispanic	135	28.7
White, non-Hispanic	335	71.3
Parent’s Marital Status		
Married or living with partner	391	83.0
Not married or living with partner	80	17.0
Parental Education		
Completion of college degree or higher	351	74.7
Other	119	25.3
Neighborhood Walkability		
Low walkability	257	54.4
High walkability	215	45.6
Approximate Annual Household Income		
< $50,000	67	14.7
$50,000 - < $100,000	176	38.7
≥ $100,000	212	46.6

### Subscale model results

The models involving the indices are presented below and in Table 2 as the main findings. The results for the subscales were generally consistent with the results for the indices; we present these in Table 3 for comprehensiveness and to show the drivers of the associations between the indices and activity variables. Table 3 also presents associations of sociodemographic characteristics with activity variables. These sociodemographic models were not adjusted for the locational indices/subscales to show the general associations between these correlates and the locational outcomes.

**Table 2:** Relations of location-specific and location-general psychosocial and environmental indices with adolescents’ location-specific and overall physical activity and sedentary time^a^

	Β^*b*^ (SE)							
	MVPA				Sedentary time			
	School	Home	Other	Overall	School	Home	Other	Overall
**Location-general variables**								
General physical activity psychosocial index	0.15** (0.04)	0.03 (0.03)	0.11** (0.04)	0.18** (0.04)	−0.04 (0.03)	− 0.01 (0.01)	− 0.04 (0.02)	− 0.07* (0.04)
**Location-specific school variables**								
School physical activity environment index	0.10* (0.04)	0.04 (0.04)	−0.01 (0.04)	0.06 (0.04)	−0.06* (0.03)	0.01 (0.01)	0.00 (0.02)	−0.05 (0.04)
**Location-specific non-school variables**								
Non-school sedentary psychosocial index	0.07 (0.04)	0.07 (0.04)	0.04 (0.04)	0.10* (0.04)	−0.01 (0.03)	−0.03* (0.01)	− 0.02 (0.02)	−0.07 (0.04)
Non-school sedentary environment index	−0.02 (0.04)	0.00 (0.04)	−0.01 (0.04)	−0.03 (0.05)	0.01 (0.03)	−0.01 (0.01)	0.02 (0.02)	0.02 (0.04)
Non-school physical activity psychosocial index	0.13** × (0.04)	0.07 (0.04)	0.15** (0.04)	0.21** (0.04)	−0.04 (0.03)	−0.01 (0.01)	− 0.03 (0.02)	−0.09* (0.04)
Home physical activity environment index	0.07 (0.04)	0.12** (0.04)	0.10* × (0.04)	0.16** (0.04)	−0.01 (0.03)	−0.03* (0.01)	− 0.02 (0.02)	−0.07 (0.04)

^a^All models were adjusted for participant age, sex, race/ethnicity, parent education, study design factors, number of days of accelerometer wear, number of school days, and accelerometer wear time in each location. Each index was tested in a separate model;

^b^Values are standardized regression coefficients (β) and standard errors (SE), with both the independent and dependent variables standardized to have a mean of zero and standard deviation of 1. These values represent the standardized increases (positive coefficients) or decreases (negative coefficients) in the outcome variables per standard deviation increase on the index variable. Benchmarks for interpreting the magnitude of the coefficients were small (β = .10), small-to-moderate (β = .20), and moderate (β = .30);

Bolded cells with × symbol depict significant associations that were categorized as ‘mismatches’ between the location reflected in the index and activity variable, there were 2 mismatches in total;

We did not label associations involving overall activity variables because there were no hypotheses around overall activity- these results are presented for context;

*P < 0.05;

**P < 0.01

**Table 3:** Relations of location-specific and location-general subscales with adolescents’ location-specific and overall physical activity and sedentary time^a^

	β (SE)^b^							
	MVPA				Sedentary time			
	School	Home	Other	Overall	School	Home	Other	Overall
**Sociodemographic variables**^*c*^								
Age	−0.01 (0.03)	− 0.09** (0.03)	− 0.07** (0.03)	− 0.08** (.03)	0.13** (0.02)	0.05** (0.01)	0.04** (0.01)	0.21** (0.02)
Sex (Females = 1)	−0.56** (0.08)	− 0.31** (0.07)	− 0.36** (0.08)	−0.66** (0.08)	0.34** (0.05)	0.02 (0.02)	0.12** (0.03)	0.41** (0.07)
Race/ethnicity (White, non-Hispanic = 1)	− 0.04 (0.09)	0.05 (0.08)	0.04 (0.09)	0.00 (0.10)	0.09 (0.06)	0.00 (0.03)	0.04 (0.04)	0.12 (0.08)
Parent education (college degree or higher = 1)	0.15 (0.10)	0.24** (0.09)	−0.03 (0.09)	0.12 (0.10)	−0.03 (0.06)	− 0.06 (0.03)	0.02 (0.04)	−0.07 (0.09)
**Location-general variables**								
Physical activity self-efficacy	0.10* (0.05)	0.03 (0.04)	0.00 (0.05)	0.07 (0.05)	−0.02 (0.03)	0.01 (0.01)	0.01 (0.02)	0.01 (0.04)
Physical activity enjoyment	0.02 (0.05)	0.08 (0.04)	0.13** (0.04)	0.13* (0.05)	−0.03 (0.03)	−0.02 (0.01)	− 0.04* (0.02)	− 0.08 (0.04)
Physical activity pros	0.02 (0.04)	−0.02 (0.04)	− 0.03 (0.04)	− 0.02 (0.05)	0.03 (0.03)	0.01 (0.01)	0.00 (0.02)	0.04 (0.04)
Physical activity cons^d^	−0.01 (0.04)	0.04 (0.04)	0.02 (0.04)	0.02 (0.05)	0.01 (0.03)	0.03 (0.01)	0.01 (0.02)	0.05 (0.04)
Physical activity social support	0.06 (0.05)	−0.02 (0.04)	0.06 (0.04)	0.08 (0.05)	−0.03 (0.03)	0.01 (0.01)	−0.01 (0.02)	− 0.02 (0.04)
General physical activity psychosocial index	0.15** (0.04)	0.03 (0.03)	0.11** (0.04)	0.18** (0.04)	−0.04 (0.03)	−0.01 (0.01)	− 0.04 (0.02)	−0.07* (0.04)
**Location-specific school variables**								
PE and recess time	0.23** (0.04)	0.13** (0.04)	0.02 (0.04)	0.18** (0.05)	−0.13** (0.03)	0.04 (0.05)	0.00 (0.02)	−0.11** (0.04)
After school environment	0.03 (0.04)	0.02 (0.04)	0.04 (0.04)	0.06 (0.04)	−0.01 (0.03)	−0.03 (0.05)	− 0.01 (0.02)	−0.04 (0.03)
School physical activity equipment	−0.08 (0.05)	−0.07 (0.04)	− 0.07 (0.04)	−0.13** (0.05)	0.03 (0.03)	0.01 (0.04)	0.02 (0.02)	0.06 (0.04)
School physical activity environment index	0.10* (0.04)	0.04 (0.04)	−0.01 (0.04)	0.06 (0.04)	−0.06* (0.03)	0.01 (0.01)	0.00 (0.02)	−0.05 (0.04)
**Location-specific non-school variables**								
Sedentary reduction self-efficacy	0.08 (0.04)	0.01 (0.04)	0.01 (0.04)	0.07 (0.05)	−0.04 (0.03)	−0.02 (0.01)	0.01 (0.02)	−0.06 (0.04)
Sedentary reduction pros	−0.08 (0.04)	−0.06 (0.04)	− 0.02 (0.04)	−0.09 (0.04)	0.05 (0.03)	0.02 (0.01)	0.02 (0.02)	0.09* (0.04)
Sedentary reduction cons^d^	−0.04 (0.05)	0.00 (0.05)	0.01 (0.05)	−0.01 (0.05)	0.00 (0.03)	0.00 (0.01)	0.01 (0.02)	0.01 (0.04)
Sedentary enjoyment^d^	−0.08 (0.05)	−0.17** (0.04)	− 0.06 (0.05)	−0.14** (.05)	0.03 (0.03)	0.04** (0.01)	0.04* (0.02)	0.12** (0.04)
Sedentary social support^d^	0.03 (0.04)	0.04 (0.04)	−0.01 (0.04)	0.01 (0.04)	−0.02 (0.03)	0.00 (0.01)	0.01 (0.02)	−0.01 (0.04)
Non-school sedentary psychosocial index	0.07 (0.04)	0.07 (0.04)	0.04 (0.04)	0.10* (0.04)	−0.01 (0.03)	−0.03* (0.01)	− 0.02 (0.02)	−0.07 (0.04)
Personal electronics ^d^	−0.06 (0.04)	0.01 (0.04)	−0.05 (0.04)	−0.07 (0.05)	0.06* (0.03)	0.02 (0.01)	0.02 (0.02)	0.08* (0.04)
Screens in bedroom ^d^	0.07 (0.05)	0.00 (0.04)	0.05 (0.04)	0.09 (0.05)	−0.06* (0.03)	−0.01 (0.01)	− 0.03 (0.02)	−0.09* (0.04)
Sedentary time rules	−0.02 (0.04)	0.01 (0.04)	−0.02 (0.04)	−0.01 (0.05)	0.01 (0.03)	0.00 (0.01)	0.01 (0.02)	0.01 (0.04)
Non-school sedentary environment index	−0.02 (0.04)	0.00 (0.04)	−0.01 (0.04)	−0.03 (0.05)	0.01 (0.03)	−0.01 (0.01)	0.02 (0.02)	0.02 (0.04)
Physical activity self-efficacy	0.12** (0.04)	0.04 (0.04)	0.16** (0.04)	0.21** (0.04)	−0.03 (0.03)	−0.01 (0.01)	− 0.04* (0.02)	−0.08* (0.04)
Physical activity social support	0.03 (0.04)	0.04 (0.04)	0.01 (0.04)	0.04 (0.05)	−0.02 (0.03)	−0.01 (0.01)	0.00 (0.02)	−0.03 (0.04)
Non-school physical activity psychosocial index	0.13** (0.04)	0.07 (0.04)	0.15** (0.04)	0.21** (0.04)	−0.04 (0.03)	−0.01 (0.01)	− 0.03 (0.02)	−0.09* (0.04)
Home physical activity equipment	0.07 (0.04)	0.12** (0.04)	0.10* (0.04)	0.16** (0.04)	−0.01 (0.03)	−0.03* (0.01)	− 0.02 (0.02)	−0.07 (0.04)
Home physical activity environment index	0.07 (0.04)	0.12** (0.04)	0.10* (0.04)	0.16** (0.04)	−0.01 (0.03)	−0.03* (0.01)	− 0.02 (0.02)	−0.07 (0.04)

^a^All models were adjusted for participant age, sex, race/ethnicity, parent education, study design factors, number of days of accelerometer wear, number of school days, and accelerometer wear time in each location;

^b^Values are standardized regression coefficients (β) and standard errors (SE), with both the independent and dependent variables standardized to have a mean of zero and standard deviation of 1. These values represent the standardized increases (positive coefficients) or decreases (negative coefficients) in the outcome variables per standard deviation increase on the index variable. Benchmarks for interpreting the magnitude of the coefficients were small (β = .10), small-to-moderate (β = .20), and moderate (β = .30);

^*c*^Sociodemographic variables were analyzed first as a separate group of predictors without the index scales in the models to obtain these values and show general associations between sociodemographic correlates of physical activity and the outcome variables;

*P < 0.05;

**P < 0.01;

^d^Variable was reverse coded when calculating index score

### Location-specific school environment model results

Higher scores on the school physical activity environment index were associated with greater MVPA and less sedentary time at school (two matches, no mismatches; Table 2).

### Location-specific non-school psychosocial and home environment model results

Lower non-school sedentary psychosocial index (less psychosocial support for sedentary time) was related to less sedentary time at home (match). The non-school sedentary environment index was not related to any activity variables (neither match nor mismatch). The non-school physical activity psychosocial index was related to greater MVPA at school (mismatch) and “other” locations (match). Lastly, the home physical activity environment index was associated with greater MVPA at home (match) and “other” locations (mismatch) as well as less sedentary time at home (match). In summary, there were four matches and two mismatches for the location-specific non-school indices. Combined with the results for the location-specific school indices (see previous section), there were a total of six matching associations and two mismatching associations out of eight potential matches, providing support for our first hypothesis (Table 2).

### Location-general psychosocial model results

The general physical activity psychosocial index was related to greater MVPA at school and “other” locations but not related to MVPA at home (i.e., related in 2 out of the 3 locations investigated). Showing moderate support for our second hypothesis, this index was not significantly related to activity variables in all locations.

### Interactions results

**Fig. 2:**
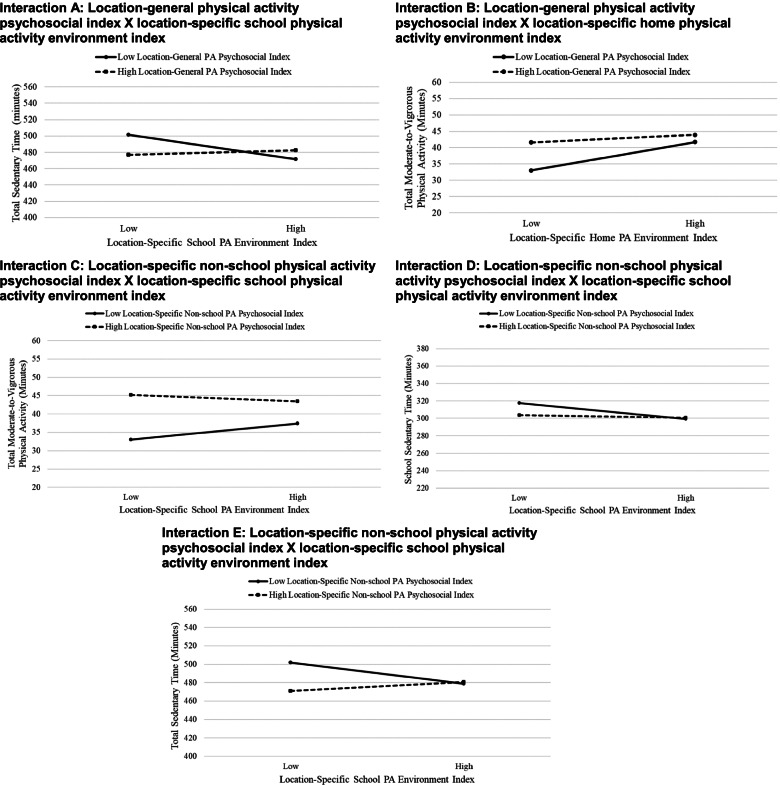
Plots of interactions with patterns depicting the benefit of having a high (favorable) value on one index when the value on the other index is low

Statistical significance for tested interactions between indices in relation to activity variables is presented in Table 4. Five interactions, labeled A-E, had a *P* value < 0.1 and were plotted. All 5 interactions involved a psychosocial X environment interaction (28% of all psychosocial X environment interactions tested). Two of these involved a location-general psychosocial X location-specific environment interaction (14% of all location-general X location-specific interactions tested), and 0 involved an interaction between location-specific indices for different locations (0% of 6 tested) (these categories were not mutually exclusive). For all 5 significant interactions, having a high value (+ 1 SD above mean) on one index was associated with greater MVPA and less sedentary time (Fig. 2). This was observed regardless of whether the index was psychosocial or environmental and regardless of the value on the other index. However, there was no/little apparent additional benefit of having a high value on both indices on activity. The effect sizes shown in the plots appear to show differences in the outcome variables, with the group that had high values on either the environmental or psychosocial index (relative to the group with low values on both) having an additional 4–12 minutes/day of total MVPA, 22–30 minutes/day fewer of total sedentary time, and 14–18 minutes/day fewer of sedentary time at school.

**Table 4:** Interactions between indices in relation to adolescents’ location-specific and overall physical activity and sedentary time^xy^

	β (SE)^z^							
	MVPA				Sedentary time			
	School	Home	Other	Overall	School	Home	Other	Overall
**Interactions**								
General physical activity psychosocial index X non-school physical activity psychosocial index	NA	− 0.02 (0.04)	0.01 (0.04)	0.00 (0.04)	NA	0.01 (0.01)	−0.02 (0.02)	− 0.02 (0.03)
General physical activity psychosocial index X school physical activity environment index	0.01 (0.04)	NA	NA	−0.03 (0.04)	0.03 (0.03)	NA	NA	^a^0.11** (0.04)
General physical activity psychosocial index X home physical activity environment index	NA	−0.05 (0.04)	NA	^b^-0.07* (0.04)	NA	0.02 (0.01)	NA	0.03 (0.04)
Non-school physical activity psychosocial index X school physical activity environment index	−0.06 (0.04)	NA	NA	^c^-0.07* (0.04)	^d^0.04* (0.03)	NA	NA	^e^0.10** (0.04)
Non-school sedentary psychosocial index X non-school sedentary environment index	NA	−0.01 (0.03)	0.01 (0.04)	0.01 (0.04)	NA	0.01 (0.01)	0.01 (0.01)	0.04 (0.03)

^a^Interaction A

^b^Interaction B

^c^Interaction C

^d^Interaction D

^e^Interaction E

^x^All models were adjusted for participant age, sex, race/ethnicity, parent education, study design factors, number of days of accelerometer wear, number of school days, and accelerometer wear time in each location;

^y^Empty cells reflect models that were not investigated due to the activity location (e.g., school MVPA) being a mismatch with the location reflected in one or both of the location-specific indices comprising the interaction (e.g., non-school physical activity psychosocial index);

^z^ Values are standardized regression coefficients (β) and standard errors (SE) representing the interaction effect, with both the independent and dependent variables standardized to have a mean of zero and standard deviation of 1; Benchmarks for interpreting the magnitude of the interaction coefficients were small (β = .10), small-to-moderate (β = .20), and moderate (β = .30); Refer to Fig. 2 for interpretation of the interaction effects

NA = Interaction not explored because location of one of the indices did not match location of the activity variable;

*P < 0.1;

**P < 0.01

## Discussion

Our exploratory study tested the value of examining location-specific psychosocial and environmental correlates of physical activity and sedentary time in adolescents. If hypothesis were supported, these findings would suggest the need for more location-tailored intervention strategies. Present findings tended to confirm hypotheses that location-specific psychosocial and environmental indices were more consistently related to location-specific activity in the matching location(s) than in the unmatching location(s). These findings also support the need to develop additional measures of location-specific psychosocial and environmental correlates of activity and evaluate their performance to confirm and extend present findings. There was a total of eight significant associations between the location-specific indices and location-specific activity variables. A majority (six of eight, or 75%) of the significant associations of location-specific variables with location-specific activity were conceptually matched (i.e., the location linked to the influencing factor matched the location in which activity occurred), suggesting little carry-over of these location-specific factors explaining behavior in other (non-matching) locations. The general physical activity psychosocial index, which was the only location-general index investigated, was related to physical activity at school and “other” locations but not home, suggesting moderate support for our second hypothesis. This finding implies that an adolescent’s general psychosocial attributes may not generalize to all locations or settings, as there might be location-specific barriers to physical activity in certain locations or different individual-level motivations or facilitators of activity that are location-specific. Researchers and interventionists can use this evidence to inform the expansion of location-specific measures of activity correlates as well as location-specific intervention strategies. Based on the present findings, we hypothesize that efforts to optimize interventions by targeting location-specific psychosocial and environmental variables across multiple locations may produce the larger impacts on adolescents’ overall activity that location-general independent variables. However, experimental studies are needed to test this hypothesis. For example, a study comparing an intervention with location-specific approaches targeting how to improve MVPA and reduce sedentary time across locations in which adolescents typically spend time compared to an intervention that did not include strategies specific to locations could yield insight on the benefits of a location-optimized intervention.

The purpose of investigating associations between the indices and overall (cross-location) physical activity and sedentary time was to contextualize the location-specific findings, as an observed association for overall activity could be driven by associations with behavior in any number of individual locations. We observed several associations between the indices and overall activity, though none of the associations with overall activity were also found with activity in all three locations. Yet, there was some evidence of carry-over across locations as indicated by the 2 mismatches. Similarly, although we found moderate support for our second hypothesis due the lack of associations between the general physical activity psychosocial index and physical activity in all three locations, the observation of associations with behavior in two of three locations (school and “other”), provides some additional evidence of carry-over across locations. The lack of association with home activity suggests there may be particular barriers to being active at home that warrant better understanding through future research. The carry-over of environmental variables into “other” locations was more surprising than the carry-over of psychosocial variables, as the latter are characteristics of the individual (who cross locations) rather than the environment (which is unique in location). However, the home physical activity environment index that exhibited some carry-over included portable equipment (e.g., bikes), which may be used away from the home environment, as described below.

Present findings regarding the value of location specificity are generally in alignment with previous studies that observed associations of environmental or psychosocial variables with physical activity only in specific locations or that were greater in magnitude for physical activity in specific locations as compared to overall (across locations) [24, 25, 47]. While most previous studies were limited to school-based or neighborhood-based activity, the present study builds on this research by including activity measures in three location categories that together comprised all of adolescents’ time and included device-based specificity for location. Taken together, these findings suggest there is generally more support for location-specific effects than carry-over across all locations, and potential carry-over is likely to relate more to psychosocial than environmental variables.

The present study expands the large body of evidence and public health recommendations [48] on the importance of the school environment in relation to school activity. In this study, the school physical activity environment index was related to both physical activity and sedentary time at school and not significantly related to MVPA or sedentary time for any other location or overall. As school environmental supportiveness of activity has been shown to vary widely across schools [49, 50], efforts should continue to improve environments and opportunities for activity at schools. Beyond the inclusion of quality physical education, recess time, and before- and after-school physical activity programming, classroom-based physical activity interventions [51] may serve as another potential way to support more physical activity and less sedentary time at school although this is not something we assessed in the current study. Previous research demonstrates that classroom modifications to bolster these behaviors include re-organizing the classroom furniture, larger classroom sizes, standing desks, and other ergonomically-friendly furniture to support reductions in sedentary time [52]. Schools may also consider identifying more indoor and outdoor facilities and amenities that would encourage adolescents’ physical activity. It is important to note that although adolescents generally have less autonomy to engage in activity at school than in many other locations, present findings suggest that psychosocial variables are also relevant to adolescents’ activity at school. This was primarily indicated by the finding that the general physical activity-related psychosocial index was most strongly related to MVPA at school, but school-specific psychosocial variables were not available.

Given that previous research showed teens generally accrued most of their activity at school and locations outside the home [36], the home appears to be a high-risk setting for inactivity and requires more attention from interventions. The general lack of meaningful associations between home physical activity and all psychosocial indices in the present study suggests that supporting adolescents to be active at home may be challenging, as there are likely additional and powerful barriers that need to be addressed. As the home physical activity environment index was the only measure statistically associated with home physical activity, interventions targeting greater access to physical activity equipment at home appear promising. The non-school sedentary environment (which could include home), which predominantly reflected screen-based technology in the present study, was not statistically related to home MVPA or sedentary time, perhaps due to the ubiquity of screens in the home. However, the association between greater non-school psychosocial supports and less home sedentary time suggests screen-based activities are important to address in health interventions. Since these psychosocial items were primarily about TV and screen time, effective intervention strategies are likely to be those that focus on an individual’s response to the screen-based environment. Present findings around both physical activity and sedentary time at home align with previous qualitative studies on how adolescents perceive less structure for physical activity at home [53] and suggest interventions should target increasing opportunities for physical activity while addressing barriers to reducing screen-based time. Effective interventions targeting these elements are likely to involve parents, as a recent meta-analysis concluded physical activity interventions targeting families more holistically produced the largest effect sizes [21]. Family intervention components could include increasing family social support, adding structure or scheduling activity, and implementing parent managed rewards [54, 55].

In contrast to the home location, adolescents’ physical activity in “other” locations was related to both general and non-school physical activity psychosocial variables. “Other” locations are likely to include neighborhoods, parks, sports areas, and friends’ and relatives’ homes, and some of these locations may have fewer screen-based barriers to activity relative to the home. Thus, interventions to improve psychosocial factors may be important for capitalizing on adolescents’ autonomy and capacity when in other locations. These interventions may also be important for supporting adolescents to seek out supportive locations for physical activity, which is in alignment with previous research [56]. Such interventions could involve teaching adolescents location-specific strategies to overcome barriers in physical activity-compromising locations (e.g., friend’s houses, after school clubs), prompting them to pursue enjoyable opportunities for physical activity when in physical activity-supporting locations such as in their neighborhoods, parks, and via active transportation, and encouraging adolescents to seek out preferred places for physical activity. Parents are likely to play an important role in influencing activity in “other” locations and could support adolescents’ autonomy and independence by providing opportunities to socialize with peers in active spaces (e.g., encouragement to participate in sports), safely and actively commuting to places, and independently doing physical activities outside the home [57–59]. The unexpected association of more supportive home physical activity environment variables with greater physical activity in “other” locations (one of two mismatches) could have been due to home physical activity equipment being used for activity outside of the home, further highlighting the importance of this facilitator.

For all significant interactions, having high supportiveness (higher values) on one index appeared to make up for low supportiveness on another index. The indices were not synergistic or additive in their associations with activity, which is often posited based on ecological perspectives on health behavior [9]. However, the compensatory nature of the interactions (i.e., one making up for a lower value in the other) suggests that successfully improving only environmental or only psychosocial variables alone can provide benefits. This does not necessary mean that only one level needs to be targeted, as targeting both levels might increase the likelihood of successfully affecting at least one level of influence. For example, when environmental modifications are not feasible or attempts have not been successful (e.g., in schools) [60], targeting psychosocial strategies may be particularly important for facilitating activity improvements. Similarly, wide reaching environmental interventions would be likely to provide benefits to all users of a setting, regardless of their psychosocial supports. However, it is important to note that interactions were only observed for a subset of the indices and activity measures (i.e., 5 of 24 [21%] of the tested interactions). It is notable that all of the significant interactions, except one, was for overall MVPA or overall sedentary time and not location-specific MVPA or sedentary time. Overall, present findings are in alignment with an accumulation of evidence showing that multilevel interventions are likely to be more impactful than those targeting only one level [9].

## Limitations and future directions

Because the original survey items did not cover all locations, as they were not originally intended to survey location-specific facets of psychosocial correlates of physical activity/sedentary time, we were unable to investigate psychosocial and environmental indices for all included locations. For example, we did not capture school-specific physical activity psychosocial variables or school-specific sedentary psychosocial or environmental variables. One potential impact of these imbalances between psychosocial and environment composites for physical activity versus sedentary time, between location-specific vs. location-general indices, and across locations, is type II error (i.e., failure to detect some associations). Since the items were developed from previous measures and were not designed to reflect the locations included this study, these items likely do not capture the specificity of locations as compared to a purposefully developed tool. It is possible that employment of established location-specific measures would reveal more associations between those location-specific measures and locational outcomes. Still, our measures were able to appropriately detect location-specific associations given our ratio of matches to mismatches. Although the location-specific variables included in the current study were derived from previously established and validated scales, the internal consistency properties or factor structure of these location-specific subscales and indices were not tested because many subscales were measured with few items. Given that few to no comprehensive survey-based measures of location-specific psychosocial variables exist, future studies should develop and psychometrically test expanded location-specific measures to improve the measurement of each location-specific construct and capture more locations with greater comprehensiveness and specificity. Location-specific measures that are more comprehensively developed and psychometrically established across diverse samples would allow for more robust research in this area. Methods focused on understanding interactions between adolescents and contexts, such as Ecological Momentary Assessment, would also be useful for improving understanding of location-general vs. location-specific correlates of activity.

The effect sizes as indicated by the standardized regression coefficients for associations between some indices and location-specific sedentary time were small, as low as 0.03. Although such small associations may not be clinically meaningful for an individual adolescent, location-specific associations could add up to create larger impacts at the day level (across locations) and population level.

With regards to the behavioral specificity of the associations, the physical activity indices were generally more consistently and strongly related to the greater physical activity than to less sedentary time, with a few exceptions. The moderate correlations between MVPA and sedentary time within locations and overall may have slightly impacted results such that greater MVPA in a location could have displaced sedentary time in that location. However, we only observed two associations when a location-specific physical activity index was significantly related to that location’s sedentary time. Although there appears to be some interconnectedness between physical activity and sedentary time (as indicated by these correlations), present findings are generally in agreement with prior evidence showing a person can be both highly physically active and highly sedentary (i.e., less time in light activity) [7, 8] and suggest that interventions aiming to impact both behaviors need to target each with specificity and across multiple locations. The present study only included two sedentary indices as compared to four physical activity indices, so although the indices showed similarly few associations with both physical activity and sedentary time (one association for each behavior), we are not able to draw strong conclusions around this finding. As indicated earlier respective to the home location, correlates of sedentary time are likely to be complex, which could explain the general lack of associations observed between the sedentary psychosocial/environmental indices and sedentary time. Other variables not measured in this study should be investigated in future studies, such as psychosocial supports for reducing sedentary time at school and availability of movement-supporting furniture at home and school [61, 62] to more comprehensively assess sedentary time across and within locations and would build upon current findings.

Although care was taken to address GPS satellite interference (e.g., large buffers, up to 2 epochs allowed outside of the location before breaking up a bout), errant GPS scatter may have erroneously linked epochs of activity with the wrong location. Since the “other” location was broadly defined, limited inferences can be made about how location-specific psychosocial and environmental variables relate to physical activity and sedentary time in specific locations within the “other” category (e.g., parks, friends’ homes). We were unable to parse out these specific locations within the “other” category due to the use of historical data in the current study as the data needed to parse these locations were not collected in the original TEAN study. However, a previous study showed a large portion of the “other” category comprised the home and school neighborhood (including active transport) [63], specifically 53% of “other” location MVPA occurred in the home or school neighborhood) [15]. This suggests that intervention strategies for the “other” location might include encouraging more physical activity near the home and school neighborhoods. These locations might be more convenient for adolescents compared to a park or recreation facility and therefore future interventions should capitalize on these places for sustaining MVPA. Still, future research should aim to parse out the specific locations within the “other” category. Present findings were cross-sectional and do not suggest these location-general or location-specific psychosocial or environmental variables caused physical activity or sedentary time. Instead, they highlight important correlates of activity that should be tested in prospective and intervention studies. Due to the large number of statistical tests conducted, we would expect some findings to be significant simply by chance. Therefore, we focused more on interpreting *patterns* across associations (e.g., this was the focus of our hypotheses) than individual associations, which is particularly appropriate for a preliminary study such as the present one.

## Conclusion

A majority of the location-specific variables investigated were related to activity in the matching location and not in non-matching locations, providing some support for the concept of location-specificity among correlates. Assessment of location-specific psychosocial and environmental variables can offer a more comprehensive understanding of adolescents’ activity within a variety of locations, further tests of location-specific associations have the potential to inform more-tailored intervention strategies for improving and sustaining physical activity and sedentary behavior. Interventions that are multilevel, location-specific, and target multiple locations may have greater impacts than interventions that target only single locations, general variables, or a single level of influence.

## Supplementary Information

**Additional file 1.** PALMS User Guide. Includes a technical guide regarding the integration of GPS and accelerometer information.

**Additional file 2: Supplementary Table 1.** Items categorized by scales and indices. Includes table which was too long to submit in the manuscript text.

**Additional file 3: Supplementary Table 2.** Descriptive statistics for psychosocial and environmental subscales comprising the indices. Includes table as supplemental information.

**Additional file 4: Supplementary Table 3.** Partial correlations between physical activity and sedentary time within locations. Includes partial correlations for outcome variables as supplemental information.

**Additional file 5.** STROBE Statement—checklist of items that should be included in reports of observational studies. STROBE Checklist for the study.

## Data Availability

The data and research materials can be obtained by contacting the last two authors of this paper (JFS & JAC).

## Abbreviations

GPS: Global positioning system
PA: Physical activity
MVPA: Moderate-to-vigorous physical activity
TEAN: Teen environment and neighborhood
