# Supplementary material for: Location-specific psychosocial and environmental correlates of physical activity and sedentary time in young adolescents: preliminary evidence for location-specific approaches from a cross-sectional observational study
Source: Int J Behav Nutr Phys Act. 2022 Aug 26;19:108. doi: 10.1186/s12966-022-01336-7 (PMC9419353; doi:10.1186/s12966-022-01336-7)
Supplement: Supplementary file 1 — Additional file 1. PALMS User Guide. Includes a technical guide regarding the integration of GPS and accelerometer information. [file 12966_2022_1336_MOESM1_ESM.pdf]

PALMS

Personal Activity Location Measurement System

USER GUIDE

December 1, 2011

UCSD

CW PHS

CENTER FOR WIRELESS & POPULATION HEALTH SYSTEMS

Table of Contents

1 Introduction to PALMS 1
1.1 Data Merging, Visualization and Exporting 1
1.2 Calculations and Algorithms 2
1.3 PALMS Community and Wiki 2
1.4 Technical Support 2
1.5 Quick Start Guide 2
2 Devices Supported 3
3 Data Formats 4
4 Time Synchronization 5
5 Before Using PALMS 6
6 PALMS Terminology and Acronyms 7
7 User Access to PALMS 9
7.1 Logging in to PALMS 9
8 The PALMS Workflow 11
8.1 Creating a Study 11
8.2 Adding Participants 16
8.3 Adding Datasets 17
8.3.1 Visualizing Data 20
8.4 Calculations 21
8.4.1 Saving Parameter Protocols 22
8.4.2 GPS, Activity, HR – Process and Merge (R2) 22
8.4.2.1 General Information Parameters 23
8.4.2.2 GPS Parameters 23
8.4.2.3 Accelerometer Parameters 31
8.4.2.4 Heart Rate Parameters 33
8.4.2.5 Energy Expenditure (EE) Estimate Parameters 35
8.4.2.6 Merge Options 36
8.4.3 Daily Summary v. 1.0 38
8.5 Viewing/Exporting Results 39
APPENDICES 42
Appendix A: Creating Data Table Files With ActiGraph Data Files 43
Appendix B: Configuration and Download Procedures for Qstarz Devices 46
Appendix C: Guidelines for Using Google Earth with PALMS Output 56
Appendix D. Importing PALMS CSV Output into ArcGIS (by: Jasper Schipperijn, PhD) 60
Appendix E. Examples of Participant Instructions (Wear and Charging) 68
Appendix F. PASTA Protocol 72
Appendix G. Parameter Quick Reference Guide 76
Appendix H. PALMS CSV Output Dictionary 81

1 Introduction to PALMS

PALMS (Personal Activity Location Measurement System) is an easy-to-use web-accessible service for measuring physical activity. Its main function is to merge and process time-stamped data from devices such as accelerometers, heart-rate monitors and global positioning system (GPS) instruments.

PALMS was developed by researchers at UCSD in the Center for Wireless and Population Health Systems, Calit2, and the Department of Family and Preventive Medicine, School of Medicine. PALMS was funded by the National Institutes of Health, Gene-Environment Initiative, (Grant 1 U01 CA130771). The intent is to improve measurement of physical activity in time and space so as to better inform research—both individual and collaborative—in exposure biology.

PALMS Overview

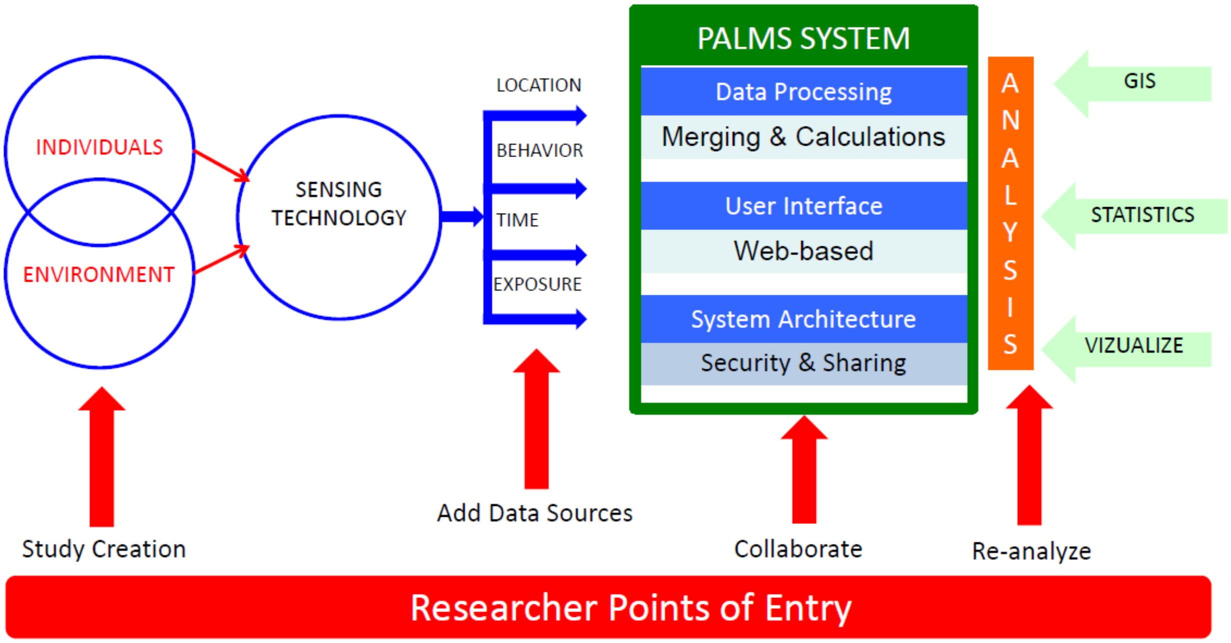

1.1 Data Merging, Visualization and Exporting

The main function of PALMS is to merge and process time-stamped data. PALMS is primarily designed to accept and process time-stamped data from accelerometers, heart rate monitors, and in the future, other data sources, such as data from wearable environmental sensors, will be included. PALMS is designed to be flexible so that data from new devices can be added to PALMS at minimal cost.

In addition to processing raw time-stamped files, PALMS also aggregates epoch-based data into more manageable files, for example, by day, participant or event. PALMS output files can be exported into other software packages, such as SPSS, ArcGIS, and in KML files for visualization.

1.2 Calculations and Algorithms

One of the features of PALMS is its calculation function, which identifies variables of interest, including trips, transportation modes (e.g., walking or vehicular), activity intensity, and GPS data points. PALMS allows users to select parameter settings to meet their specific study needs. This enables researchers to assess the impact of various parameters on their results, save parameter protocols for replication in other studies, and share them with researchers interested in advancing the field of physical activity measurement.

Note: The performance of PALMS algorithms to accurately detect trips, transportation modes and location is currently being assessed in a validation study supported by the NIH. PALMS is also being developed in collaboration with researchers at UCSD with technologies and processes to ensure data security and policy-driven data sharing.

1.3 PALMS Community and Wiki

PALMS is currently freely available online to those with a valid username and password. As of September 1, 2011, there are 33 PALMS users in seven countries. PALMS is currently deployed in 15 studies. For additional up-to-date information and/or to share experiences and learning, please visit the PALMS Wiki at:

http://ucsd-palms-project.wikispaces.com/

1.4 Technical Support

In addition to this user guide, materials are available to help researchers work with different devices, collect data from participants, and test PALMS parameters. Technical support and topic expertise is also available. Please contact PALMS staff at: palms@ucsd.edu. Initial consultation is provided at no cost with additional services available at reasonable prices.

1.5 Quick Start Guide

The quick start guide is a concise overview to using PALMS. You can find a copy of the quick start guide at: https://palms.ucsd.edu:8443/QuickStart/

There is also a link through our Wiki page: http://ucsd-palms-project.wikispaces.com

2 Devices Supported

PALMS supports the following devices:

1. Accelerometers
   (a) ActiGraph GT3X+*
   (b) ActiGraph GT3X
   (c) ActiGraph GT1M
   (d) ActiCal
2. Heart rate and motion devices
   (a) ActiTrainer (uni- and tri-axial)
   (b) ActiHeart
   (c) Zephyr Bioharness
3. GPS devices
   (a) Qstarz GPS devices
   (b) USGlobalSat DG-100
   (c) Any GPS that exports a GPX file

* Data from the GT3X+ can only be imported to PALMS, if it is re-integrated to an epoch and converted to a data table. Raw acceleration data from the GT3X+ cannot be imported at this time.

We are regularly adding new devices, so if yours is not listed, check the Wiki. If your device is not listed in the Wiki, please contact us to discuss your requirements.

3 Data Formats

Various formats are available for different data types. Outputs from most devices can be saved as **comma-separated values (CSV)** data files. This is the primary format for importing data into PALMS.

The ActiGraph accelerometers and heart rate monitor data must be downloaded and converted to a “data table format” to be uploaded to PALMS. The data table format assigns a date and time stamp to each record. To convert DAT or AGD files to the data table format in ActiLife 4 or 5, follow the procedure described in Appendix A.

Most GPS devices can be exported in GPX format that is readily imported to PALMS. For GlobalSat DG-100 devices, you can also import the GSD and the CSV file formats. For Qstarz devices, additional data such as satellite information—used by PALMS to make indoor and outdoor classifications—can be saved in the CSV format using the Raw Data Manager in the Qstarz software. For further details, please see configuration and download procedures in Appendix B.

4 Time Synchronization

A key feature of PALMS is its ability to synchronize time-stamped data streams from multiple devices recording at different epochs. PALMS merges these data streams into one merged file for calculations and analysis.

For optimal synchronization, the clocks of all devices must be synchronized before deployment.

GPS data loggers obtain their clock from GPS satellites, which are synchronized to atomic clocks at the U.S. Naval Observatory. This master clock is also the reference clock for all Internet timeservers. This time is reported in UTC (Coordinated Universal Time).

Time from other devices such as the ActiGraph accelerometers are synchronized to the computer clock each time they are initialized.

> ***Important:*** *To ensure that the PALMS algorithms properly merge your data you MUST synchronize your computer's clock to a UTC clock.*

Users can download the NISTime 32 program from the National Institute of Standards and Time (NIST) website (http://www.nist.gov/pml/div688/grp40/its.cfm). This program allows users to check their computer clocks against Internet time and adjust as necessary.

You can also manually synchronize your computer clock to minutes and seconds found at: www.time.gov.

International users can identify their national time laboratories on the Bureau International des Poids et Mesures website (http://www.bipm.org/en/scientific/tai/). These time laboratories have national clocks to which you can synchronize your computer.

5 Before Using PALMS

PALMS will perform optimally if the data are collected following standard procedures and best practices. While not all such procedures are well established, we have provided guidelines to help users collect the best possible data. These guidelines and protocols can be reviewed in the Appendices, which include the following documents:

- Creating Data Table Files with ActiGraph Data (Appendix A)
- Configuration and Download Procedures for Qstarz GPS Devices (Appendix B)
- Guidelines for Using Google Earth with PALMS Output (Appendix C)
- Importing PALMS CSV Output into ArcGIS (Appendix D)
- Example Participant Wear Instructions (Appendix E)
- Physical Activity Spatial–Temporal Analysis (PASTA) Protocol (Appendix F)
- Parameter Quick Reference Guide (Appendix G)
- PALMS CSV Output Dictionary (Appendix H)

6 PALMS Terminology and Acronyms

This section defines terms as they are used in the PALMS software. Please note, these terms may be defined differently from those commonly used in the scientific community.

AGD - AGD files are database files, and is the native file format for ActiLife operations and tools. Although the file cannot be processed in ActiLife, the AGD format can be exported to CSV or DAT files through the ActiLife import/export tool or AGD viewer tool.

BPM - Beats per minute, referring to pulse or heart rate.

Calculations - Calculations are applied to the raw datasets for one or all participants within a study. Calculations typically clean, categorize and merge datasets from multiple devices to create a results set Calculations contain options and parameters that determine how the calculation works. These defaults can be changed by the user and saved as Protocols.

CSV - CSV (comma-separated values) files can be imported into spreadsheets (Excel), statistical programs (SPSS, SAS) or GIS programs (ArcGIS) for display and analysis.

DAT - A file with the DAT file extension is a data file.

Datasets - Data from devices is uploaded to PALMS via a web page and stored into datasets. A dataset contains data for a single participant for a single device recorded over a given period. A participant may have multiple datasets for each device, as long as the datasets do not overlap in time.

Devices - Devices collect data about study participants. Typical devices include GPS data loggers, accelerometers, and heart rate monitors.

EE - Energy expenditure.

GSD - General (or generic) station description. A GSD file is a readable ASCII text file and contains both general and device-specific specifications for communication and network configuration.

GPS - Global Positioning System. A worldwide navigational and surveying system based on the reception of signals from an array of orbiting satellites.

GT3X+ - The model number of an AcitGraph activity monitor that provides physical activity measurements such as activity counts and vector magnitude, energy expenditure, steps taken, activity intensity levels, METs, etc. This device collects data in raw format at a user-specified sample rate up to 100 Hertz. Filtering and epoch selection are performed after data is collected, allowing users to process datasets multiple times at different epoch selections even after a study has ended.

GPX - GPS eXchange. A device-independent data format used for GPS navigation devices. It is used for exchanging GPS data between programs and sharing data with other users.

HR - Heart rate.

HRM - Heart rate monitor.

KML - KML (keyhole markup language) files can be viewed in Google Earth. They are visual representations of the data, i.e., in a map.

LAT – Latitude.

LON – Longitude.

LOS - A loss of signal (los) is a period of time during which a GPS device could not fix its location; usually caused by being in a building or tunnel.

MVPA - Moderate-to-vigorous physical activity.

PASTA - Physical Activity Spatial – Temporal Analysis.

Participants - Study participants (sometimes called subjects) typically wear one or more devices for several days. At the end of that period the data is downloaded from the device and uploaded to the PALMS server via a page on the PALMS web site.

PI - Principal investigator. The scientist in charge of an experiment or research project.

Protocols - The protocol contains the options and parameters used by a calculation. Protocols are saved with each results set, enabling the user to see details about how the result was generated.

Results sets - Results sets are generated each time a calculation is executed. It contains either the resulting data or an error message if the calculation fails. Results sets can be exported as CSV or KML files. They can also be deleted.

SNR – Signal-to-noise ratio.

Study - PALMS is organized by studies. A study contains all the device and participant data as well as the results of calculations performed on that data and the analysis protocol. If you need to apply different analysis protocols for multiple participants (e.g. different accelerometer cutoffs by age group for children), you should create a study for each analysis protocol. This will allow you to run one analysis protocol for each study instead of applying individual protocols for each participant.

Users - Users refer to people who access the PALMS web application. Study participants (or subjects) are not considered users. Users are assigned to roles, each with a different level of access and privileges (see next section).

UTC - Coordinated Universal Time (abbreviated UTC) is the primary time standard by which the world regulates clocks and time. Computer servers, online services and other entities that rely on having a universally accepted time use UTC for that purpose.

WET - Work energy theorem.

7 User Access to PALMS

To gain access to PALMS please contact us at: mailto:palms@ucsd.edu. You will be asked to provide contact and study information to create a user account. You will then be given a username, password, and a study group.

PALMS users are currently organized into study groups. A study group is defined as those users who collaborate on the same project. Within a study group multiple studies can be defined; but all members of the same group have access to these studies. PALMS users can also have access to multiple study groups.

For example, if Sarah, Ann, Jake and Matt are all working on the same study, they will be assigned to one study group in PALMS (e.g., Center1). However, if Sarah and Matt are also working on another study to which Ann and Jake should not have access, then Sarah and Matt will be also be part of another study group (e.g., Center2). As members of both study groups Sarah and Matt will be allowed to work in either study group, but Ann and Jake will be restricted to Center1 study group.

At the moment users are given privileges to all functionalities in PALMS. This means that any user can create studies, add and delete data, run calculations, export results, etc. In the future, however, the user in the study group who has the role as the PI (principal investigator) will set the access to various functions in PALMS and can restrict functions, e.g. disallow delete functions.

PALMS administrators have access to all studies. These administrators can help to troubleshoot any problems in uploading, or downloading data and running calculations. PALMS administrators have completed CITI Ethical training and their certificates can be provided on request. The administrators will not copy or use your data for any purpose without permission.

7.1 Logging in to PALMS

When you receive your username, password and study group, you can log into PALMS at:

https://palms.ucsd.edu:8443/PALMS/

Enter your username and password on the main page as shown below.

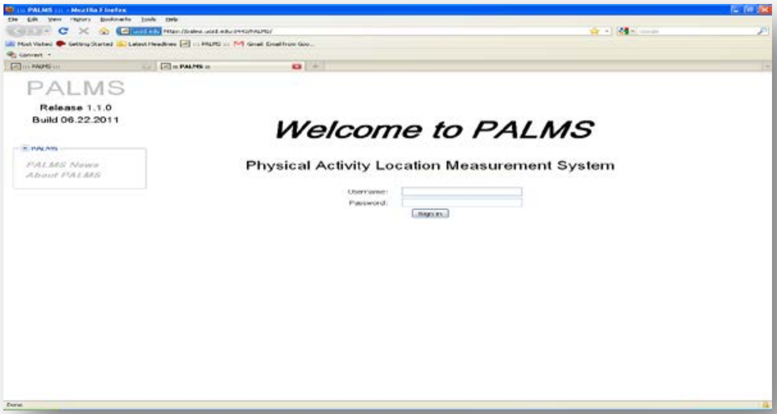

When your username and password is authenticated, you'll be taken to the study list, which shows all the studies that you have permission to access.

> ***Note:*** *When you log in to PALMS for the first time, your study list may be empty since no studies have been created. The procedure for creating a study is discussed in Section 8.1.*

Click on the study's name to open it. All further actions will affect the selected study only. If you have multiple studies, you can move between studies by clicking on the Change Study link on the left side of the screen.

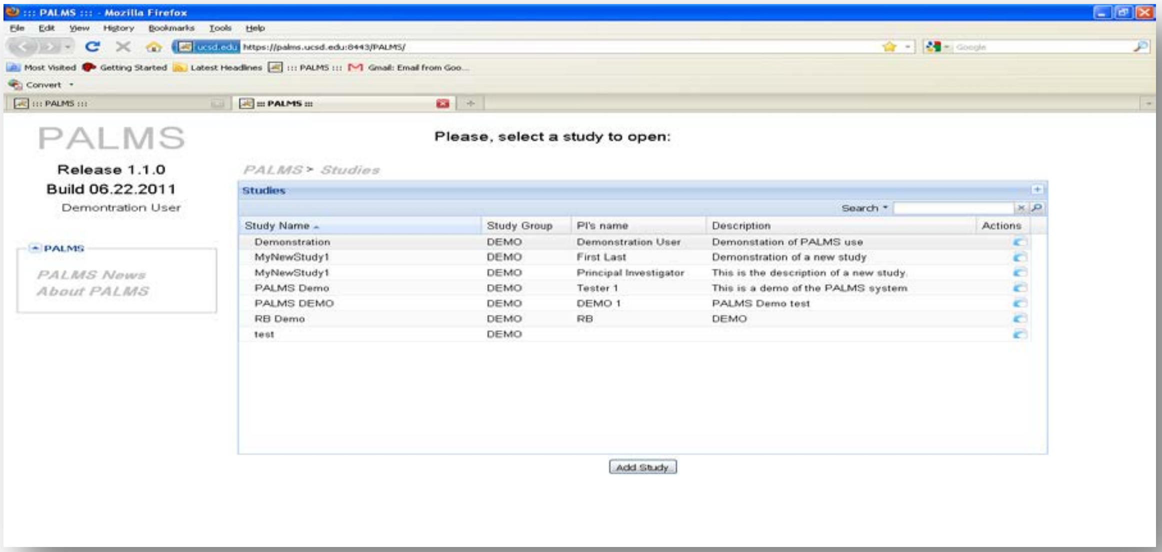

8 The PALMS Workflow

The sequence of steps for using PALMS is:

1. Create a new study.
2. Configure the study by selecting devices and calculations.
3. Enter the study participant.
4. Upload datasets (collected by the devices) for each participant.
5. Set up a calculation protocol and run the calculation (save protocol if desired).
6. View results within PALMS.
7. Export the results to other software packages for further analysis (optional).

These steps can be completed *following* data collection in the research study or completed as part of an ongoing process *during* data collection. When using PALMS during data collection, steps 9.1 and 9.2 are completed only once at the beginning of the research study. Step 9.3 is completed as participants are enrolled. Step 9.4 is begun when the devices are retrieved from the participant. Steps 9.5–9.7 are done as an ongoing process as datasets become available and the results analyzed.

8.1 Creating a Study

1. Log in to PALMS as described in Section 7.
2. When your username and password are authenticated, a study list appears, presenting all studies that you have permission to access.
3. To create a new study, click the Add Study button at the bottom of the page.

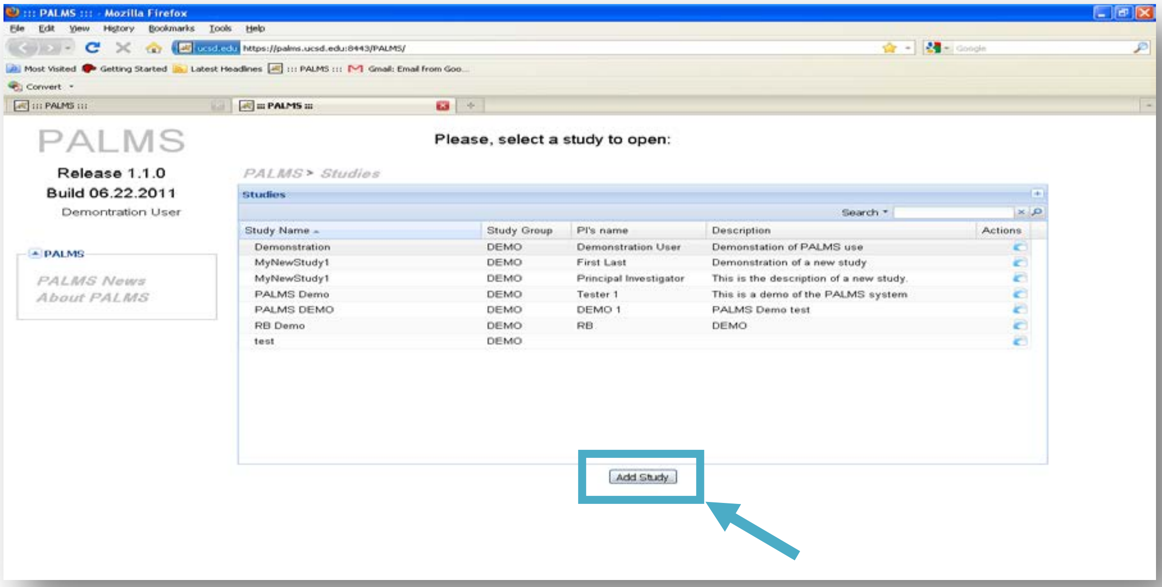

4. In the Study Name field, enter a name for your study.
5. In the PI Name field, enter your name or the name of the study's PI (principal investigator).
6. Optionally, enter the dates your study starts and ends.

7. Enter a short description of your study.
8. Select the study group to which you have been assigned.
9. Select the default time zone for the study. This should be the time zone where the majority of your study's participants live. Selecting the appropriate time zone is important because PALMS converts UTC time from GPS devices to local time and displays data in local time (see Section 4).
10. Click the Save button.

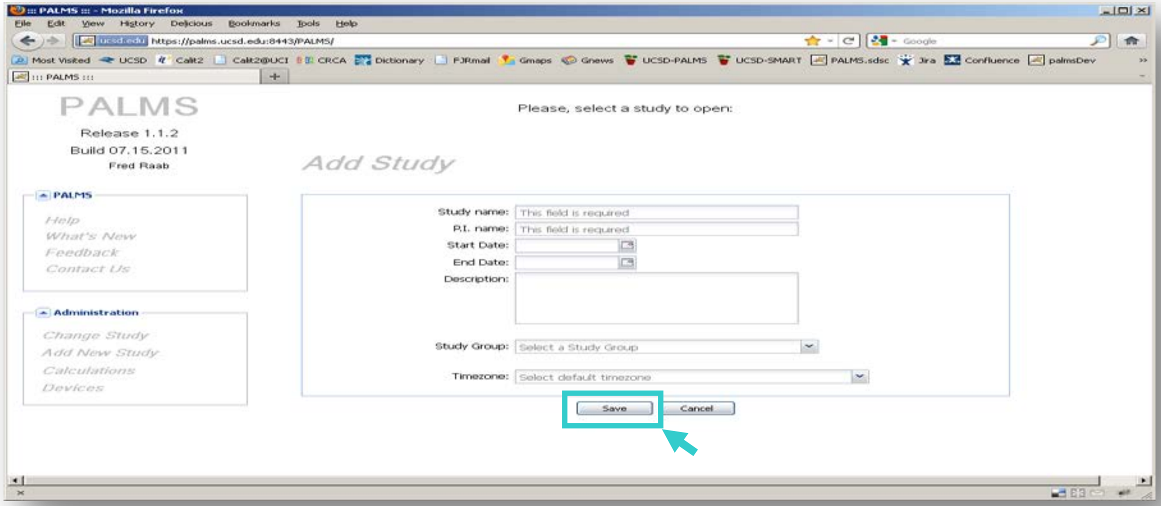

11. Click Study from the top menu bar to define devices and calculations for your study.

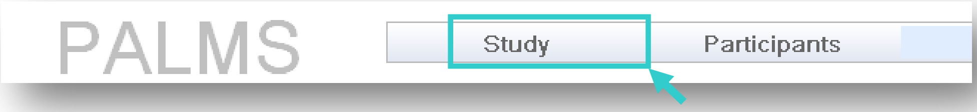

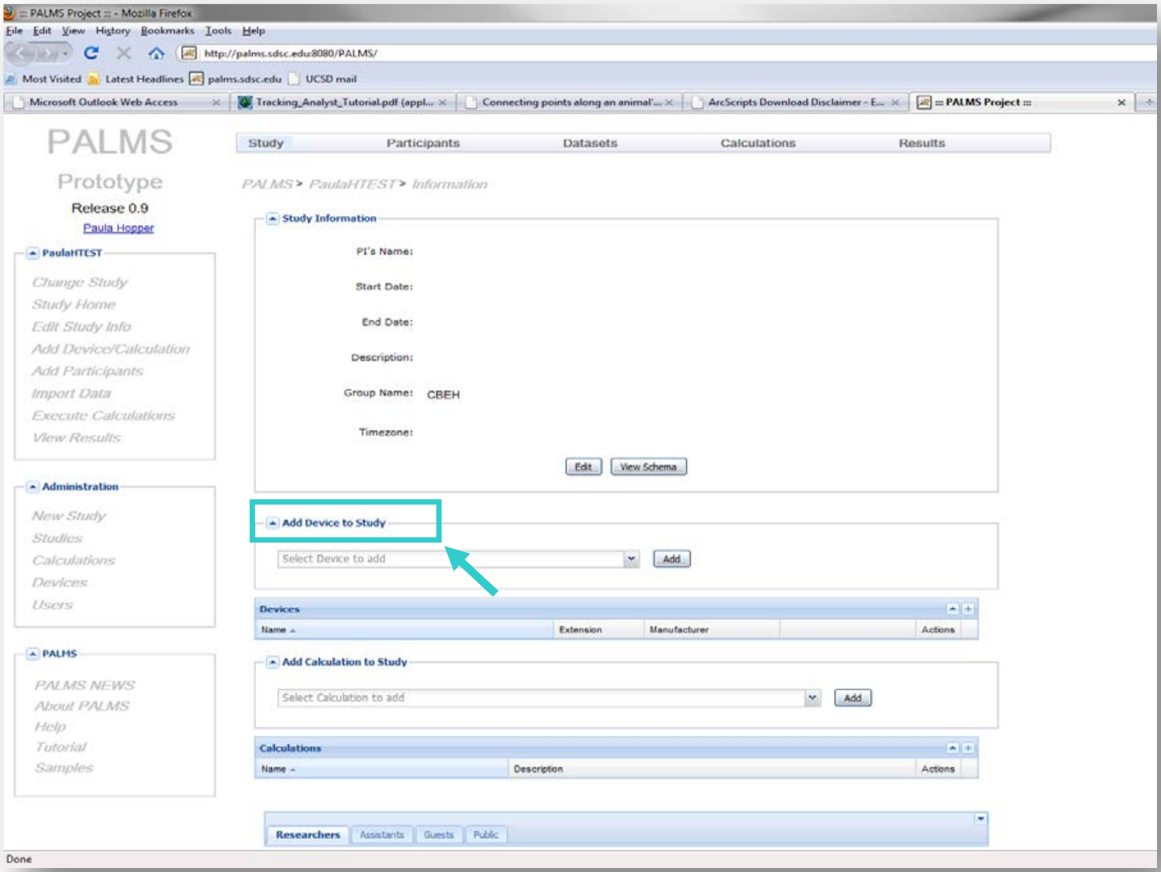

12. In PALMS, devices must be defined in the Device table on the Study page before any data is uploaded. Select the device formats you wish to add according to Table 1 below. Click the Add button to add the device.

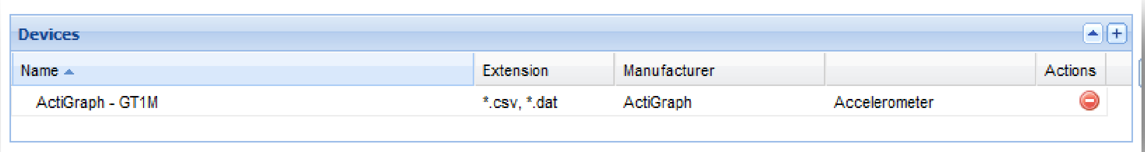

Table 1. Device formats for GPS and activity devices

**Devices**	**PALMS-Compatible Device Formats**
ActiCal	ActiCal (*.AWC*.CSV)
GT1M & GT3X	ActiGraph – GT1M (*.CSV, *.DAT)<br>ActiGraph – GT1M – Date Format = dd/mm/yyyy (*.CSV, *.DAT)[a]<br>ActiLife 5 Data Table (*.csv)<br>ActiLife 5 Data Table – Date Format = dd/mm/yyyy (*.CSV)[a]<br>ActiGraph – MeterPlus (*.mpd)
GT3X+[b]	ActiLife 5 Data Table (*.csv)<br>ActiLife 5 Data Table – Date Format = dd/mm/yyyy (*.CSV)[a]<br>ActiGraph – MeterPlus (*.mpd)
ActiHeart	ActiHeart (*.csv)
ActiTrainer	ActiTrainer (*.CSV, *.DAT)<br>ActiLife 5 Data Table (*.CSV)<br>ActiLife 5 Data Table – Date Format = dd/mm/yyyy (*.CSV)[a]
DG-100	GPS – DG-100 (*.GSD)<br>GPS – GlobalStat CSV Format (*.CSV)<br>GPS (.GPX format) (*.GPX)
Qstarz GPS	GPS = Qstarz (raw data format) (*.csv)<br>GPS (.GPX format) (*.GPX)
All Devices	All Devices (*.*)[c]

[a] ActiLife 4/ActiLife 5 data table using an international data format
[b] GT3X+ raw files must be re-integrated to an epoch and converted to a data table before uploading.
[c] Used to import multiple devices at a time. Specific device formats must be added to the device table.

***Recommendation**: Use the ActiLife 5 data table uploading GT1M, GT3X, GT3X+ and ActiTrainer data.*

13. Added devices will be listed. To add multiple devices click the + button in the top right corner of the Device table and click Add. To remove a device, select it and then click the delete icon. 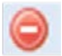

14. Next, define the calculations to be used in your study. Select the desired calculation from the dropdown menu (see Table 2 for brief descriptions) and click Add. You can select more than one calculation, and you can add/remove calculations at a later time. The calculations selected here are executed from the top menu bar under Calculations.

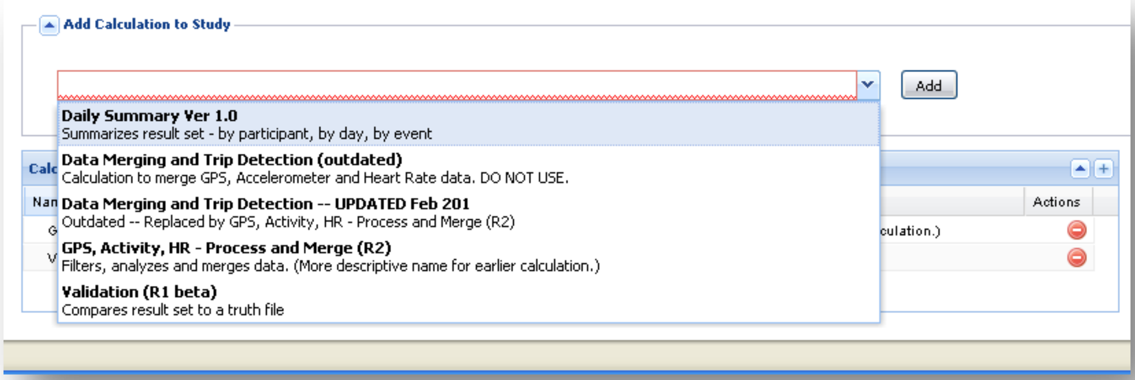

Table 2: Calculation descriptions

Calculation	Description
GPS, Activity, HR – Process and merge (R2)	This is the main calculation in PALMS. It processes and merges GPS, activity and heart rate data. It filters GPS data, detecting trips and location, and classifies locations as indoor/outdoor (optional). It also classifies activity intensity and detects bouts of activity.
Data Merging and Trip Detection – UPDATED Feb 2011	This is an older version of the main calculation. (DO NOT USE)
Data Merging and Trip Detection (outdated)	This is an older version of the main calculation. (DO NOT USE)
Daily Summary Ver. 1.0	This calculation uses results sets from the main calculation as an input and aggregates this result to calculate total number of trips in a day, modes of travel and other variables across each wear day for each participant.
Validation Calculations	These are calculations under development for use by PALMS staff in their validation studies.<br>They are available for use by the general PALMS community to compare the results generated by different protocols.

***Note**: Details about the most current calculations, their inputs, outputs and parameters can be found on the PALMS Wiki. Older versions of calculations are stored in the system so researchers who used these calculations in a previous study can re-run them at any time if needed.*

8.2 Adding Participants

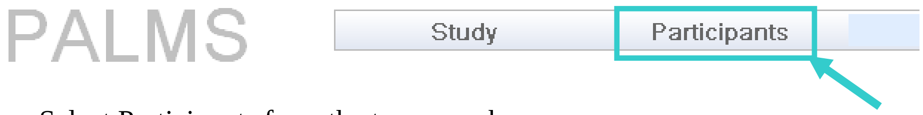

1. Select Participants from the top menu bar.

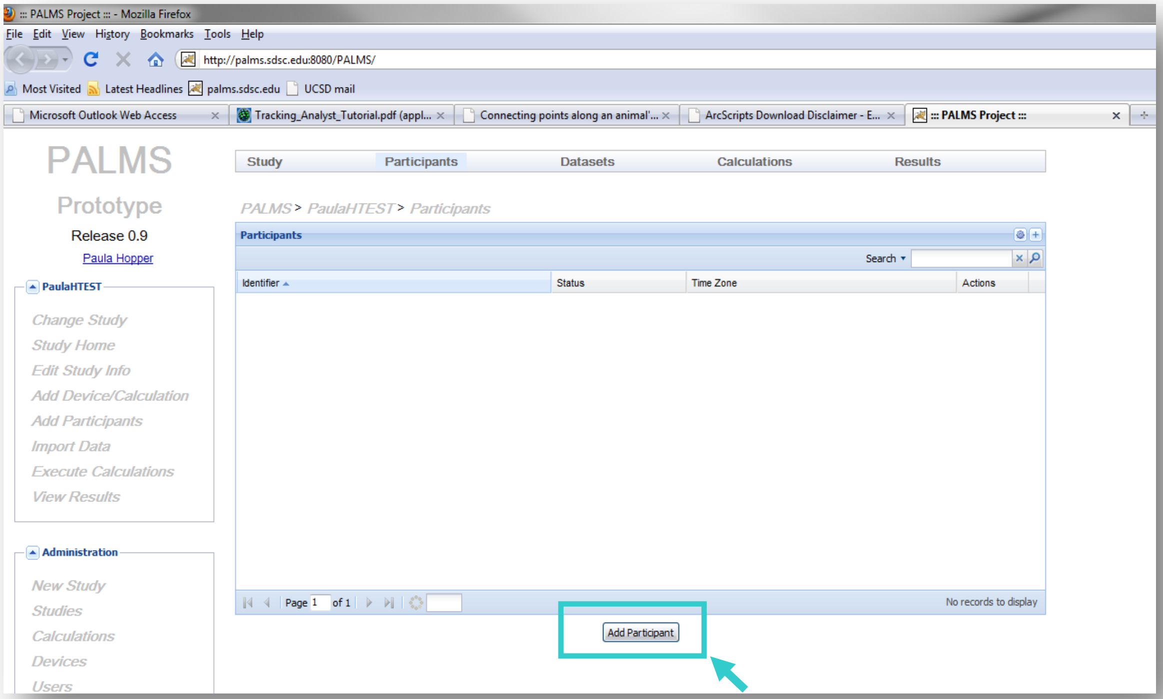

2. Select Add Participant.

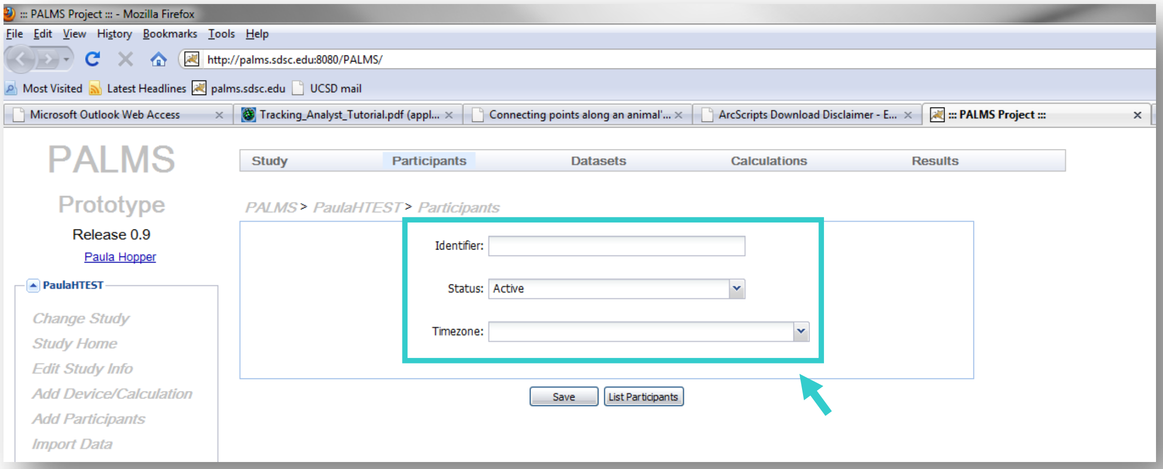

***Note**: Actions link to specific functions in PALMS and only appear when participants have been added. For example, you will not be able to upload a dataset without first adding the participant to the study.*

3. Add the participant information, according to the descriptions below:

   Identifier: All alphanumeric characters allowed. To protect participant confidentiality, it is recommended that you do NOT use the participant's name.
   Status:
   Inactive – Participant is no longer active in the study. No data has been uploaded for this participant.
   Active – Participant is active in the study.
   Completed – All data has been successful uploaded for this participant.
   Test – This participant is a test participant (typically a staff member) and the data should not be included in the final analysis.
   Time zone: The time zone where the data was collected.
   Actions: *(buttons)*

   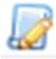 Edit participant information

   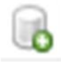 Import participant data

   ***Note**: The Status field is for the researcher's convenience and is not currently used by the calculations, i.e., you cannot select participants by their status.*

4. Click the Save button.
5. After saving the participant, the Table of Participants will open. You can add more participants or edit information about a previously added participant.
6. To add another participant, click the Add Participant button and repeat steps above.
7. To edit information, click the Edit Participant button and make changes to the identifier, status or time zone.

8.3 Adding Datasets

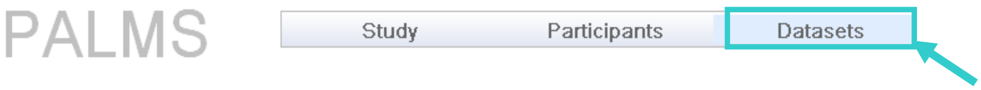

***Note**: At this time, PALMS does not allow for batch uploading of data files.*

1. To add datasets, you can either select the Dataset tab from the menu bar, or use the Import Dataset button in the participant table. When you use the Import Dataset button, the participant will be automatically populated in the Import Datasets section.
2. The dataset table has the following headers:

   Participant: Participant identifier.

   Device: Device type.

   Date Imported: Date the data was imported.

Starting Date: Starting date of the data file.

Ending Date: Ending date of the data file.

# of Samples: Total rows of data collected.

Status:

Available – PALMS has added the dataset to the database and it's ready for a calculation.

Inserting – PALMS is currently adding the dataset to the database (use the refresh button in the upper right corner of the dataset table to update the data and see progress in the status).

Rejected – There was an error in the file format or an invalid value. Double click on the word to see greater detail and contact the PALMS administrators for help troubleshooting.

Actions: *(buttons)*

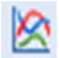
View data as points on a map (GPS) or a timeline (Activity Data).

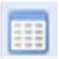
View data as a table.

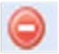
Delete datasets.

3. Select a participant and device from the dropdown menu in the Import Datasets section, and then click on the Import Datasets button.

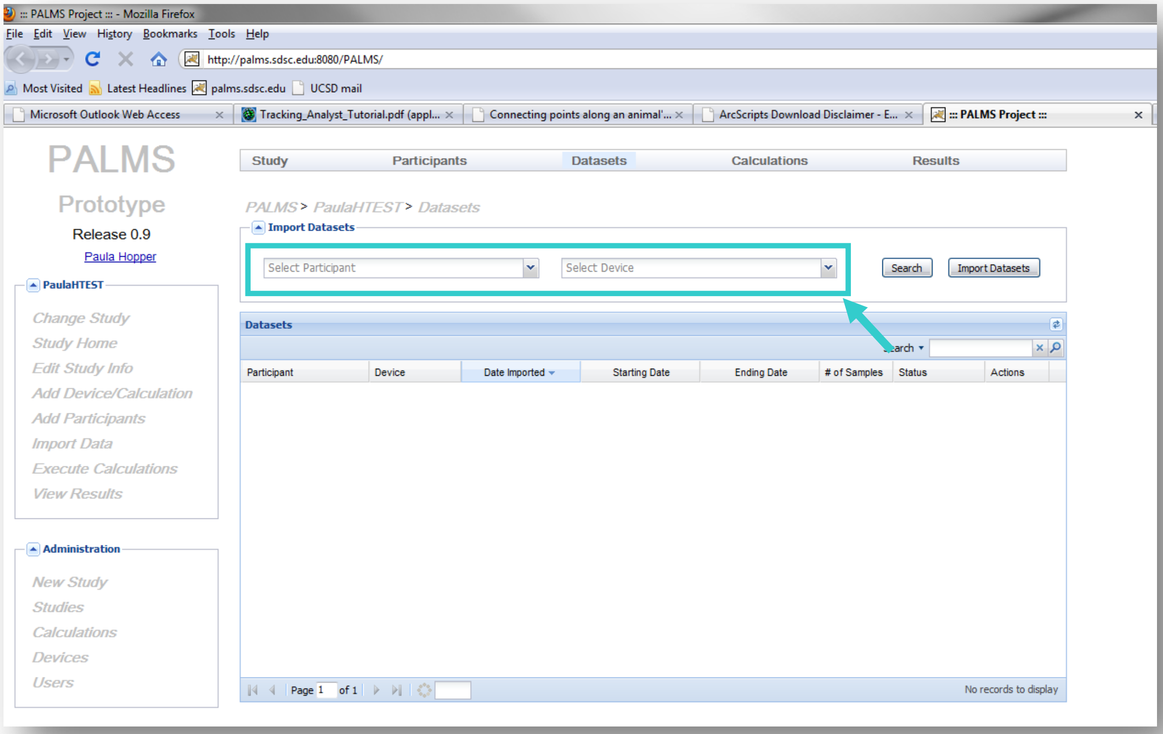

4. An Importing Files – Participant dialogue box will appear. Click on the Add button in the bottom left corner of the new box.

5. Browse and open the appropriate file for the participant and device type and then click the Upload button.

Tip: *When uploading files, be sure to check the ID in the filename with the participant ID in the dialogue box title to make sure you are uploading the appropriate files.*

6. When the file is uploaded, the Notes header will read Upload Successful. Click the Close button to close the Importing Files dialogue box.

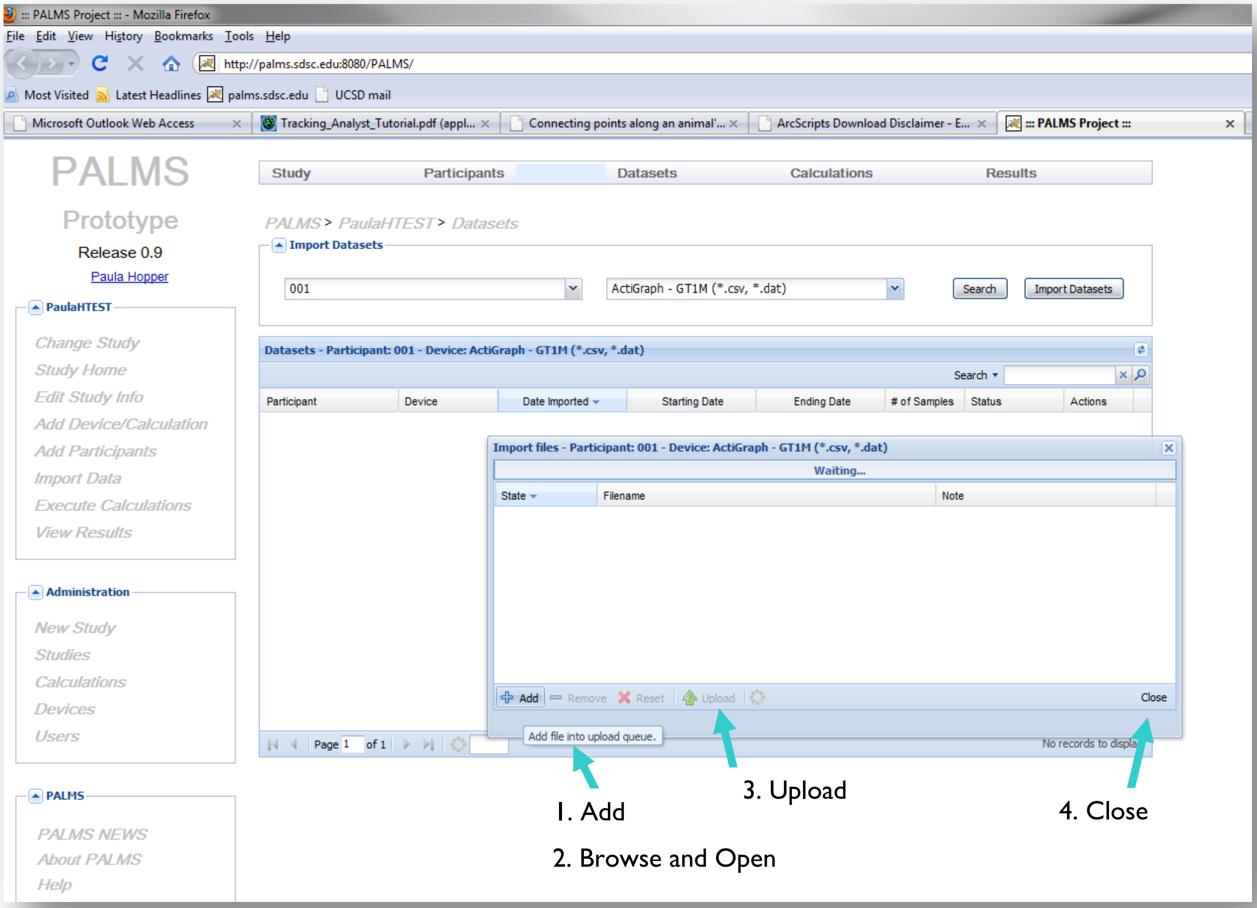

7. After closing the Importing Files dialogue box, check the status of the file just imported. The status can be Available, Inserting, or Rejected (see table above for descriptions).

Tip: *Once you've uploaded the GPS and activity files for a participant make sure to check that there is an overlap between the start and end dates and time of the two files. If there is no overlap, you may have uploaded the wrong file.*

8. Repeat the steps to add datasets until all the datasets (i.e. participant data files) for the study are uploaded.

8.3.1 Visualizing Data

The visualization action button 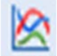 in the dataset table will produce visual data either as a map for GPS or as a timeline for activity data.

GPS data visualization:

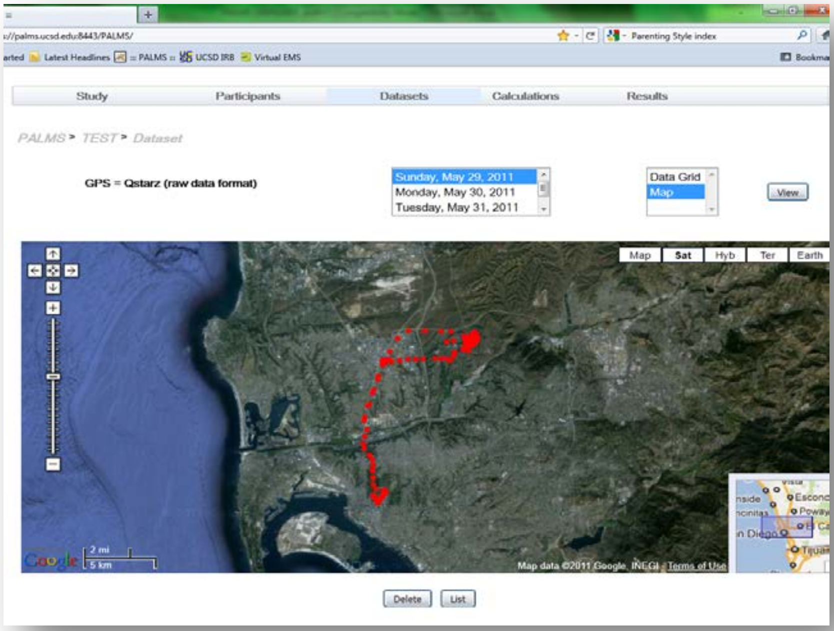

Activity data visualization:

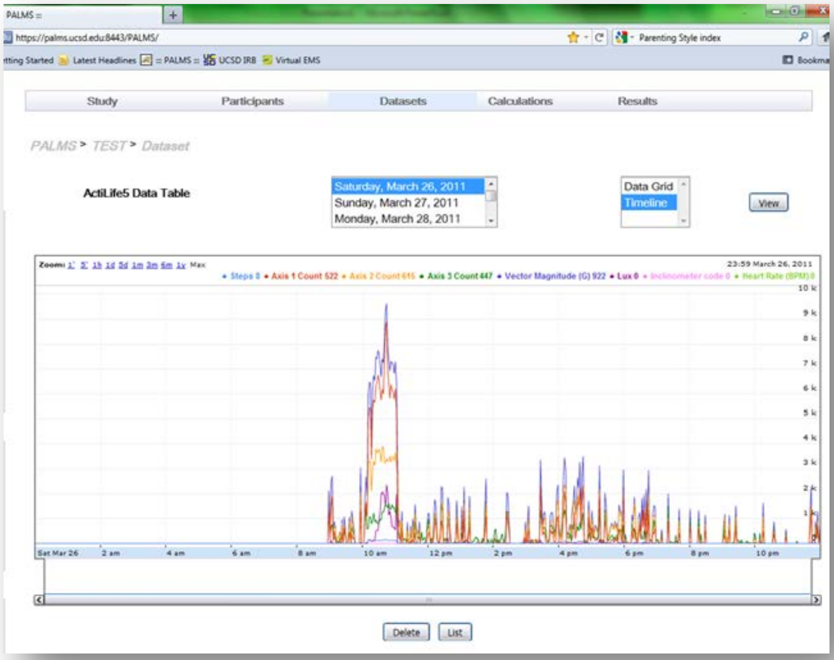

GPS and activity data can also be viewed in tabular format using the data grid button. 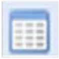

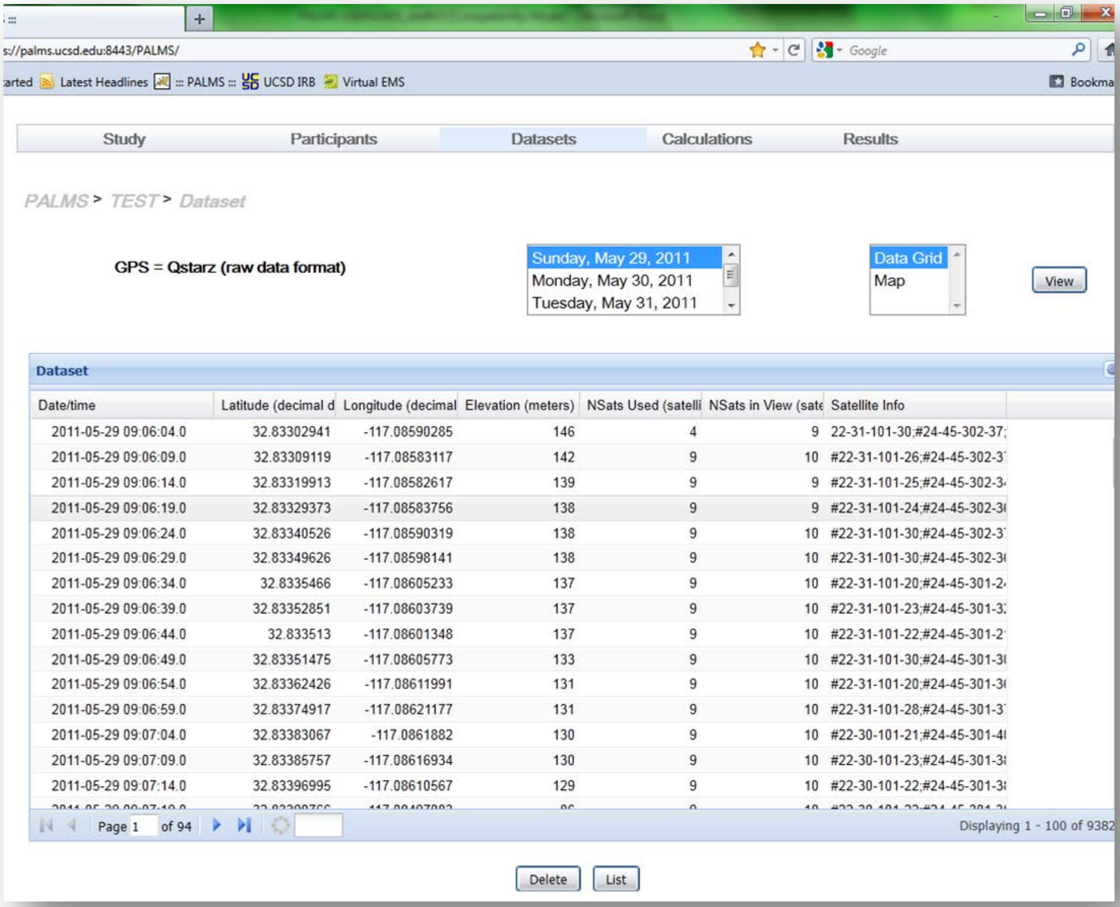

8.4 Calculations

In PALMS, calculations are the functions used to define the parameters for filtering and merging data and for summarizing results sets. In PALMS there is one main calculation called GPS Activity HR – Process and Merge (R2). This calculation:

- filters GPS and activity data
- detects trips, locations, and bouts of activity
- classifies transportation modes and activity intensity
- calculates energy expenditure; and,
- merges the GPS and activity data.

The other calculations in PALMS use the results set from the main calculation to create summary variables.

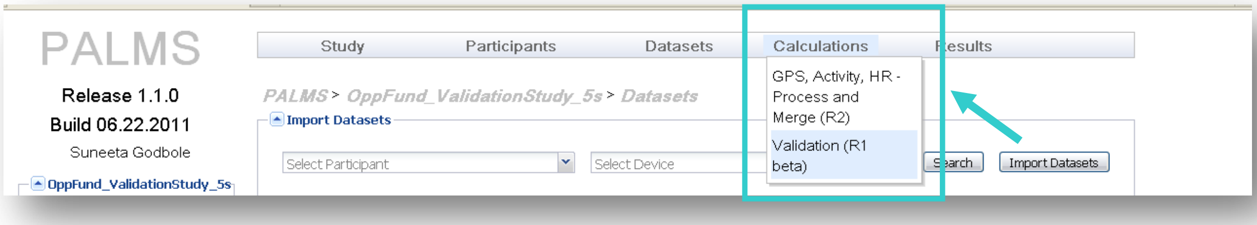

To run a calculation, select Calculations from the PALMS main menu and choose the calculation you would like to run from the list. In the following pages we'll go over the parameters for each unique calculation:

1. GPS, Activity, HR – Process and Merge (R2) [main calculation]
2. Daily Summary v1.0

8.4.1 Saving Parameter Protocols

Parameter settings can be saved as a protocol for PALMS. PALMS is set up with default parameters, which are saved as a default protocol. When you make changes to the parameters, the protocol can be saved under a new name.

To save a protocol, click on the Edit Protocol link and click Save As to specify a protocol name. Modify the parameters as desired, and click Save. The saved protocol can be retrieved at a later session by selecting the protocol from the top Select a Protocol dropdown menu.

8.4.2 GPS, Activity, HR – Process and Merge (R2)

PALMS currently processes one data stream at a time (i.e., GPS data, accelerometer data, etc.) and merges the data streams after processing. Consequently, when a data point is removed by an invalid value filter, only the data associated with that device is removed.

Users can define parameters for GPS, accelerometers, heart rate, energy expenditure and merge controls. Click on the down arrow on the right side of the function name to open the parameters and edit the protocol for each section.

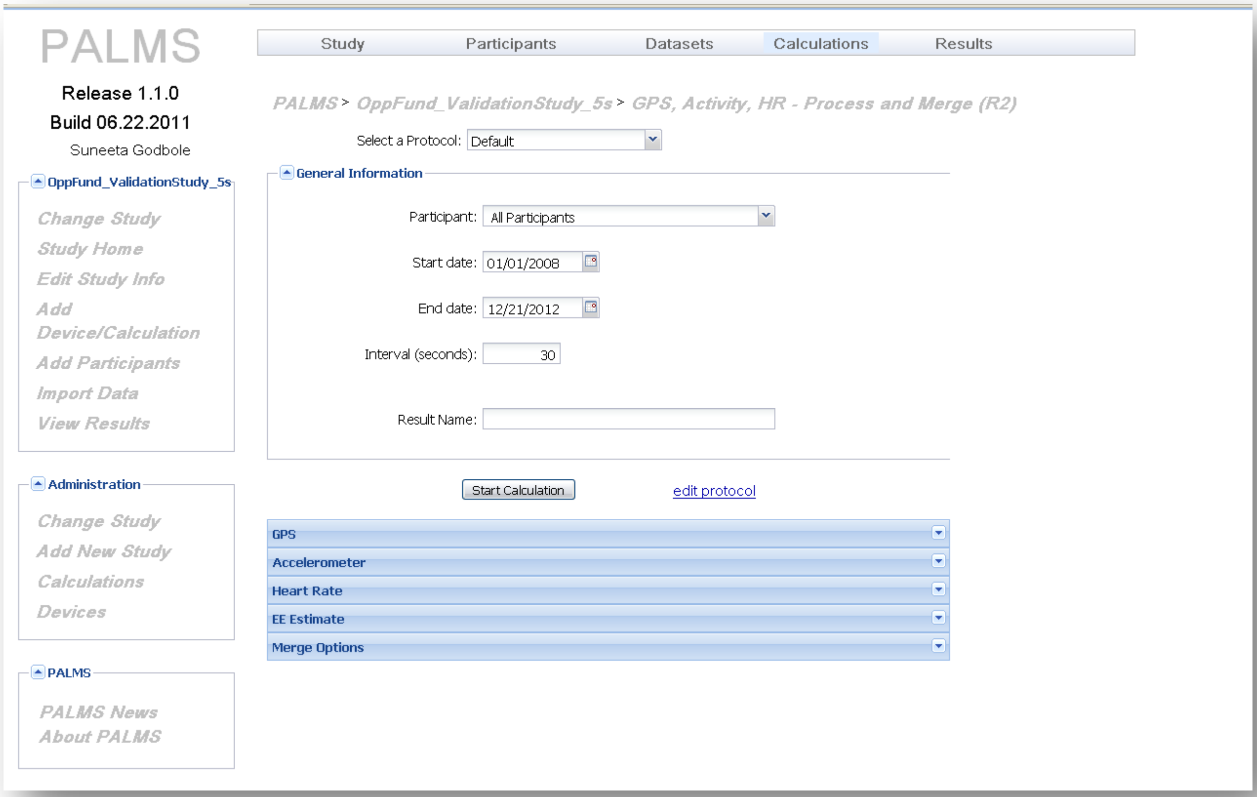

8.4.2.1 General Information Parameters

Participant – Users can select one participant or all participants for a calculation.
Start date *(mm/dd/yyyy)* – Restricts the time period for the results set (first day).
End date *(mm/dd/yyyy)* – Restricts the time period for the results set (end day).
Interval – (seconds) This sets the results interval. Users can set the results interval to any time epoch greater than or equal to the collection epoch of their devices. When the results interval is greater than the data collection interval for activity data, activity counts are aggregated to the results interval epoch by summing the values within the interval. When the results interval is greater than the data collection interval for GPS data, GPS points are selected based on the time stamp (see Merge Option p. 36).
Result name – Users can specify any result name to find their results set in the results table.

8.4.2.2 GPS Parameters

The GPS parameters are as follows: (1) filtering invalid values, (2) indoor/outdoor detection, (3) computing averages, (4) location detection, (5) trip detection, and, (6) speed cutoff values.

Before applying the GPS parameters, PALMS completes a preprocessing step in which it calculates the distance, speed and elevation change for consecutive records in the data file, using the latitude and longitude coordinates for each point. During the preprocessing step, PALMS also extracts satellite information from Qstarz devices configured to collect this information. Finally, PALMS labels fixes as

"first", "last" and "lone" fixes using the Loss of Signal parameter (see Max Loss of Signal Allowed p. 28).

8.4.2.2.1 Filter Invalid Values

GPS receivers are constantly computing their location (or fix). Occasionally, they return a spurious data point due to a poor satellite signal or interference from buildings. PALMS attempts to detect these implausible data points and will filter (remove) them. The parameters in this section control how PALMS determines that a fix is invalid.

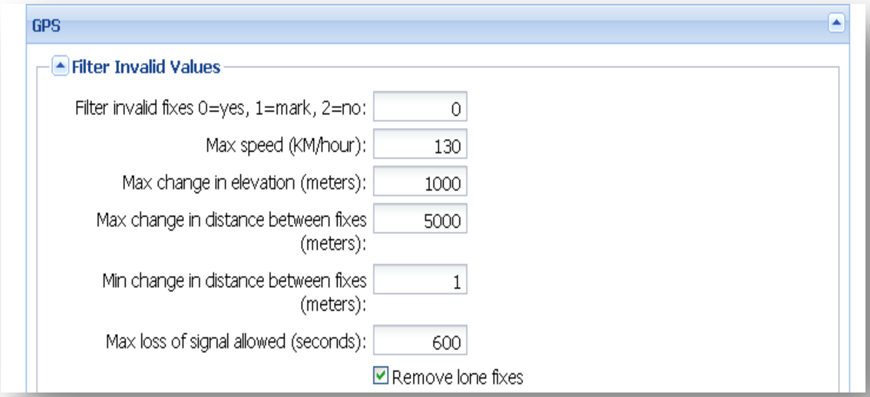

Filter invalid fixes – This parameter allows the user to decide if they want to filter out values that fall outside of the filter criteria (next 4 parameters) from the calculation and subsequent results sets. The options are:

0 - Removes invalid GPS fixes before detecting trips and location and classifying transportation modes. This option removes GPS fixes in the results set, but these fixes are retained in the raw datasets.

1 - Marks the invalid GPS fixes before detecting trips and locations and classifying transportation modes.

2 - No filtering – all fixes are used in the calculation.

PALMS calculates speed, distance change and elevation change between consecutive records in the data file regardless of the difference in time between those points. This effects the maximum change in elevation, maximum change in distance and minimum change in distance parameters.

Max speed (km/h) – Data points with speed values greater than the value selected in this parameter are determined to be invalid and are removed from the results set. (They remain in the participant dataset.) The instantaneous speed is recalculated between the next point and the previous point.

When the max speed criteria is set at 130 km/h:

Time	Speed
t_0	26
t_1	135
t_2	130

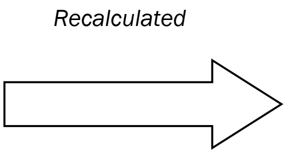

Time	Speed
t_0	26
t_2	25

The data associated with t_1 is removed from the results set and the speed from t_0 to t_2 is recalculated for t_2.

Max change in elevation (m) – Data points with an elevation change value greater than selected in this parameter are removed.

Min change in distance between fixes (m) – Data points with a change in distance value less than selected in this parameter are filtered out of the calculation and results set.

The minimum distance parameter has two functions in PALMS: it removes points that provide no significant new information; and, it removes invalid points caused by GPS "jitter." The accuracy of GPS devices varies across brands and leads to slight variations (jitter) in the location fixes for non-moving individuals. These inaccurate GPS readings create noise in the data and provide no additional useful information for detecting trips or locations. Therefore, when there is a change in distance between two consecutive fixes that is less than the minimum distance parameter, the latter fix is removed from the results set. (See illustration below.) New distance, speed, and elevation change values are then computed.

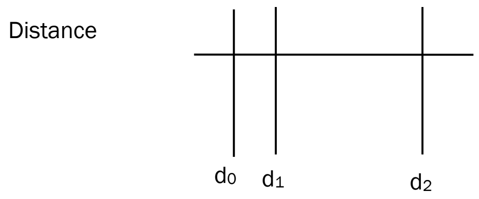

When the distance between d_0 and d_1 is less than the minimum distance parameter, d_1 will be removed and the distance between d_0 and d_2 will be calculated.

Noise also appears as the sudden movement away from a location, followed by a return to the location. To clean this type of noise, the minimum distance parameter calculates the distance between three consecutive fixes. It compares the distance between the first and second fix to the distance between the first and third fix. If the distance between the first and second fix is greater than the minimum distance parameter, and the distance between the first and third fix is less than the minimum distance parameter, the second fix, and all other data associated with the point, are removed from the calculation and results set as illustrated below. Then the speed, distance, and elevation change between the first and third fix are recalculated.

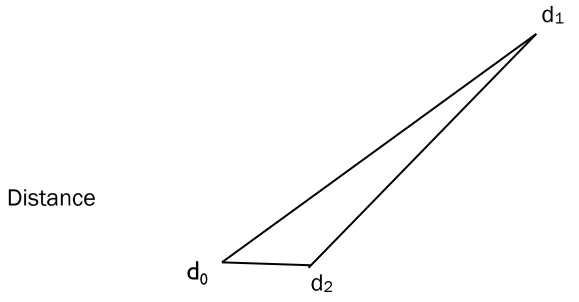

If the distance between d_0 and d_1 is greater than the minimum distance parameter, but the distance between d_0 and d_2 is less than the minimum distance parameter, then d_1 is filtered out and speed, distance and elevation change are recalculated.

Max loss of signal – The longest time period before a loss of signal is declared. A loss of signal (LOS) is a period of time during which the GPS device could not fix its location; usually caused by being in a building or tunnel. The loss of signal parameter is used in the preprocessing step when PALMS marks first, last, and lone fixes. First and last fix labels are used to stop these points from being removed from the dataset. First and last records in a data file are marked as first and last fixes. In addition, the last fix before a loss of signal and the first fix after a loss of signal are marked last fix and first fix respectively.

First/last fix illustration:

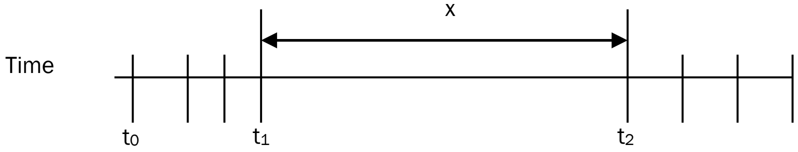

t_0 = initial GPS location fix – labeled by PALMS as “first fix”
x = gap between consecutive GPS location fixes at t_1 and t_2,
when x < LOS value (in seconds), PALMS does not label either point
when x > LOS value, PALMS marks t_1 as “last fix” and t_2 as “first fix”

Remove lone fix *(checkbox)* – This parameter removes fixes labeled as lone fixes from the results file. A lone fix is a location fix established between two loss-of-signal periods. Due to the loss of signal on either side of the lone fix, the validity of these fixes is questionable and users are given the option to remove the lone fixes in their calculations.

Lone fix illustration:

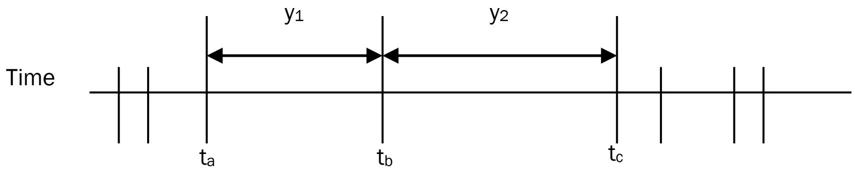

If y_1 > LOS < y_2 then t_b is categorized as a “lone fix”
If y_1 < LOS < y_2 then t_b will be categorized as a “last fix” and t_c will be a “first fix”
If y1 > LOS > y_2 then t_b will be categorized as a “first fix” and t_a will be a “last fix”

8.4.2.2.2 Indoor/Outdoor Detection (only for use with Qstarz devices if you configure them to collect satellite information)

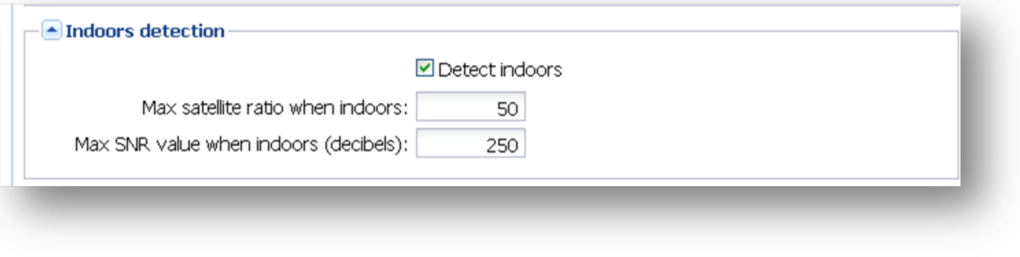

During the device configuration process it is necessary to specify the collection of these data (see Appendix B). The indoor/outdoor designation is indicated in the output file under the fix type.

Detect indoors – This checkbox sets PALMS to classify fixes as indoor or outdoor.

Max satellite ratio when indoors – Sets the maximum ratio of satellites in-view to satellites used for an indoor classification. When the satellite ratio is greater than this parameter, the fix is classified as outdoor.

Max SNR value when indoors (decibels) – Sets the maximum signal to noise ratio value for an indoor classification. When the satellite SNR value is greater than this parameter, the fix is classified as outdoor. This is the primary parameter to classify indoor/outdoor. The satellite ratio parameter (above) is used when SNR values are not collected.

8.4.2.2.3 Compute Averages (optional)

When plotting speed and elevation changes, it may be useful to use average values in place of the instantaneous values recorded by the GPS device. Since this step occurs *after* filtering and trip detection, selecting these options will output the average values in the results set.

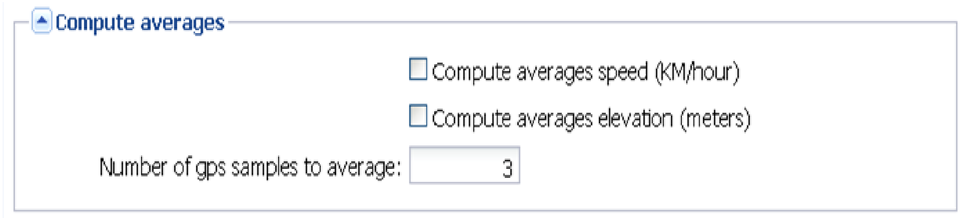

Compute average speed (km/h) – Computes the average speed using the number of samples specified and replaces the speed values in the output speed column with the average.

Compute average elevation (meters) – Computes the average elevation using the number of samples specified and replaces the elevation values in the output elevation column with the average.

Number of GPS samples to average – This parameter allows the user to select the number of data points to use in computing the moving average for speed and elevation changes.

8.4.2.2.4 Location Detection

To classify location, PALMS calculates the total number of stationary points around start points, end points, first fixes, last fixes and (optionally) pause points within the user-defined cluster radius. Stationary, start, end and pause points are categorized in the trip detection calculation (see Section 9.4.2.2.5). Once these potential location clusters are defined, the distance between the clusters is calculated and points that are within the user-specified cluster radius are grouped. The locations are then determined based on the user-specified minimum amount of time at a location parameter. Detected locations are assigned a sequential number in the PALMS output, the first location detected is assigned 1, the second is assigned 2, and so on.

PALMS detects locations for each participant, which means that when All Participants are specified in a calculation protocol, participants that visit the same locations could have different location numbers assigned for these locations.

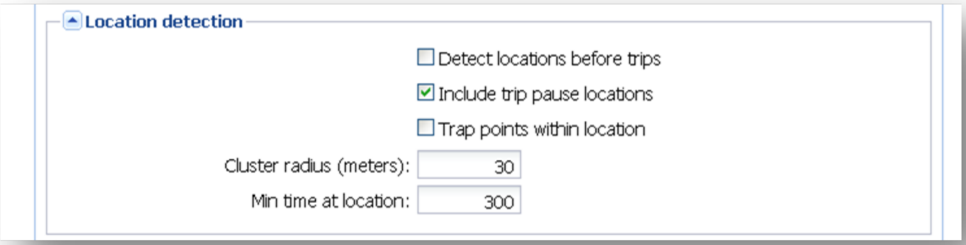

Detect location before trips – The default order is for PALMS to detect trips before detecting *locations*. Selecting this option reverses the order and has PALMS detect locations before *trips*.

Note*: We do not recommend using this option as it is still under development.*

Include trip pause locations – Checking this option allows pause points within trips to be locations.

Trap points within location – When checked, the latitude, longitude and elevation of points that are assigned to a location, but not part of a trip, are replace by the coordinates of the center of the location cluster.

Cluster radius (meters) – This is the user-defined radius of cluster of points for the initial pass to begin the location detection process. For example, in the default protocol, points within a 30 meter radius are likely to be one location and the jitter is just due to interference: the cluster location detection collapses these points.

Min time at location (seconds) – User-defined validation criteria for the classification of points as locations. A cluster of points can only be classified as a location if the total time (consecutive) at the location is greater than this parameter.

8.4.2.2.5 Trip Detection

PALMS uses a state-based method for classifying points as stationary, moving, and paused states. This method requires that specific criteria are met before changing from one state to another. The initial state is stationary, as shown below in the trip detection state diagram. Detected trips are also assigned numbers in a sequential order in the PALMS output. The first trip for a participant is labeled 1, and so on.

Trip Detection State Diagram

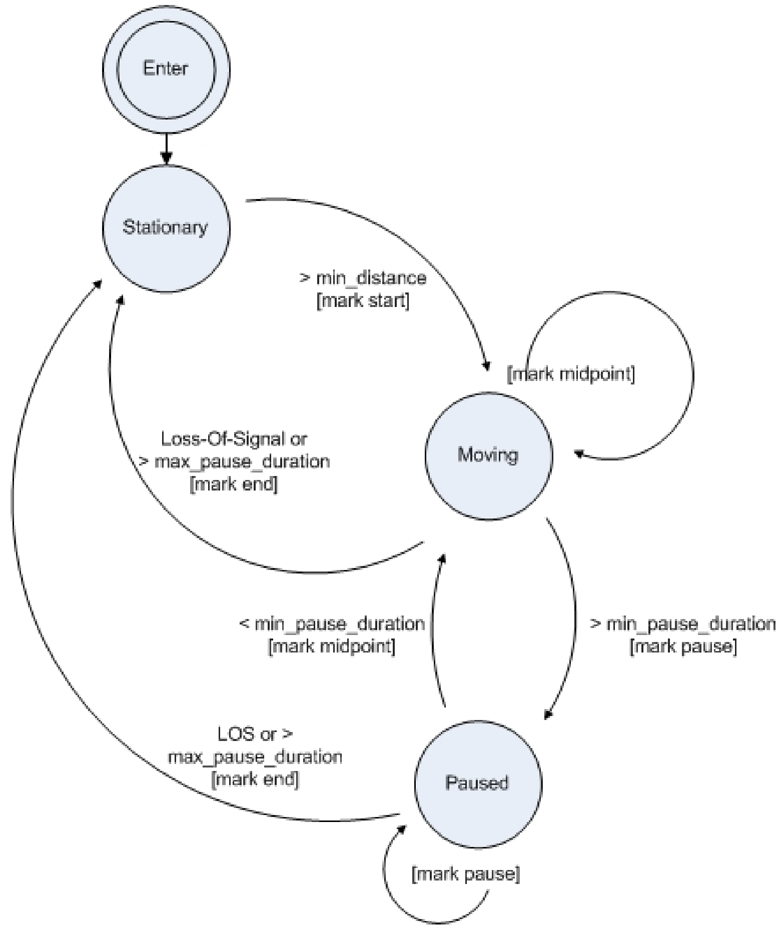

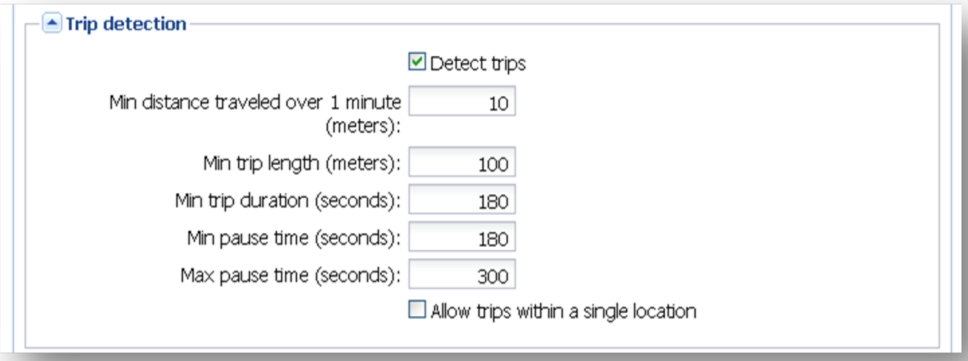

Detect trips – A check in this box instructs PALMS to detect trips. Trip detection is required for mode detection.

Min distance travelled over 1 minute (meters) – This minimum value determines the start point of a trip. PALMS sums the distance traveled in 1 minute for each fix by adding consecutive distances. When PALMS locates a fix where the distance travelled in 1 minute is greater than the parameter value, the point is marked as a trip start point.

Min trip length (meters) – The minimum length of a trip to be considered valid.
Min trip duration (seconds) – The minimum duration of a trip to be considered valid.
Min pause time (seconds) – The minimum duration of a pause to be detected.
Max pause time (seconds) – The maximum duration of a pause. When the duration is exceeded, PALMS will assign the trip an end point.
Allow trips within a single location – When checked, this option allows PALMS to detect trips within a location. As a default, when PALMS detects that a trip occurred within one location, it assumes the "trip" was actually GPS jitter. It will be discarded and the fixes will be classified as stationary points.

Tip: *Users should change the trip parameters based on the population under study. The speed of travel for active trips can vary between populations of older adults and children.*

8.4.2.2.6 Speed Cutoff Values

The maximum and the average speeds of the trip are calculated and used to define the transportation mode. The values specified in the parameters are minimum values for each activity.

Note: *There are currently only three classifications: Vehicle, Bicycle and Pedestrian. Running and Jogging are not yet implemented.*

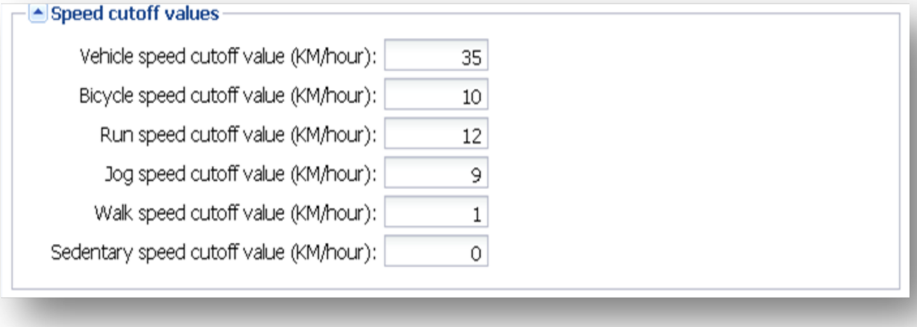

Vehicle speed cutoff value (km/h) – When the maximum speed of a trip is greater than or equal to this value, the trip is classified as a vehicle trip.
Bicycle speed cutoff value (km/h) – When the average speed of a trip is greater than or equal to this value, the trip is classified as a bicycle trip.
Walk speed cutoff value (km/h) - When the average speed of a trip is greater than or equal to this value, the trip is classified as a pedestrian trip.
Sedentary speed cutoff value (km/h) – This value is not used and can be ignored.

Tip: *Walking speed differs for different populations. Consider your population when choosing a speed.*

8.4.2.3 Accelerometer Parameters

The accelerometer parameters are used to clean and score the accelerometer data. These parameters are: Invalid Value Filters, Activity Bout Detection, Sedentary Bout Detection, and Activity Intensity Classification Cutoff Values.

8.4.2.3.1 Invalid Value Filter

These parameters distinguish between sedentary and non-wear periods.

***Note**: Missing values (when there are GPS fixes and no accelerometer data) are labeled with a –1 in the output file.*

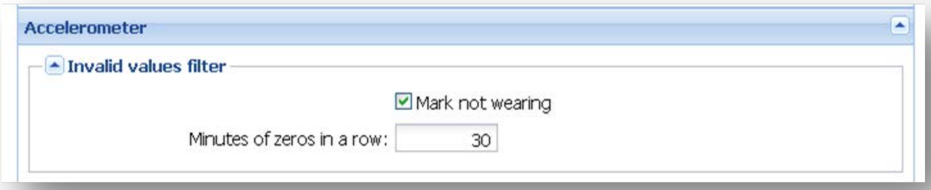

Mark not wearing – This option marks the non-wearing periods by changing the 0 count to a –2 when the data are within a period of time that meets the non-wear criteria.

Minutes of zeros in a row – This parameter allows the user to specify the number of minutes of consecutive 0 counts to be reclassified as a non-wear period.

***Warning**: there is no tolerance threshold in this parameter, and data cannot be screened.*

8.4.2.3.2 Activity Bout Detection

A bout is a period of activity during which the accelerometer counts are consistently between a lower and upper limit for a defined number of minutes. A tolerance is also defined, allowing for counts outside the limits, such as brief pauses that may occur when a runner stops for a red light. Bouts detected in PALMS are assigned sequential numbers based on their occurrence for each participant.

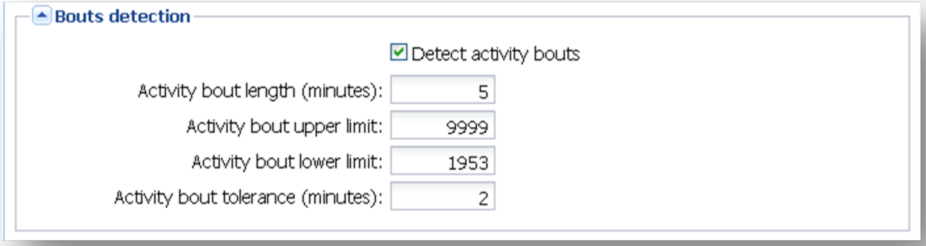

Detect activity bouts – Checking this box turns on the bout detection calculation.
Activity bout length (minutes) – The minimum duration of the activity bout.
Activity bout upper limit – The upper count limit for a bout (counts per minute).

Activity bout lower limit – The lower count threshold for a bout (counts per minute).

Activity bout tolerance (minutes) – The time that activity counts can be outside the specified range and still be considered part of the bout.

8.4.2.3.3 Sedentary Bout Detection

A sedentary bout is a period of inactivity during which the accelerometer is between zero and an upper limit for a defined number of minutes. A tolerance is also defined for brief or periodic periods of activity, enabling sedentary counts in excess of the defined upper limit. Sedentary bouts are assigned sequential numbers based on their occurrence in the PALMS output for each participant.

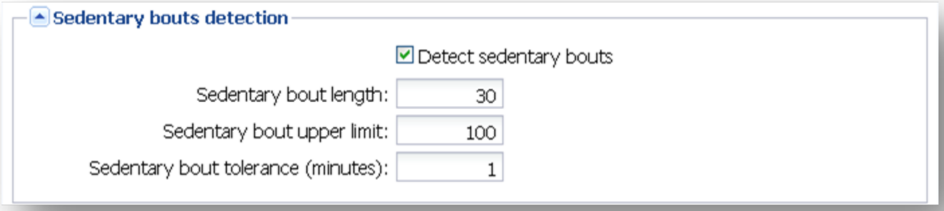

Detect sedentary bouts – This checkbox turns on the sedentary bout detection calculation.

Sedentary bout length – The minimum duration of the sedentary period to be classified as a bout.

Sedentary bout upper limit – The upper count limit for a sedentary (in counts per minute).

Sedentary bout tolerance (minutes) – The period of time that activity counts can go higher than the specified upper limit and still be considered part of the bout.

8.4.2.3.4 Activity Intensity Classification Cutoff Values

These parameters define the cutoff values for accelerometer readings used to classify activity intensity. These values are defined on a per-minute basis, and PALMS divides the specified parameter value for lower interval length.

Note: *Cutoffs have only been validated at a 60-second epoch. Therefore, users should compare results with the data aggregated to 60 seconds, and then the divided value for lower epochs.*

A light cutoff value of 400 will be divided evenly to 100 for a 15-second result interval length. Then each accelerometer value in the result interval will be compared to 100 to determine if it is light activity.

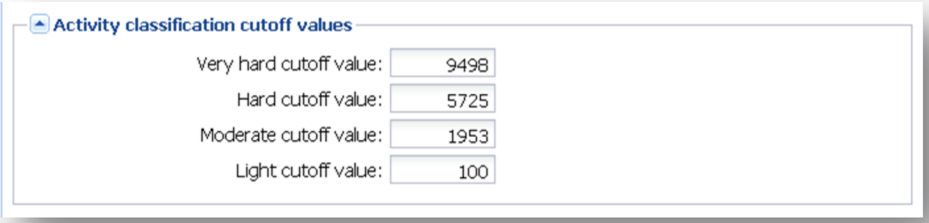

Very hard cutoff value – Accelerometer values greater than this parameter are classified as very hard intensity (per minute).

Hard cutoff value – Accelerometer values greater than this parameter are classified as hard intensity (per minute).

Moderate cutoff value – Accelerometer values greater than this parameter are classified as moderate intensity (per minute).

Light cutoff value – Accelerometer values greater than this parameter are classified as light intensity (per minute).

The activity intensity classification output is an ordered categorical variable with values as described below:

Values	Description
-2	Non-wear time
-1	Missing data
0	Sedentary activity intensity classification
1	Light activity intensity classification
2	Moderate activity intensity classification
3	Hard activity intensity classification
4	Very hard activity intensity classification

Tip: *Activity cutoffs vary in children. If you create activity specific studies for each activity cut off you can run all participants in one calculation.*

8.4.2.4 Heart Rate Parameters

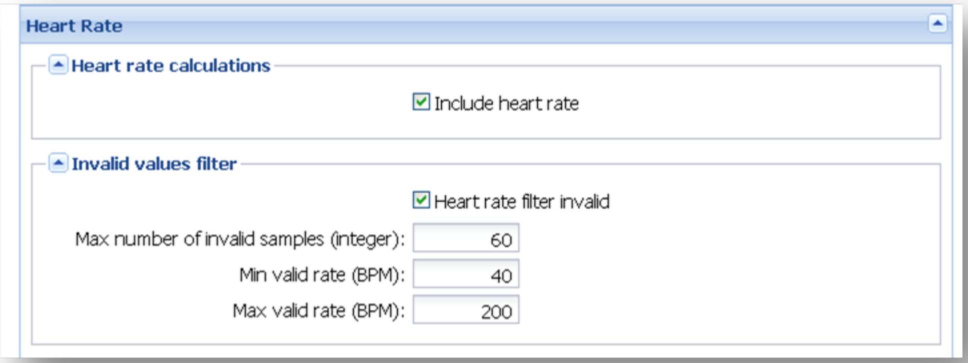

8.4.2.4.1 Heart Rate Calculations

Include heart rate – When checked, if heart rate data is available it will be processed. Otherwise, heart rate variables in the results set will be set to –1.

8.4.2.4.2 Invalid Values Filter

Heart rate monitors require firm contact with the participant's skin. Loose contact generates noise, which results in readings of a very low or very high heart rate. The following parameters determine how PALMS handles such noise.

Values less than the Min Valid Rate or greater than the Max Valid Rate are considered invalid. If Moving Average is checked, invalid samples will be replaced by the moving average. Otherwise the invalid value will be replaced by the previous valid value.

The field Max Number of Invalid Samples sets the maximum number of consecutive invalid readings that will be replaced. When the maximum number is reached, invalid values are replaced by –2 to indicate bad data. (–1 indicates missing data).

Heart rate filter invalid – When checked, PALMS will filter out invalid heart rate values.
Max number of invalid samples (integer) – Maximum number of consecutive invalid readings allowed.
Min valid rate (BPM) – Minimum value for a valid heart rate reading.
Max valid rate (BPM) – Maximum value for a valid heart rate reading.

8.4.2.4.3 Moving Average

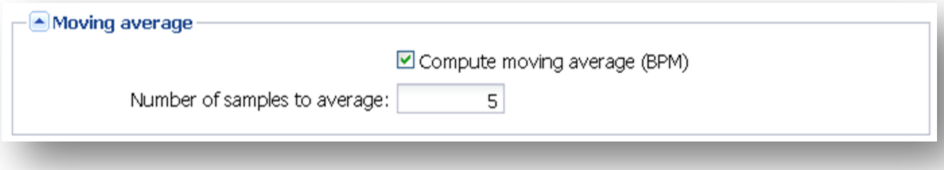

Compute moving average (BPM) – When checked, PALMS computes a moving average for heart rate.
Number of samples to average – This parameter defines the number of consecutive samples to average.

8.4.2.4.4 Max Heart Rate (HR) Formula

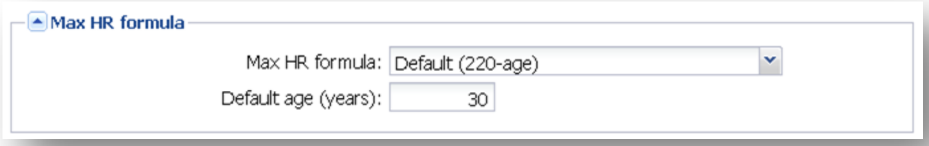

Max HR formula – Dropdown box to select the formula used to calculate the maximum heart rate. In all cases, the maximum heart rate is calculated as in integer value.

Formula	Description
Default (220 – age)	Subtracts age from 220
Londeree	206.3 – (0.0771 * age)

Miller	217 – (0.85 * age)
Kavonen	206.9 – (0.67 * age)

Default age (years) – All max HR formulas are based on the participant's age. If the participant's age is not available (i.e., was not entered on the Participant page), the value entered by the user will serve as an estimate of the participant's age.

8.4.2.4.5 HR Bout Detection

Bouts based on heart rate are assigned sequential values in the PALMS output for each participant.

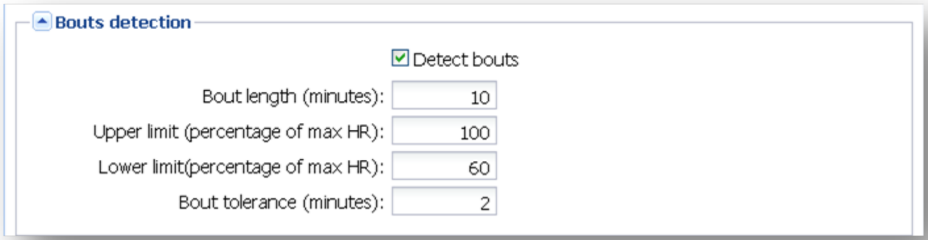

Detect bouts – When checked, PALMS will detect heart rate bouts.
Bout length (minutes) – The minimum duration of a HR bout.
Upper limit (percentage of max HR) – The highest percentage of the maximum heart rate that can be part of a heart rate bout.
Lower limit (percentage of max HR) – The lowest percentage of the maximum heart rate that can be part of a heart rate bout.
Bout tolerance (minutes) – The amount of time that heart rate can be outside the upper and lower limits and still be included in the heart rate-based activity bout

8.4.2.5 Energy Expenditure (EE) Estimate Parameters

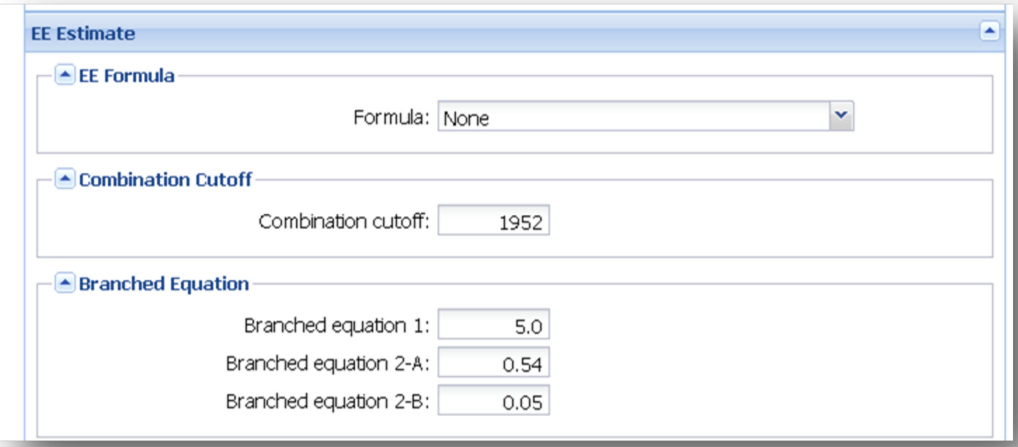

Energy expenditure can be estimated using several different formulas, as described below:

EE formulas

Formula	Description
None	Do not estimate EE. EE is set to –1.
Device Default	Use the EE value estimated by the device's software and upload to PALMS.
Freedson	For ActiGraph devices only – use Freedson formula based on counts and participant's weight.
Work-Energy	For ActiGraph devices only – use Work-Energy Theorem (WET) formula based on counts and participant's weight.
Combination	For ActiGraph devices only – use a combination of the Freedson and Work-Energy Theorem formula based on the combination cutoff value (see below).

Combination cutoff - When the Combination formula is selected, for counts below this value PALMS will use WET. For counts greater than or equal to this value, PALMS will use Freedson. Note: The participant's weight must have been entered.

Branched equation – Not yet implemented.

8.4.2.6 Merge Options

To merge the GPS and activity time-stamped data streams, PALMS creates a new timeline based on the start and end times of either the GPS or activity data streams. PALMS will create a timeline according to the user-specified device interval length (epoch) under the General Information parameters (see p. 23). PALMS will align fixes from the GPS stream to the timeline by comparing timestamps and merging data when the timestamp is congruent with the timeline. PALMS will aggregate accelerometer data to the results timeline as illustrated below:

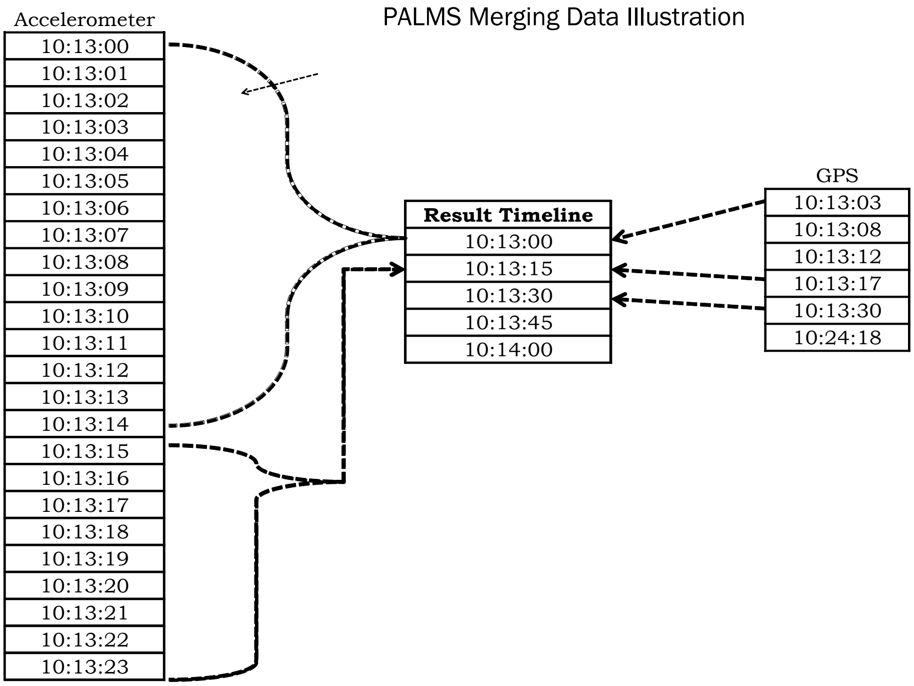

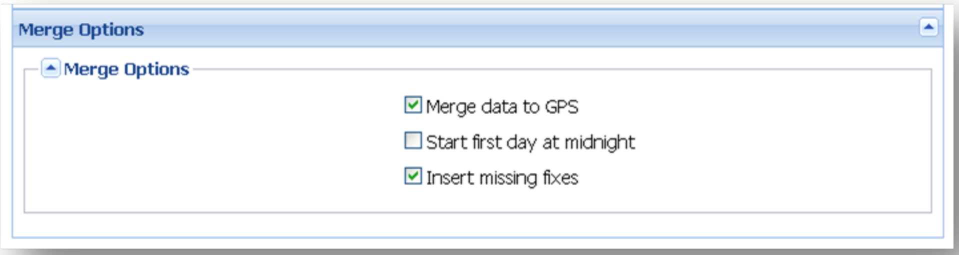

Merge data to GPS – This parameter instructs PALMS to use the start and end time from the *GPS data stream* to start and end the results timeline. When unchecked, PALMS will use the start and end time from the *activity data stream* to create the start and end times for the results timeline.

Start first day at midnight – This parameter instructs PALMS to start the results timeline at midnight on the first day of either the GPS or activity data stream.

Insert missing fixes – The parameter has PALMS insert fixes when GPS or accelerometer data is missing. The last valid fix will be inserted for the missing fix, while a –1 will be added for missing data in the activity variable.

***Note**: Any number of options can be checked.*

8.4.3 Daily Summary v. 1.0

The Daily Summary compiles the one-row-per-epoch output of the PALMS calculation (described above) by participant, day and event. An event is defined as a change in the participant's location (a trip), physical activity level (bout) or sedentary behavior.

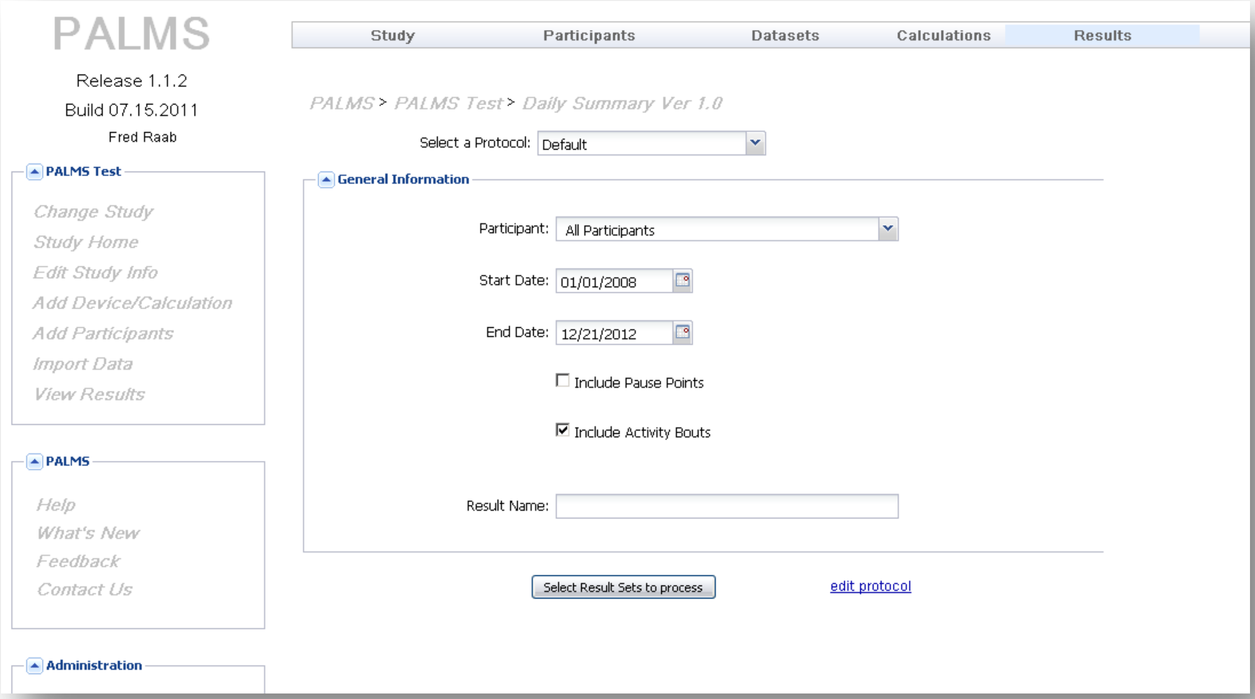

The Daily Summary parameters are:

Participant –Users can select a single participant, or all participants for a calculation.
Start date *(mm/dd/yyyy)*– Restricts the time period for the results set (first day)
End date *(mm/dd/yyyy)* – Restricts the time period for the results set (end day)
Result name – Name of the results set produced by the calculation.

***Note**: It is recommended that the name starts with "DS – " to distinguish it from other results sets.*

Include pause points – When checked, pauses in a trip will be included in the output. If unchecked, only trip start and end points will be included.
Include activity bouts – When checked, the start and end of each activity bout will be included in the output.

When the parameters are selected, click the Select Results Sets to Process button. A list of results sets (previously generated by the PALMS calculation) will be displayed. Click on the name of a results set to summarize it, and then click the Start Calculation button.

***Important**: The results of the Daily Summary calculation can be exported as CSV files, but not KML files (see Section 8.5).*

8.5 Viewing/Exporting Results

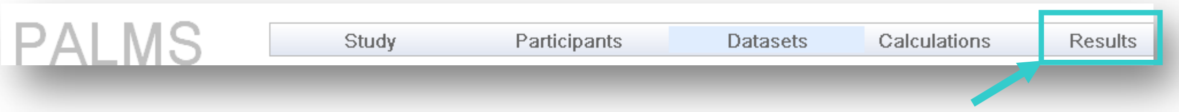

Once you have set a calculation to run, you may check on the progress of the calculation and export the results set by clicking on the Results menu from the main menu bar or the View Results in the left panel.

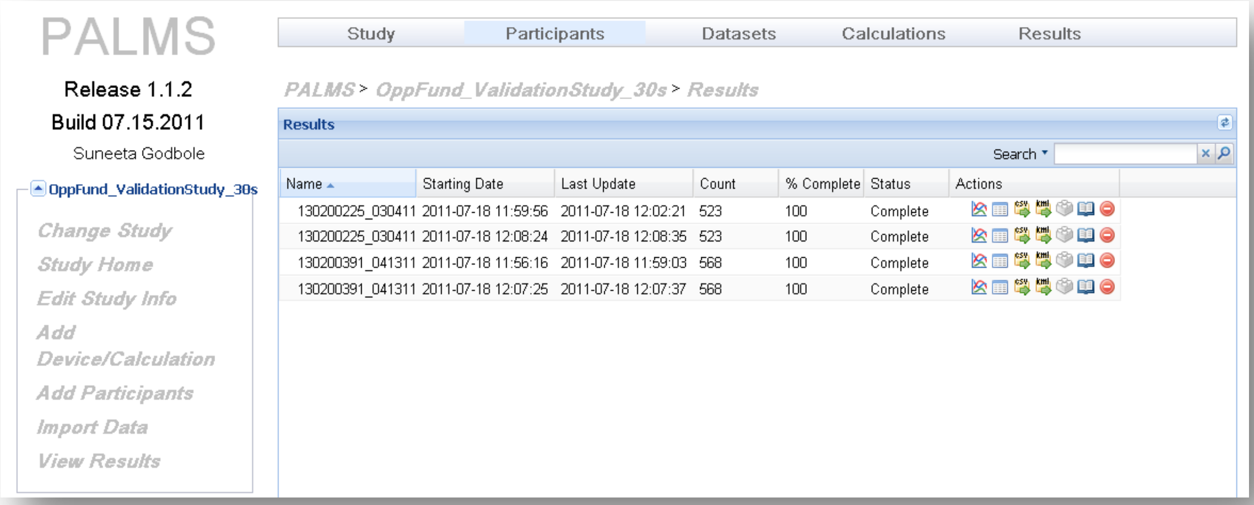

The Results table includes the following information:

Name – Name you assigned to the results set when calculation was set up.
Start date – Date and time the calculation was started.
Last update – Date and time the calculation last updated the results set.
Count – Number of rows written to the results set.
% complete – Estimate of the percent complete for the calculation.
Status –

Executing – PALMS is currently running the calculation. To see the progress, click on the refresh button in the top right corner of the Results table.

Complete – PALMS has completed the calculation.

Failed – PALMS failed to execute the calculation. Details are contained in the calculation log file (see below). Please contact the PALMS administrator for troubleshooting.

Actions –

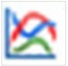 Displays the results set on a map.

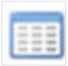 Displays the results set as a dataset.

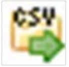
Exports the results set as a CSV file.

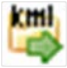
Exports the results set as a KML file.

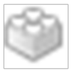
Displays the protocol used to create the results set.

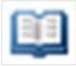
Displays the log file with detailed information on the calculations.

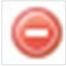
Deletes the results set.

Note*: The PALMS project team does not recommend using the dataset and map display functions within PALMS for large results sets (>50,000 rows) due to the length of time it takes to process in the PALMS browser. It is quicker to export results sets as CSV or KML files (see below).*

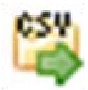
CSV stands for comma separated values files, which can be read by multiple programs including Microsoft Excel, SPSS, and GIS. Clicking on the CSV export button will open a pop up window (see below). Users select the variables and the order of the variables for export in a CSV file format by moving them from the left to right panel with the arrow buttons.

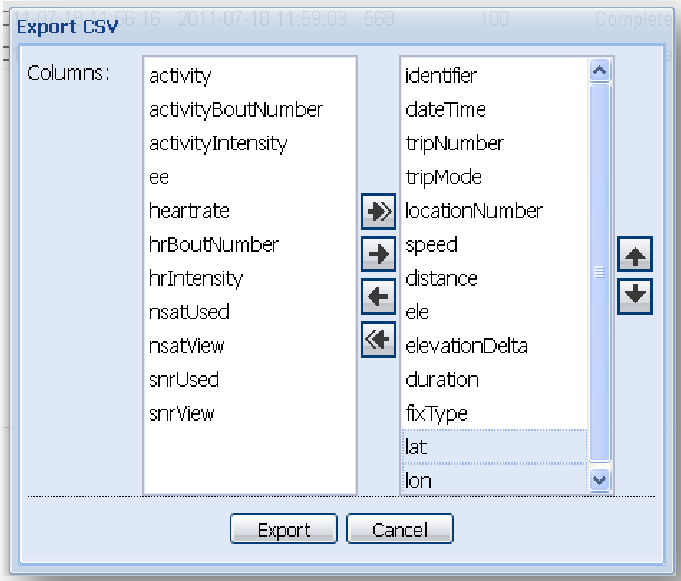

When opening a CSV exported file in Excel you must change the format on the dateTime variable. Change the column format by highlighting the column, right-clicking to Format Cells and choose the Custom format type. Enter mm/dd/yyyy hh:mm:ss to display the dateTime

variable correctly. In SPSS, import this variable as a string and use syntax to create new Date and Time variables. To import to ArcGIS use the instructions in Appendix D (p. 60).

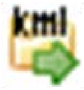

The KML file can be viewed in Google Earth to display the trips, locations and bouts of activity calculated by PALMS. Within Google Earth the results are stored in a set of folder which can be expanded using the + and – marks on the left of the folder name (See Appendix C for more details 56).

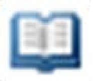

The log file can be useful in the determining how data are processed in PALMS. It can also be used to troubleshoot parameter settings. It does however contain much computer programming jargon, and so may not be informative for all users.

APPENDICES

A **Creating Data Table Files with ActiGraph Data Files**
How to convert an ActiGraph data file to a format that can be recognized by PALMS.

B. **Configuration and Download Procedures for Qstarz Devices**
How to configure and download devices with Qstarz's QTravel program and how to configure setting to record satellite information for indoor/outdoor detection in PALMS.

C. **Guidelines for Using Google Earth with PALMS**
Different options for visualizing the PALMS KML output in Google Earth.

D. **Importing PALMS CSV output into ArcGIS (by Jasper Schipperijn)**
How to import PALMS CSV outputs into ArcGIS.

E. **Examples of Participant Instructions (Wear and Charging)**
Samples of wear instructions given to participants wearing PALMS devices.

F. **Physical Activity Spatial Temporal Analysis (PASTA) Protocol**
The PASTA is a PALMS-based recall tool for identifying participant activities.

G. **Parameter Quick Reference Guide**
Brief explanations of the PALMS parameters

H. **PALMS CSV Output Dictionary**
Description of the variable formats in the PALMS output

Appendix A: Creating Data Table Files With ActiGraph Data Files

Before importing activity data to PALMS from the suite of ActiGraph devices, it must be pre-processed using the ActiGraph software. Use the ActiGraph software to create data table files from raw data files following the steps below.

If using ActiLife 4:

1. Open ActiLife program.
2. Click Analyze Data on the top menu bar.
3. Choose Show Data Table from the dropdown menu.
4. Browse to your Raw Accelerometer data file (.DAT) and click Open.
5. A new window will open – Save As the raw accelerometer file to a data table file (.CSV).
6. Click Save
7. The data table file (.CSV) will open in a new window

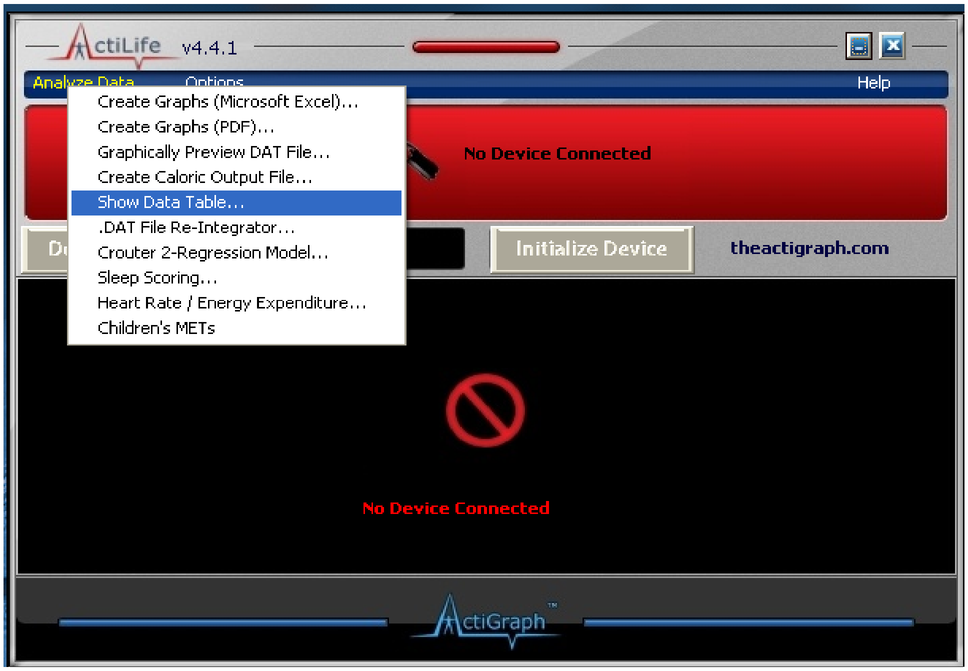

If using ActiLife 5:

1. Select Import/Export from the File menu.
2. Select .AGD -> Data Table (.CSV) from the Import/Export submenu.

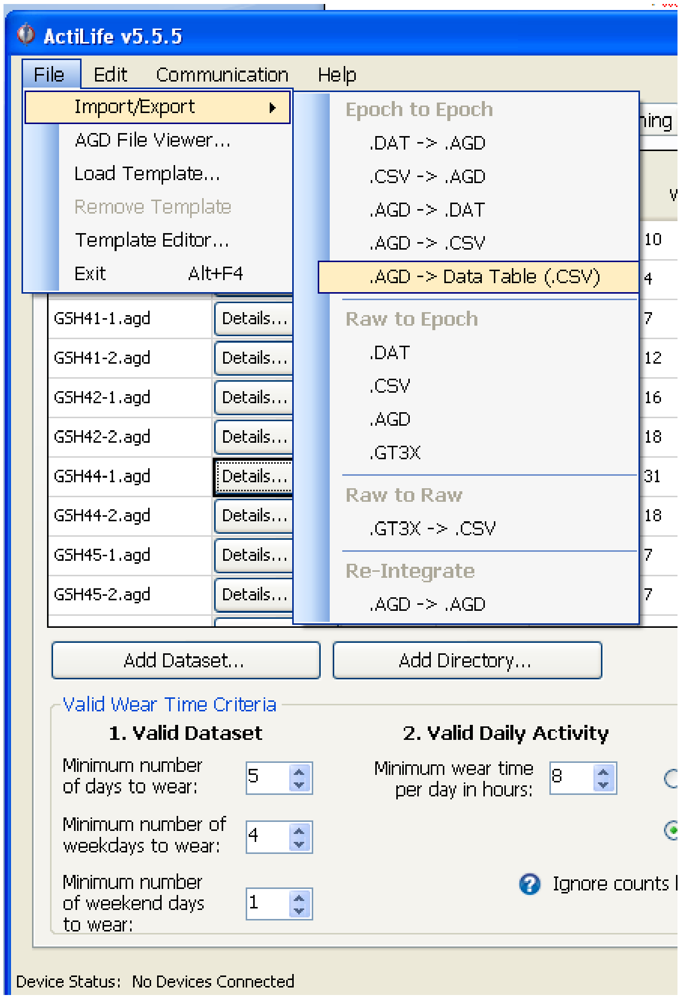

If using ActiLife 5 and the raw collection epoch (Hz) for a GT3X+ device:

1. Convert the .GT3X (raw acceleration file) to an .AGD file (count file).
    - Select Import/Export from the File menu.
    - Select .AGD in the Raw to Epoch section from the Import/Export submenu.

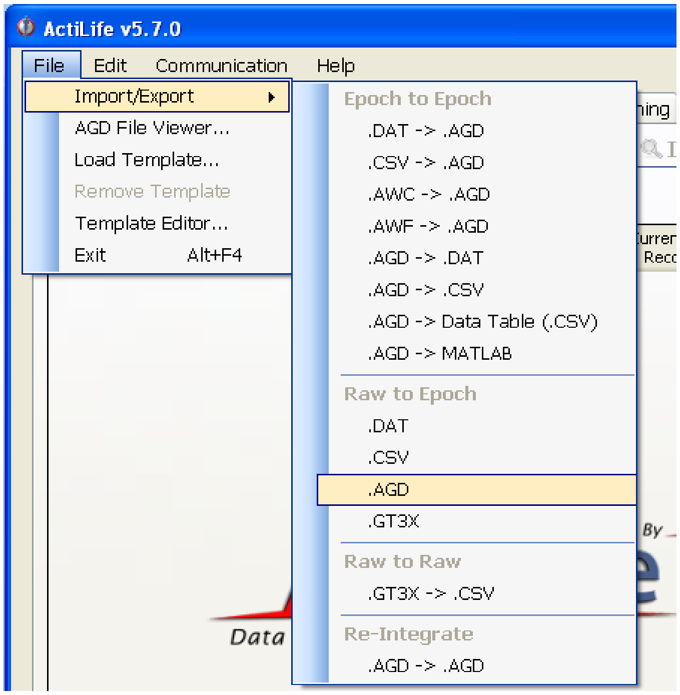

2. Convert the .AGD file to Data Table using the instructions on the previous page.

Appendix B: Configuration and Download Procedures for Qstarz Devices

B.1 Configuring GPS

1. Open Qstarz

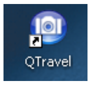

2. Select Config GPS from the File menu.

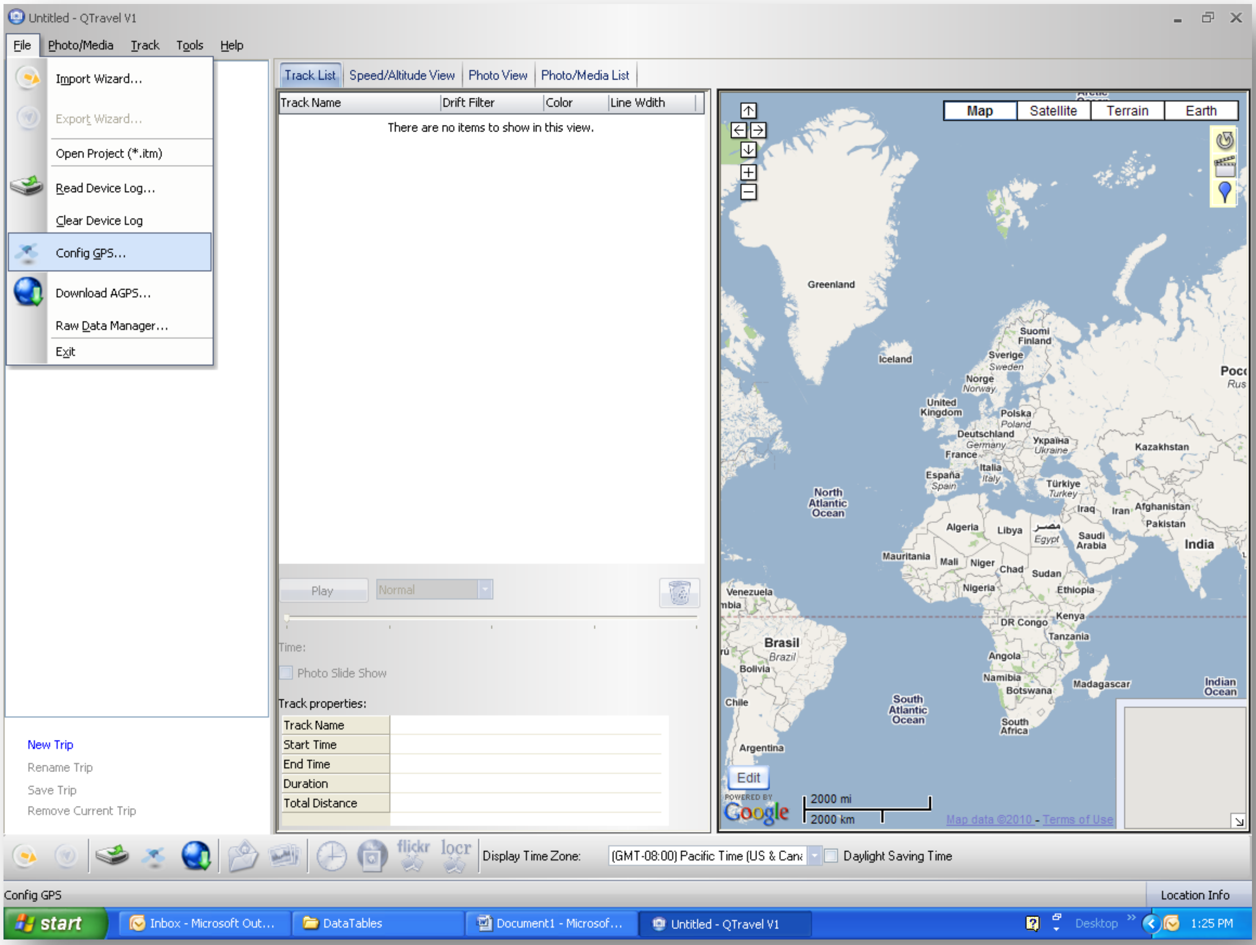

(a) If you cannot connect to the device and get the following error message:

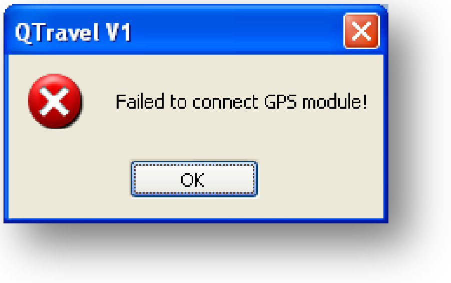

(b) Verify that the GPS is in **Log Mode.**

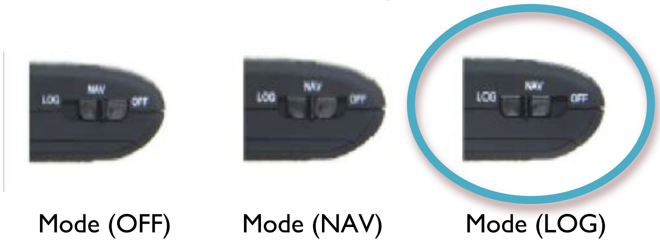

(c) Click **OK**
(d) When the Configure GPS Module window opens, click Connect.

3. Check the Data Log Memory section and verify that there is 0% memory used.

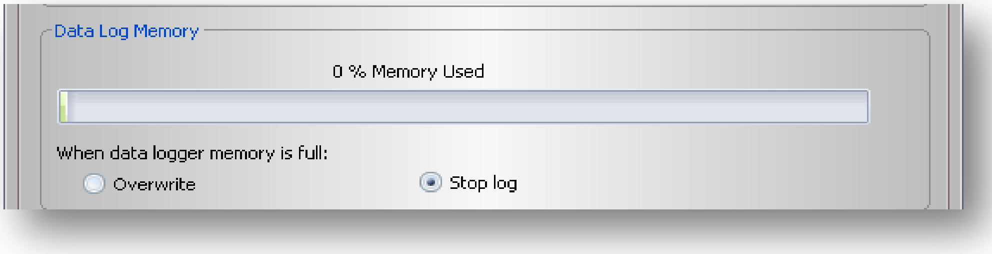

(a) If no data is present, skip to Step 4.
(b) If data is present on the device, click Cancel.
(c) Under the File menu, click Clear Device Log.
(d) Click Yes.
(e) Repeat Step 2.

4. Ensure that following settings are entered/checked:

(a) Connection Setting: Auto Scan GPS Module
(b) User Name: Participant Identifier and Time point information
For Example: PALMS_34_1
(c) GPS Log Setting: General
(d) Log Criteria: Set interval length for logging [Study Dependent].
(e) When the data logger memory is full: Stop Log
(f) Uncheck Turn on Buzzer box.

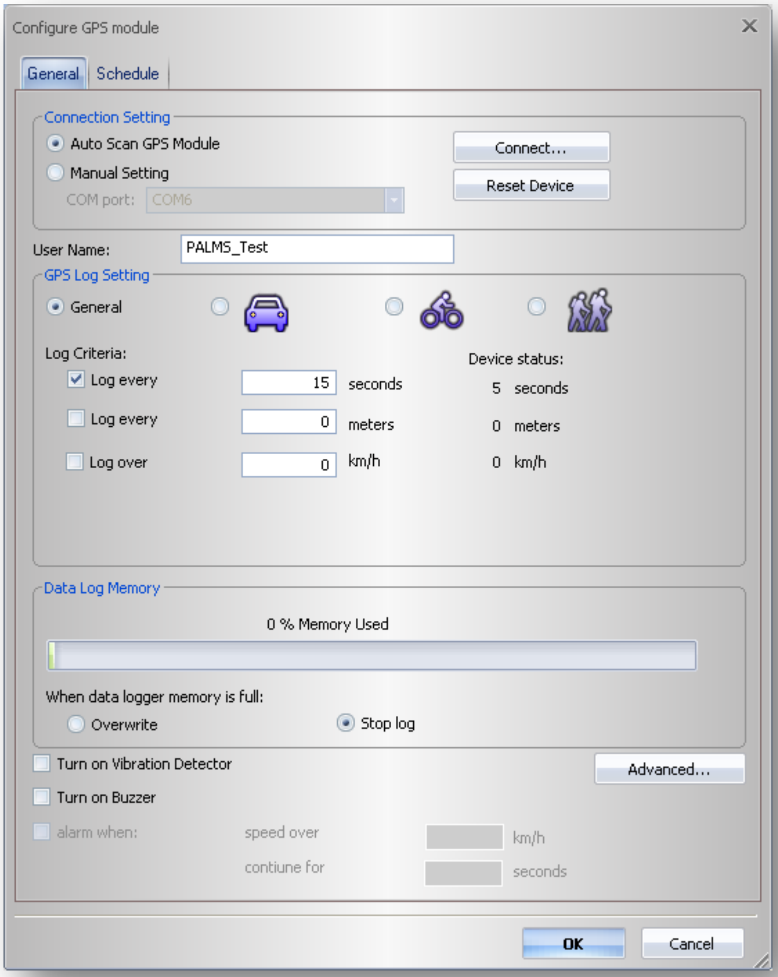

(g) Click OK.

***Note**: The "double" configuration protocol is NECESSARY to ensure that the device is configured correctly.*

5. Again, select Config GPS from the File menu.

   (a) Verify that the setting on the main Config window are as desired.
   (b) Click Advanced.

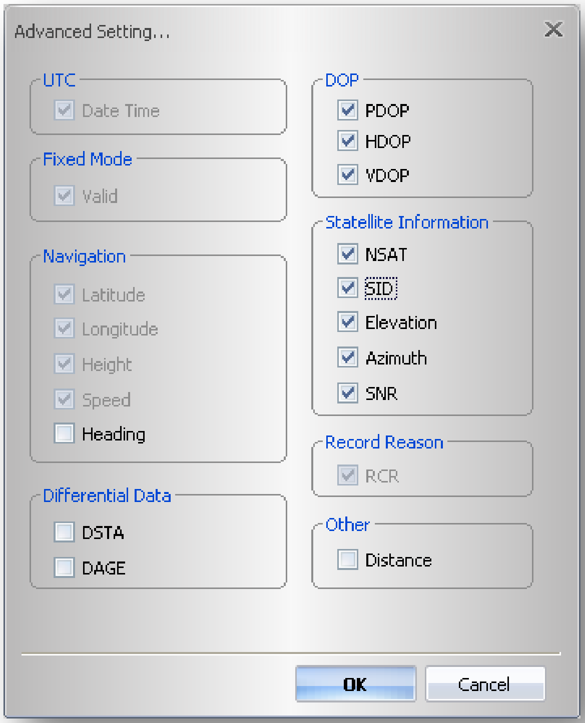

(c) Ensure PDOP, HDOP, VDOP, NSAT, SID, Elevation, Azimuth, SNR fields are checked.
(d) Click OK.

***Note**: SNR field will only be available once SID field is checked.*

6. When the GPS device is configured, it is recommended that you **place tape** over the mode switch to prevent the GPS device from being accidentally turned off.

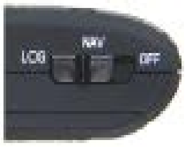

B.2 Downloading GPS Data

1. Connect GPS device via USB cord.
2. Verify that the GPS is in Log Mode.

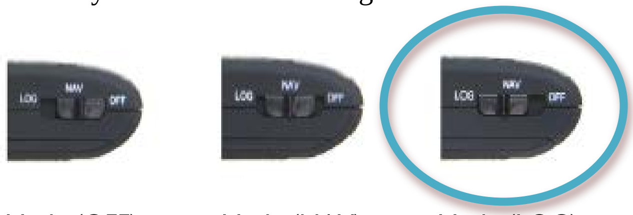

Mode (OFF) Mode (NAV) Mode (LOG)

3. Open QTravel.
4. Select Read Device Log from File menu.

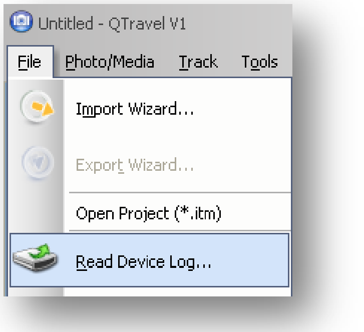

5. The following screens will appear in order.

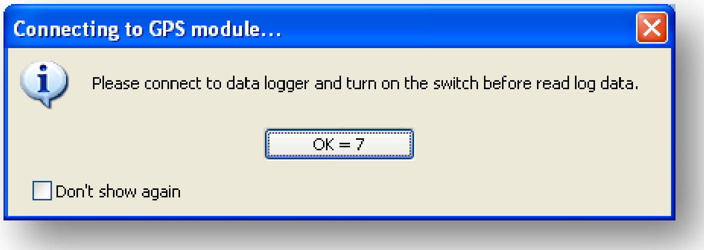

(a) Click OK.

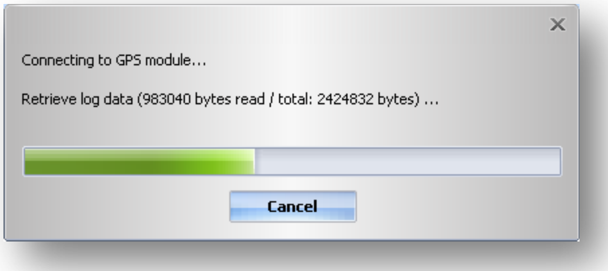

6. The screen will close when complete.
7. Be sure all Tracks are selected and click OK.

   (a) Import will start automatically.
   (b) Track logs will appear on the map (if connected to the Internet).

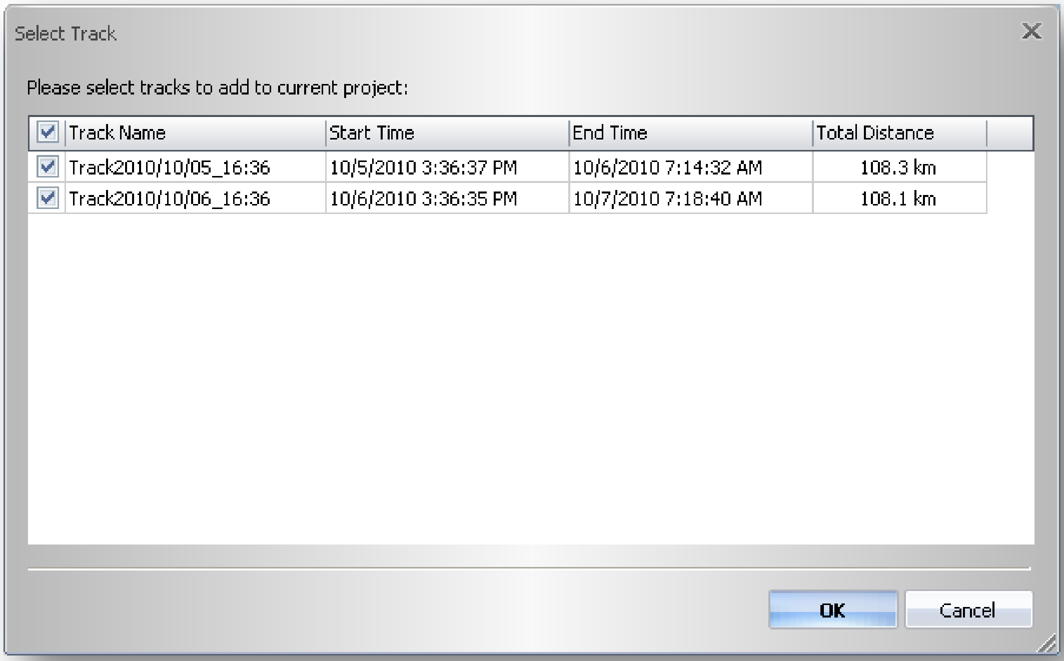

***Critical**: When data has been imported, the steps in the next section must be performed in order to save data!*

B.3 Exporting Raw Data

1. Select Raw Data Manager from the File menu.

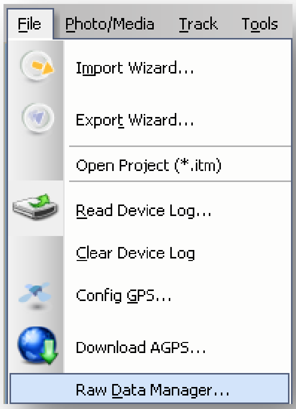

2. The following dialogue box will open.

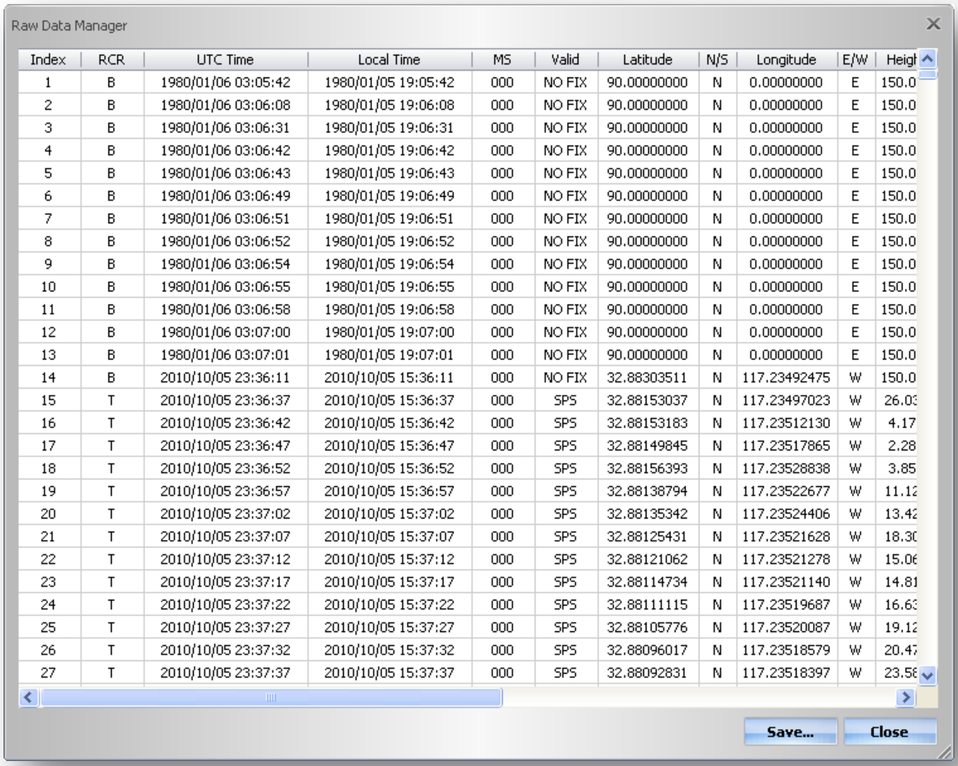

Index	RCR	UTC Time	Local Time	MS	Valid	Latitude	N/S	Longitude	E/W	Heigl
1	B	1980/01/06 03:05:42	1980/01/05 19:05:42	000	NO FIX	90.00000000	N	0.00000000	E	150.0
2	B	1980/01/06 03:06:08	1980/01/05 19:06:08	000	NO FIX	90.00000000	N	0.00000000	E	150.0
3	B	1980/01/06 03:06:31	1980/01/05 19:06:31	000	NO FIX	90.00000000	N	0.00000000	E	150.0
4	B	1980/01/06 03:06:42	1980/01/05 19:06:42	000	NO FIX	90.00000000	N	0.00000000	E	150.0
5	B	1980/01/06 03:06:43	1980/01/05 19:06:43	000	NO FIX	90.00000000	N	0.00000000	E	150.0
6	B	1980/01/06 03:06:49	1980/01/05 19:06:49	000	NO FIX	90.00000000	N	0.00000000	E	150.0
7	B	1980/01/06 03:06:51	1980/01/05 19:06:51	000	NO FIX	90.00000000	N	0.00000000	E	150.0
8	B	1980/01/06 03:06:52	1980/01/05 19:06:52	000	NO FIX	90.00000000	N	0.00000000	E	150.0
9	B	1980/01/06 03:06:54	1980/01/05 19:06:54	000	NO FIX	90.00000000	N	0.00000000	E	150.0
10	B	1980/01/06 03:06:55	1980/01/05 19:06:55	000	NO FIX	90.00000000	N	0.00000000	E	150.0
11	B	1980/01/06 03:06:58	1980/01/05 19:06:58	000	NO FIX	90.00000000	N	0.00000000	E	150.0
12	B	1980/01/06 03:07:00	1980/01/05 19:07:00	000	NO FIX	90.00000000	N	0.00000000	E	150.0
13	B	1980/01/06 03:07:01	1980/01/05 19:07:01	000	NO FIX	90.00000000	N	0.00000000	E	150.0
14	B	2010/10/05 23:36:11	2010/10/05 15:36:11	000	NO FIX	32.88303511	N	117.23492475	W	150.0
15	T	2010/10/05 23:36:37	2010/10/05 15:36:37	000	SPS	32.88153037	N	117.23497023	W	26.0
16	T	2010/10/05 23:36:42	2010/10/05 15:36:42	000	SPS	32.88153183	N	117.23512130	W	4.17
17	T	2010/10/05 23:36:47	2010/10/05 15:36:47	000	SPS	32.88149845	N	117.23517865	W	2.28
18	T	2010/10/05 23:36:52	2010/10/05 15:36:52	000	SPS	32.88156393	N	117.23528838	W	3.85
19	T	2010/10/05 23:36:57	2010/10/05 15:36:57	000	SPS	32.88138794	N	117.23522677	W	11.1
20	T	2010/10/05 23:37:02	2010/10/05 15:37:02	000	SPS	32.88135342	N	117.23524406	W	13.4
21	T	2010/10/05 23:37:07	2010/10/05 15:37:07	000	SPS	32.88125431	N	117.23521628	W	18.3
22	T	2010/10/05 23:37:12	2010/10/05 15:37:12	000	SPS	32.88121062	N	117.23521278	W	15.0
23	T	2010/10/05 23:37:17	2010/10/05 15:37:17	000	SPS	32.88114734	N	117.23521140	W	14.81
24	T	2010/10/05 23:37:22	2010/10/05 15:37:22	000	SPS	32.88111115	N	117.23519687	W	16.6
25	T	2010/10/05 23:37:27	2010/10/05 15:37:27	000	SPS	32.88105776	N	117.23520087	W	19.1
26	T	2010/10/05 23:37:32	2010/10/05 15:37:32	000	SPS	32.88096017	N	117.23518579	W	20.4
27	T	2010/10/05 23:37:37	2010/10/05 15:37:37	000	SPS	32.88092831	N	117.23518397	W	23.5

3. Click Save.
4. Verify the following boxes are checked and click OK.

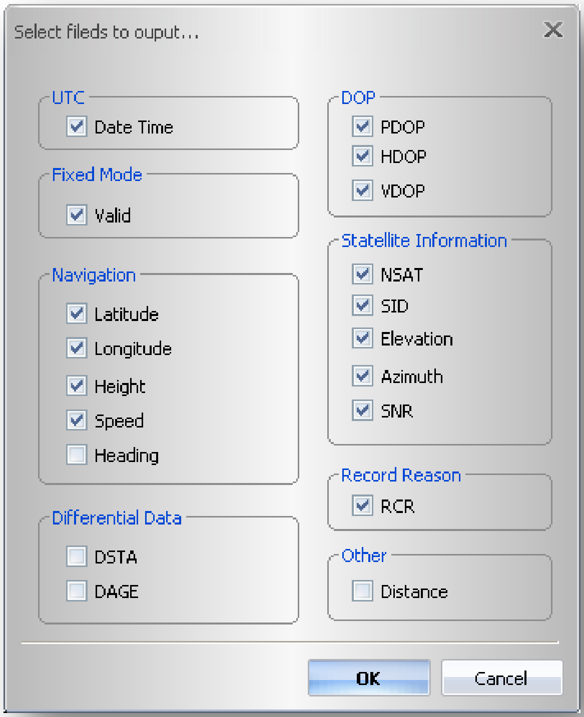

4. Name the GPS file (.CSV) and Save in the appropriate folder.

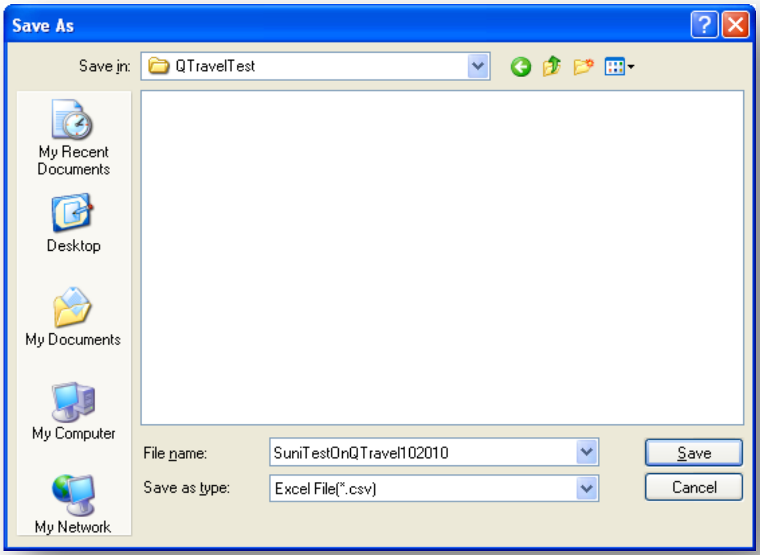

5. Click Close when finished.
6. Compare the rows of data in the CSV file to the rows of data in the Raw Data Manager. The CSV file should have one extra row of data (the header row). If there is a discrepancy in the number of rows of data, the device has been downloaded incorrectly. If so, close the program completely, turn off the device, and unplug the USB connector. Restart the program. Plug in the USB connecter and turn on the device. Then repeat the downloading procedure.

B.4 Exporting GPX Data

1. Select Export Wizard from the File menu.

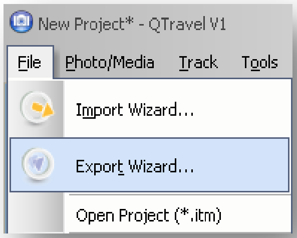

2. Select GPX File (GPS Exchange format).

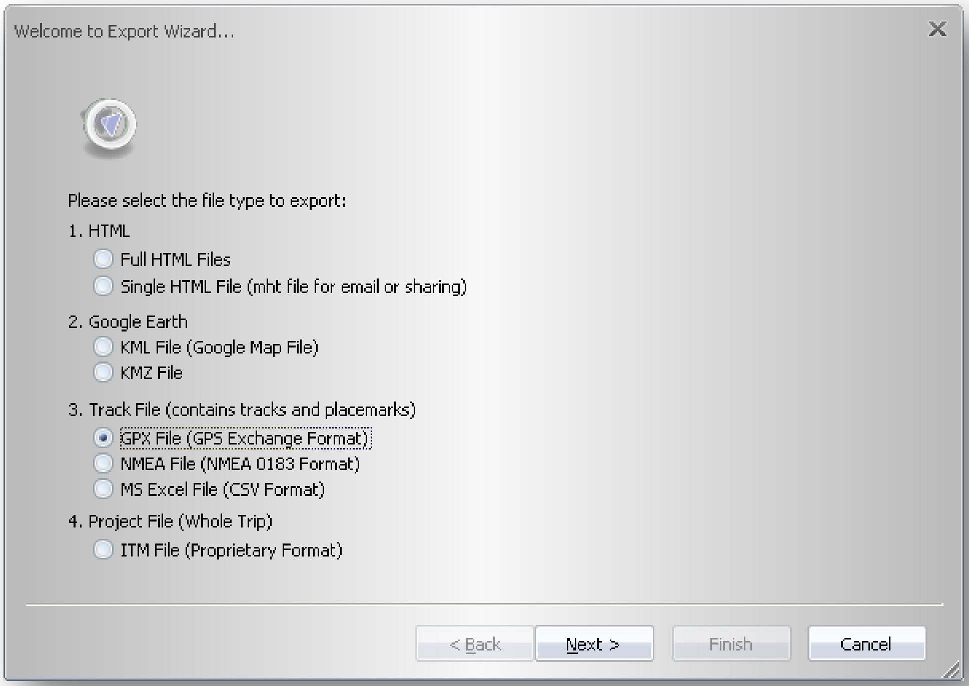

3. Click Next.
4. Check the box next to each record to export (typically, you will select all files).

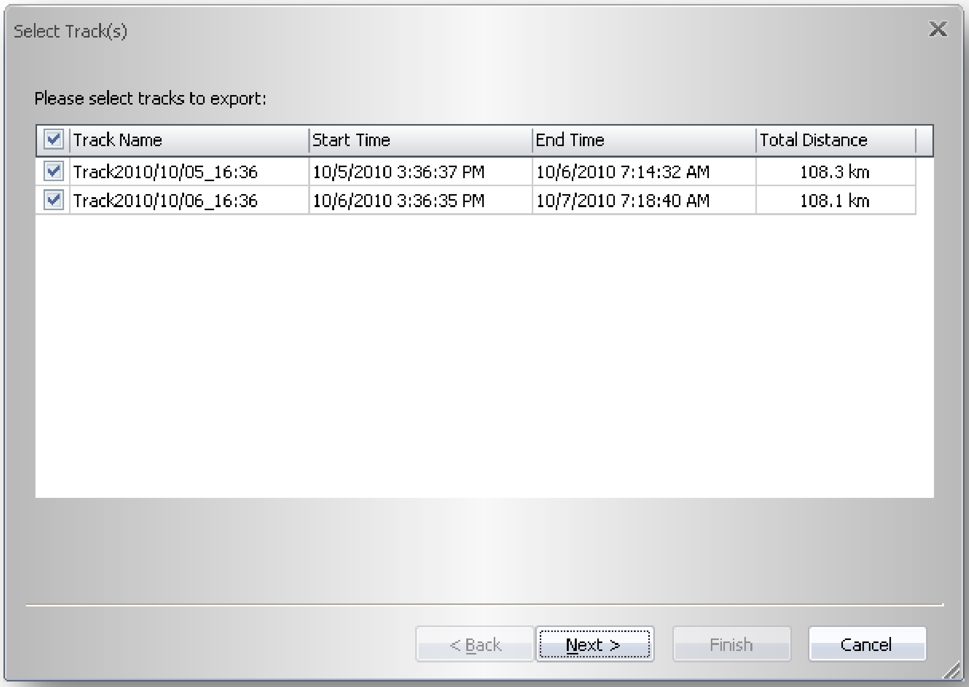

5. Click Next.
6. Select location to save files (verify that you are saving a .GPX file).

7. Name the GPX file (.GPX) and Save in the appropriate folder.

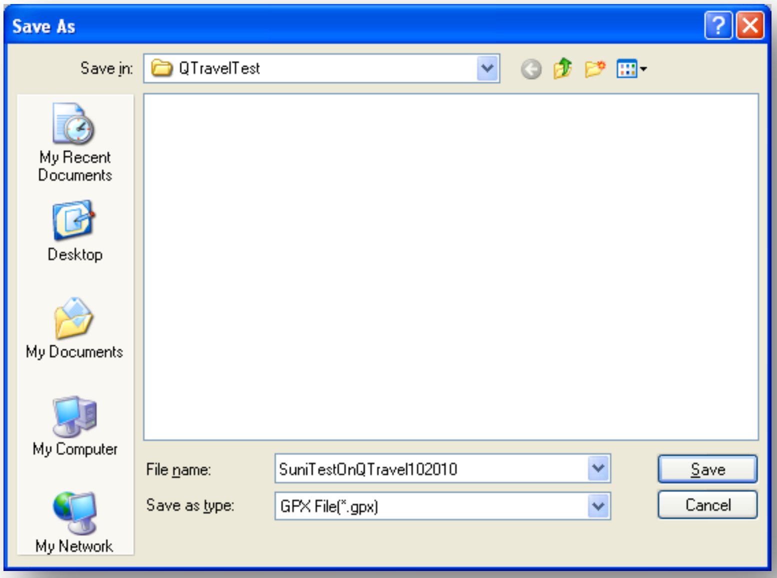

Appendix C: Guidelines for Using Google Earth with PALMS Output

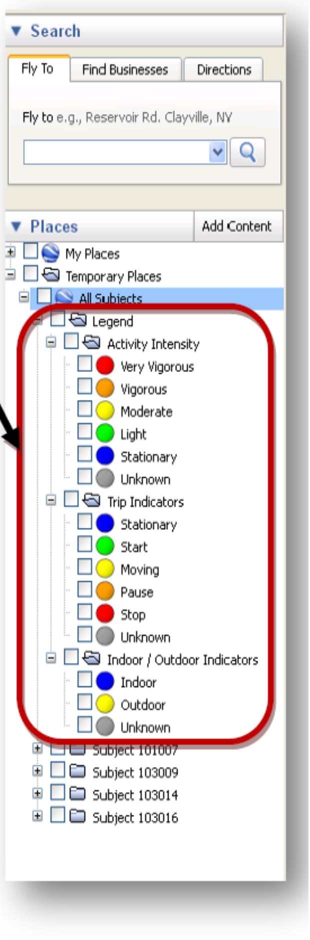

I. Open Google Earth
II. Open appropriate .KML file
III. Select subject information
   1. Open the Legend by checking the box next to the folder named Legend. This will expand showing you the various categories and color codes for each algorithm
   2. When you open the KML file, the Subject ID will be populated in the left hand menu, listed under "Temporary Places". Select appropriate Subject ID or IDs by checking or unchecking the box (all boxes are checked by default)

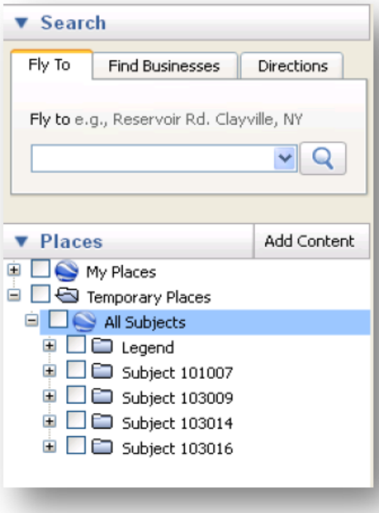

Note: You can save any "Temporary Place" file under "My Places" if you would like to eliminate the step of loading individual data files each time you open Google Earth.

3. Expand subject information by clicking on the **Plus Box** next to the Subject ID. This will show the available data, including a day-by-day list

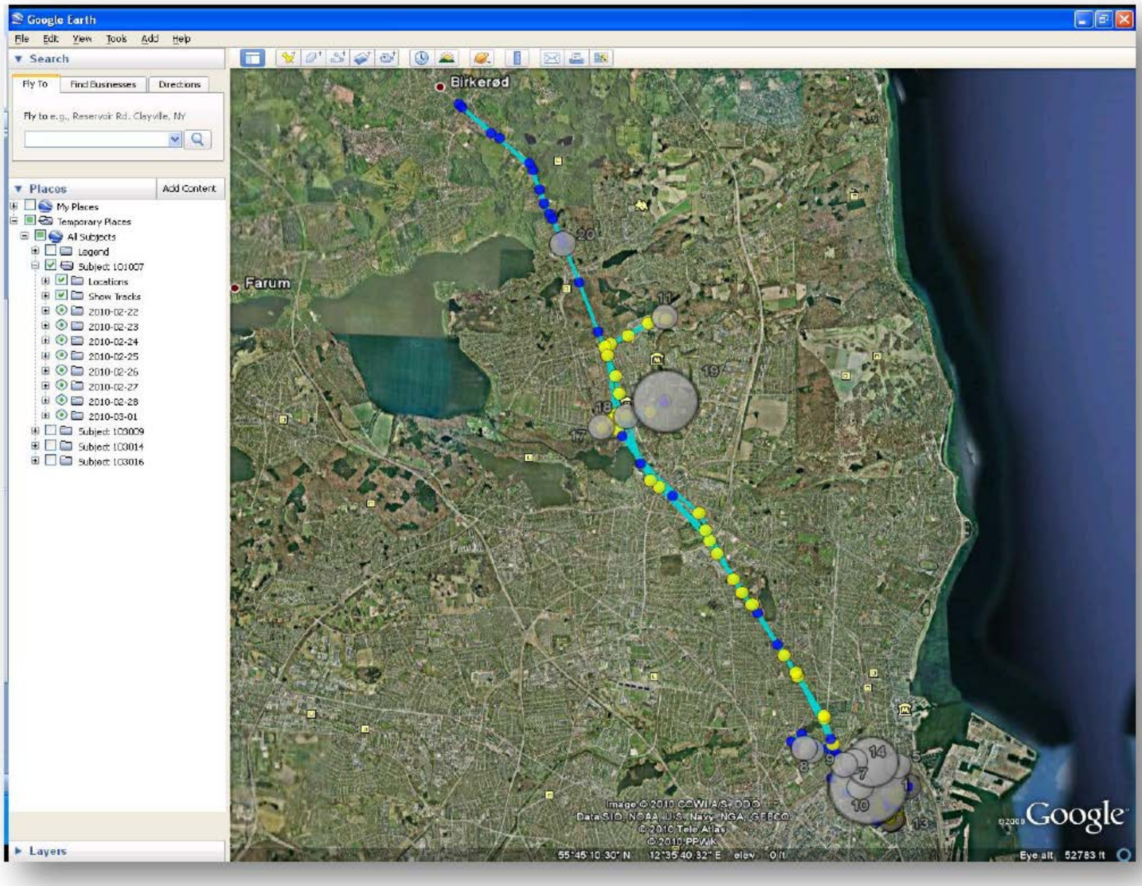

Note: All data will be selected by default and automatically plotted on the map. To remove data from the map, deselect unwanted categories (i.e., days) by clicking on the checked box next to each category.

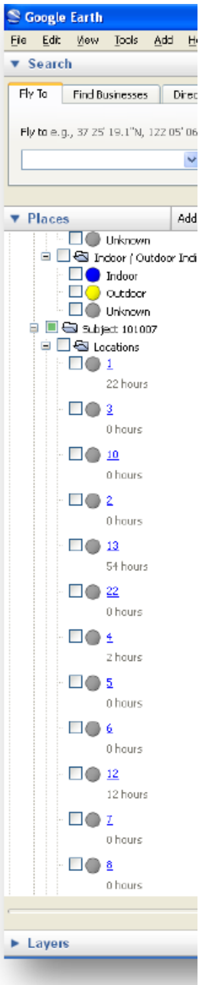

4. Identify day(s) of interest to the researcher conducting the PASTA interview(*reference Instruction guidelines)*
5. Deselect all unwanted days of data
6. Click on the Plus Box next to the day and category of interest to expand the selection

Note: If there is no **Plus Box** next to a specific category, there were no data available or no data were identified by PALMS. For example, if there is no Plus Box next to the "By Bouts" category, PALMS did not identify any bouts of activity that met the parameters set by the researcher during the processing of the data.

7. Available Categories of data:
   i. Locations (and hours at each location)
      1. Locations are identified based on the parameters set in PALMS. You can expand the category to locate a specific location of interest. The number of hours spent at each location is listed under each location number.
      2. Locations are marked on the map with a transparent grey circle.
   ii. By Trips
      1. Trips are identified by the parameters set from PALMS.
         a. First Fix = this is the first fix onto satellites BEFORE the start of the trip
         b. The folder icon under the By Trips category contains the various trips identified for that day for that participant. The **Tracks** icon can be checked to connect the trip points.

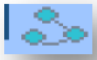

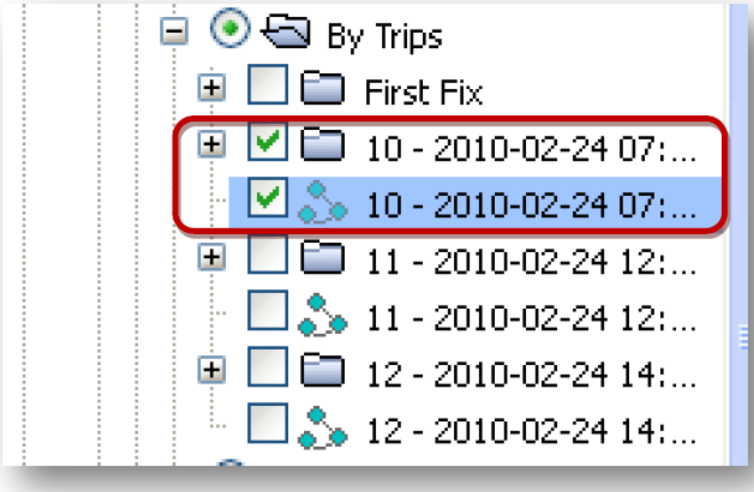

iii. By Bouts
    1. Bouts are identified by the parameters set from PALMS.

iv. Indoors/Outdoors
    1. PALMS will identify whether the data point at each location for each epoch is either indoors or outdoors based on satellite information exported from the GPS unit.

NOTE: not all GPS units can export this information

Appendix D. Importing PALMS CSV Output into ArcGIS
(by: Jasper Schipperijn, PhD)

PALMS CSV output is formatted based on US locale settings, which means that periods (".") are used as decimal separators and commas (",") as thousands separators. If you are in a country that uses other separators, please change the locale settings of your computer before import.

ArcGIS doesn't recognize periods (".") in the filename, you should therefore verify that filenames do not have them.

Each new map window in ArcGIS automatically takes the projection of the first layer that is added to the map. Adding a layer with a correct projection substantially reduces the chance for projection problems.

D.1 Add XY Data

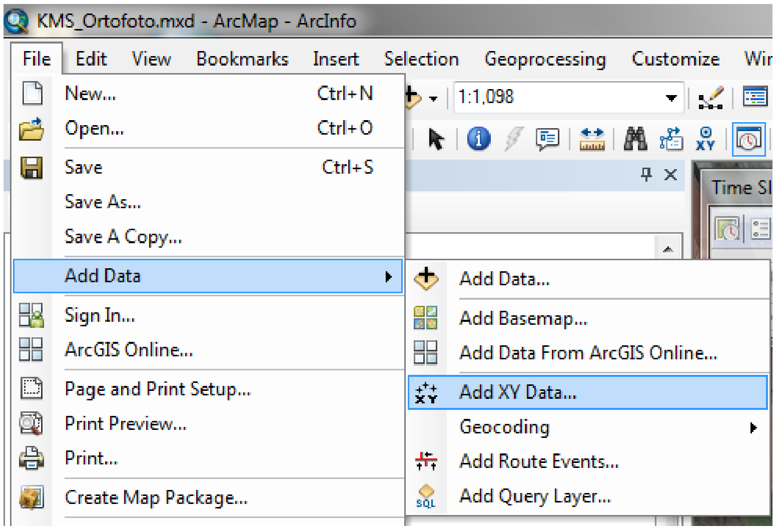

1. Select the file you want to add, and if needed, select the X and Y fields. ArcGIS will recognize the standard PALMS output field names "lon" and "lat".
2. Click Edit to set the correct coordinate system of the data to be imported.

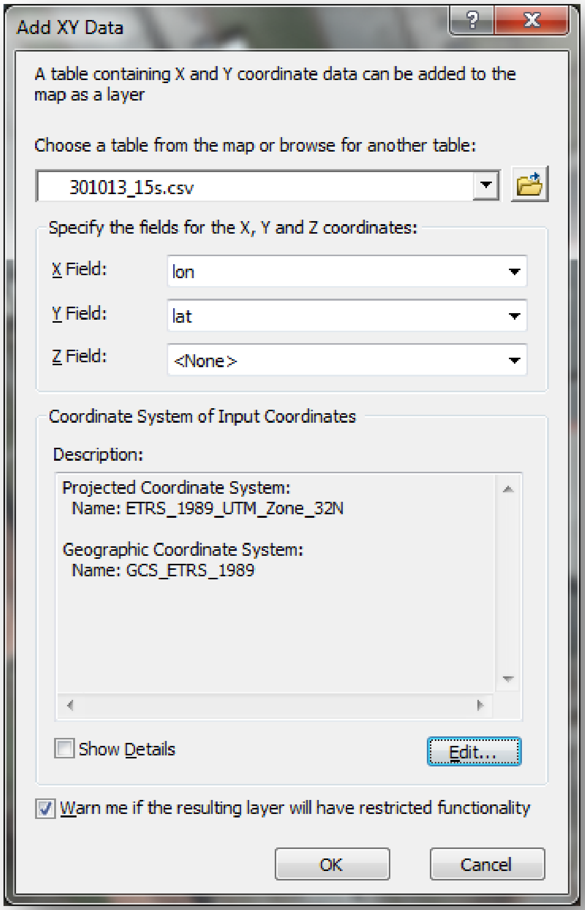

3. Click Select to set a predefined coordinate system

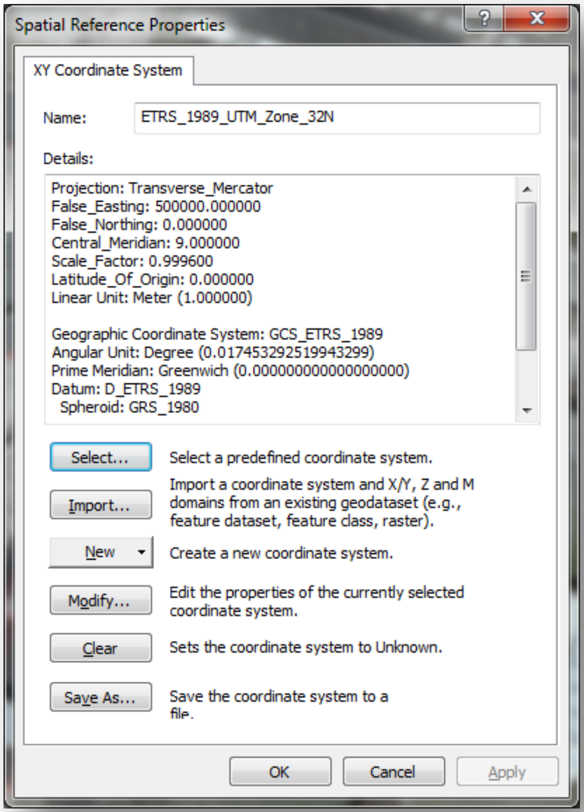

4. Click Geographic Coordinate Systems.

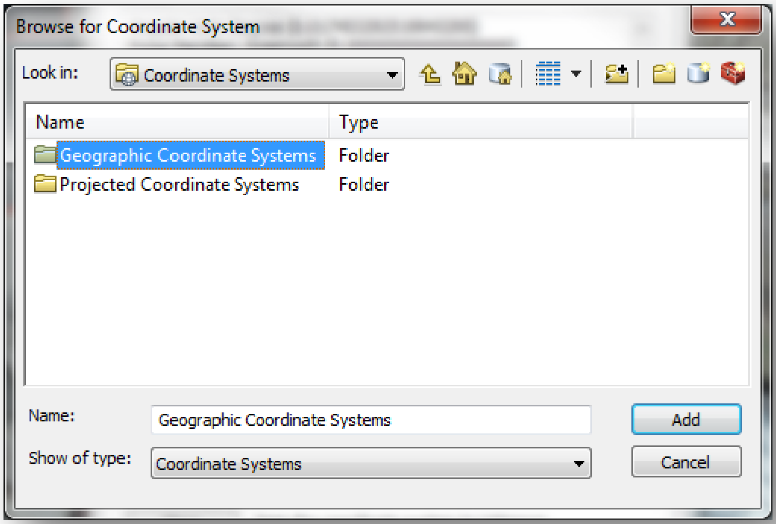

5. Click World.

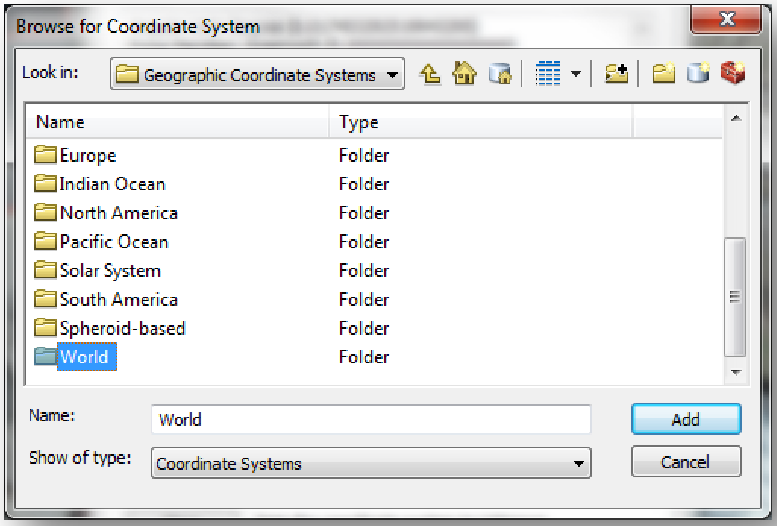

6. Click WGS 1984.prj. WGS 1984 is the projection for most GPS data. If your data is in a different projection, accordingly.

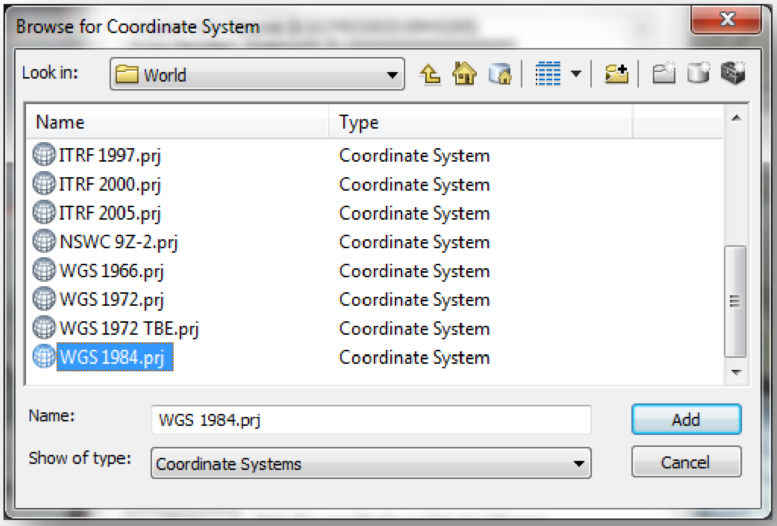

The window should now look like this (with your filename).

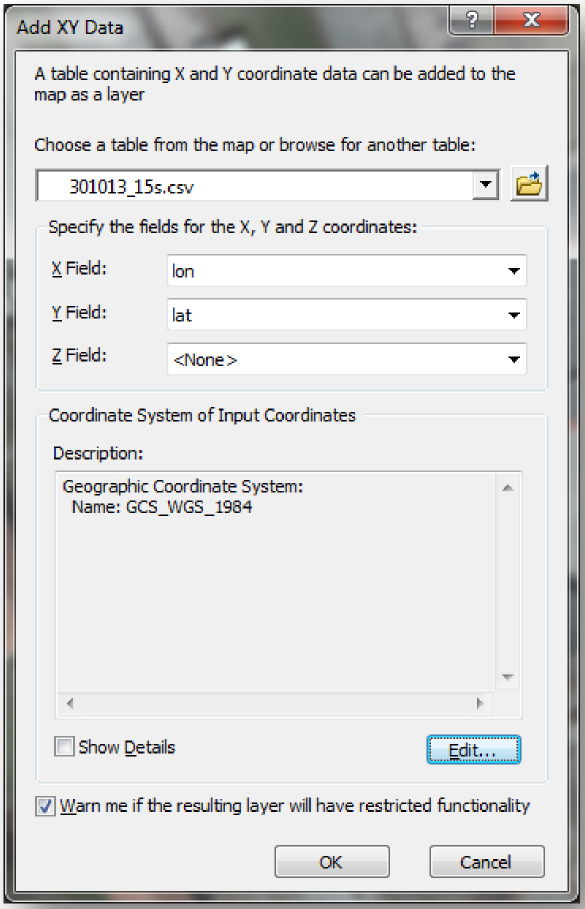

7. Click OK. Your data is now added to the map.

D.2 Exporting GPS XY Layer Files into an ArcGIS File Geodatabase

To increase processing speed in all future GIS analyses it is recommended to export all layers to a ArcGis File Geodatabase Feature Class.

1. Right click on the layer you want to export, click on Data, and click Export Data.

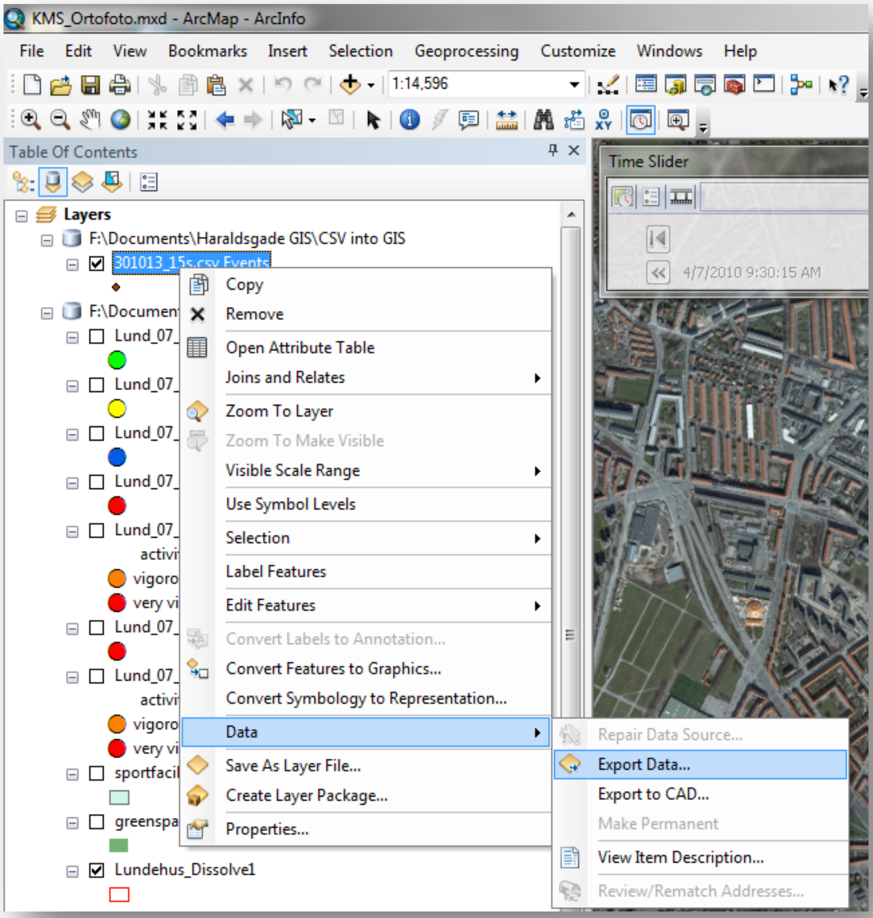

2. Select Use the Same Coordinate System As: The Data Frame. This will ensure correct projection of your GPS and will reduce the chance of subsequent projection problems.
3. Selected File and Personal Geodatabase Fature Classes in Save as Type.
4. Select the desired File Geodatabase.
5. Give the file a logical, recognizable filename that does not begin with a number.

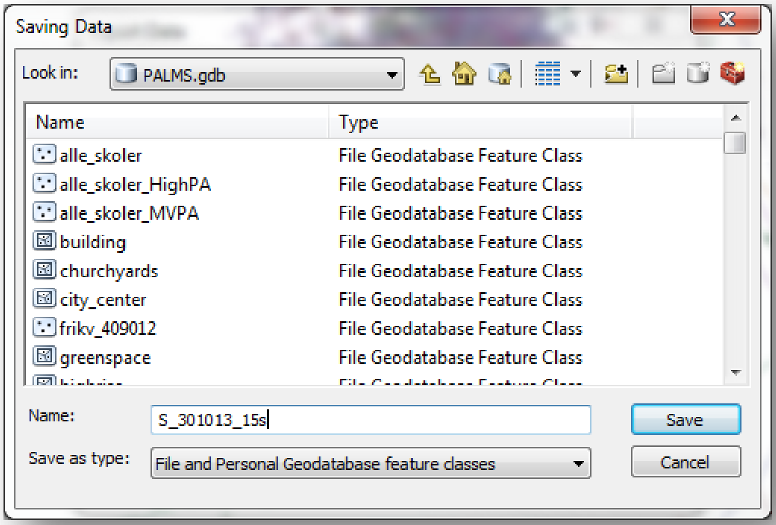
Saving Data
Look in:
PALMS.gdb
Name
Type
alle_skoler
alle_skoler_HighPA
alle_skoler_MVPA
building
churchyards
city_center
frikv_409012
greenspace
File Geodatabase Feature Class
Name:
S_301013_15s
Save
Save as type:
File and Personal Geodatabase feature classes
Cancel

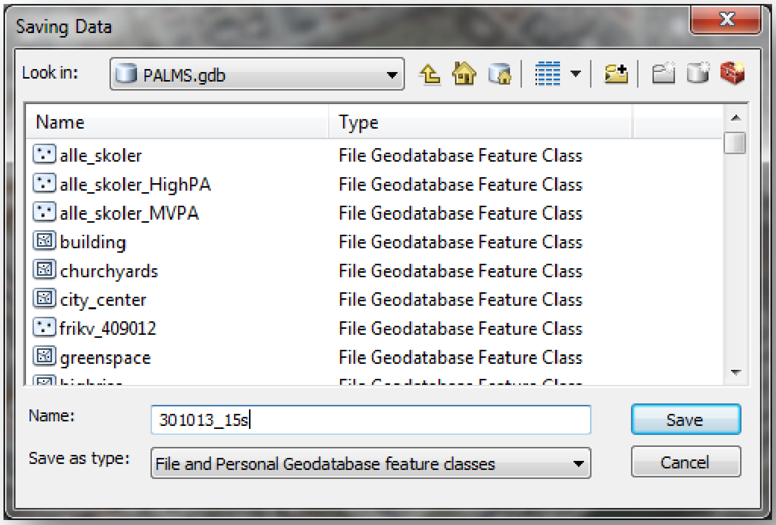
Saving Data
Look in:
PALMS.gdb
Name
Type
alle_skoler
alle_skoler_HighPA
alle_skoler_MVPA
building
churchyards
city_center
frikv_409012
greenspace
File Geodatabase Feature Class
Name:
301013_15s
Save
Save as type:
File and Personal Geodatabase feature classes
Cancel

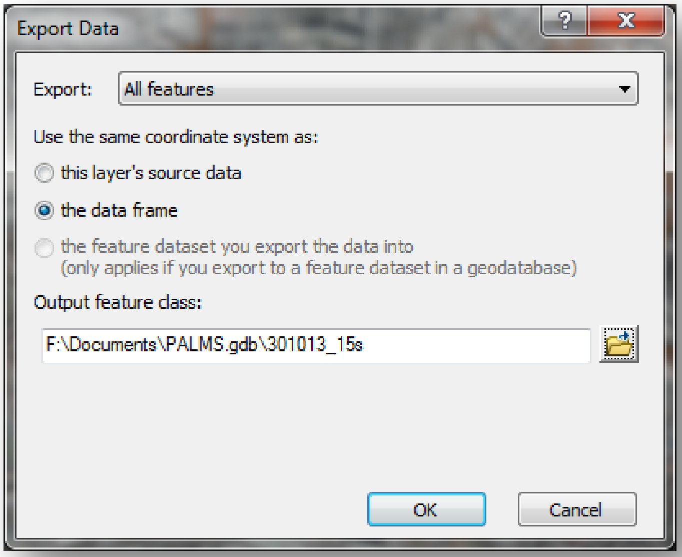

6. Click OK.

***Note**: Exporting time will depend on the amount of data and your computer speed. Large files (>1 million points) typically take at least a few minutes to export.*

9. If you want to add the data to the map, click Yes.

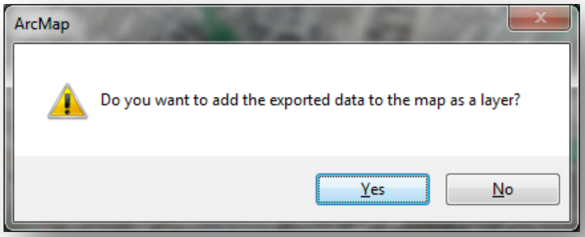

Appendix E. Examples of Participant Instructions (Wear and Charging)

Below are examples of wear instructions for participants.

E.1 Accelerometer Wear Instructions

1. Wear the monitor on the belt provided.
2. Keep the monitor on your **right hip** (see picture below).
3. Wear the monitor at all times **except** when sleeping, showering, or swimming.
4. Ensure that the belt is kept snug so that the monitor is firmly against your body and does not hang loosely.
5. The monitor is water resistant, **not** waterproof.
6. Do not wash the monitor or the belt. The belts will be cleaned when they are returned.
7 After wearing the monitor for 7 days, please return it in the self-addressed stamped envelope.

Questions? Call (858) 534-9311

If there is no answer, please leave a message and your call will be returned.

THANK YOU

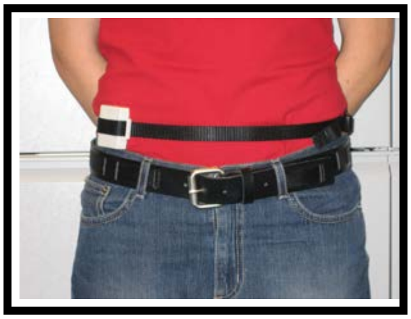

E.2 GPS Wear Instructions

GPS refers to Global Positioning System. The GPS unit will continuously record your position on the face of the earth as you move about during the day. We are asking you to wear this unit so that we can learn more about what environments people are physically active in. Here is a list of things that will help you remember what to do:

1. The UCSD Researcher will assist you to ensure that your device is turned on and working at each of your scheduled appointments.
2. Take the unit with you wherever you go.
3. Clip the unit directly to your clothing or movement meter belt, or carry it in your pocket, backpack or purse.
4. Don't let the GPS device get wet or bumped hard**.**

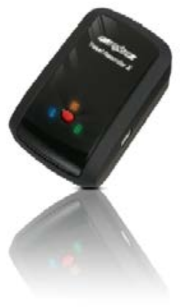

E.3 Charging the GPS Device

You **must** charge the GPS battery each evening.

1. Charge the GPS unit near your cell phone so that you remember to pick it up in the morning.

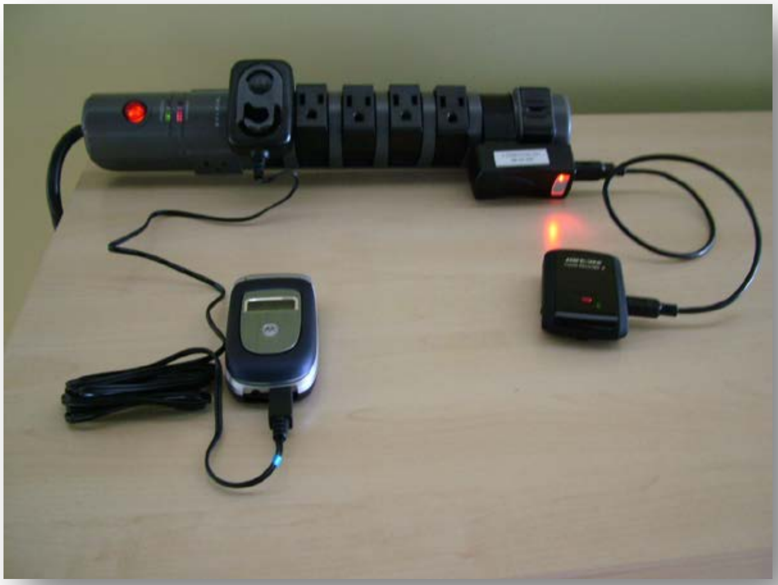

2. You can leave the GPS device in its pouch while charging.
3. Use the cable with a **Red Star label.**

4. Plug the cable into the slot on the side of the GPS device (there is only 1 slot).

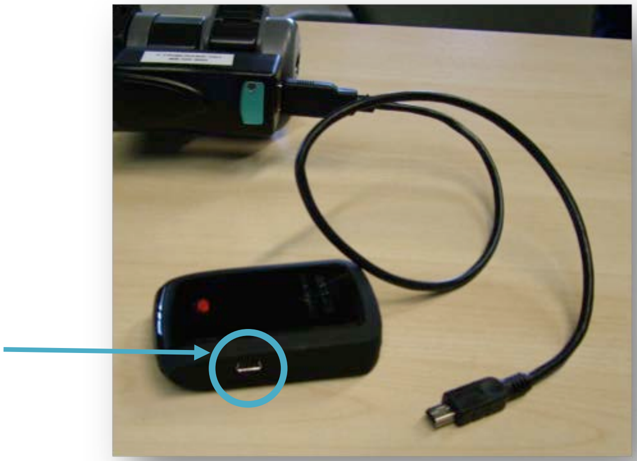

5. A green light indicates the battery is in charging mode
6. You do not need to move any switches on the device while charging.

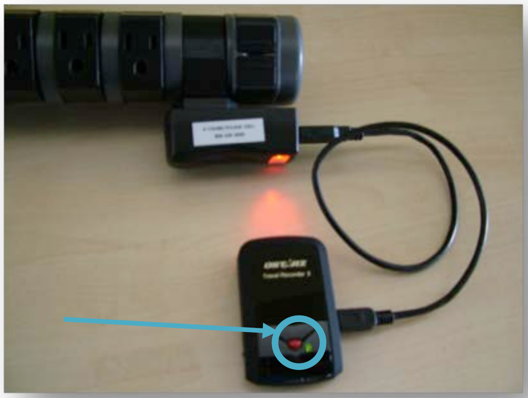

Questions? Call (858) 534-9311.

If there is no answer, please leave a message and your call will be returned.

E.3 Combined GPS and Accelerometer Wear Instructions

HOW TO WEAR THE DEVICE BELT

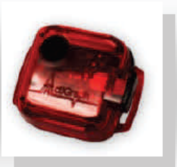

RED device

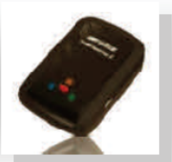

BLACK device

WEAR the device belt. . .

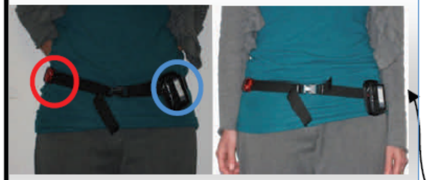

for AT LEAST 12 hours a day

REMOVE the device belt. . .

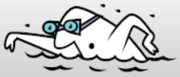

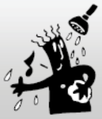

for water based activities

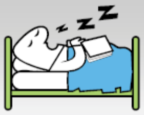

when you go to bed at night

CHARGE the BLACK device . . .

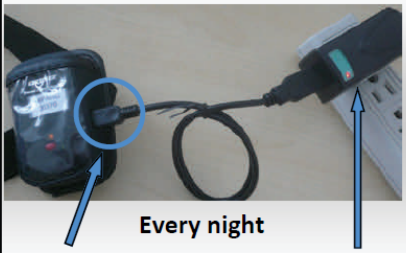

Every night

Plug device into charger

Plug charger into outlet

For this study we will ask you to wear 2 devices attached to a belt.

- the **RED device** measures the movement of your hips;
- the **BLACK device** measures location, speed and the distance you travel.

At first the belt may feel slightly awkward, but most people soon forget about it. It's extremely important that you wear the device belt <u>whenever you are awake</u>.

Proper Placement

The belt should be worn snug around your waist either over or under your clothing (whichever is more comfortable).

1. Position the **RED device** just above your RIGHT hip bone. (RED = RIGHT)
2. Position the **BLACK device** just above your LEFT hip bone. (BLACK = LEFT)

You can check the correct placement of both devices by letting your arms hang down by your sides. Each device should be in line with your arms, just touching your forearms

Tips for Wearing the Device Belt

- **Put it on first thing in the morning** -- either just after you get out of bed or just after you shower or take a bath in the morning.
- **Do not get it wet** (take it off while swimming, bathing, etc.)
- At night, take it off right before you go to bed and **charge the BLACK device.**
- There is no "ON" or "OFF" switch on either device.
- **Wear the belt for at least 12 hours every day** for the entire week. (If it is not worn long enough we will ask you to re-wear it).
- **Do not let anyone else wear the belt.** The devices have been programmed to work on you.

Charging the BLACK Device (every night)

- Leave the BLACK device on the belt inside the case.
- Plug charger into outlet and plug device into charger.
- In the morning, unplug the device from the charger.

What To Do if You Have Questions

- **Call us at (858) 822-3311**, if you have any questions.
- Please leave a message if you reach the voice mail, and someone will return your call.

Appendix F. PASTA Protocol

PASTA (Physical Activity Spatial - Temporal Analysis)
Instruction Guidelines

Sheet preparation instructions:

1 Print appropriate numbers of PASTA sheets
(you may need multiple sheets per day depending on number of participant trips)

2 Enter participant ID and date of administration at the top of each page

3 Enter day and date the participant will be recalling

4 Fill in times as needed along the left hand column labeled **'Time'**

5 Ensure you have the appropriate data source to guide your questioning
(i.e., Google Earth KML files opened in Google Earth) including blank maps (if necessary)

* *Depending on what information is available before visit, much of this sheet can be pre-populated with participant trips, wear times, data anomalies etc. - the questions can confirm this entry*

PASTA administration instructions:

1 Use the detailed data source output files (i.e., KML file on Google Earth, accelerometer graphs, etc.) as a guide for this structured interview. The visualizations should help the researcher identify obvious areas of activity. The objective of this interview is to confirm and identify "events" that are of interest to the study (i.e., wear times, trip identification, mode identification, etc.).

2 You may either ask the participant to simply recall his or her activities in the day and record the responses or you can prompt the participant with the detailed source files (i.e., KML files, graphed DAT files, etc.).

3 When asking about trips or bouts of activity, be cautious about showing visualizations (i.e., Google Map) that contain possible "errors" or "anomalies". These may confuse the participant if s/he is struggling to remember the events of the day.

4 If the participant struggles recalling long periods in the day, try to cue his or her recall by asking generic questions about the day. For example, if the participant cannot remember an activity in the early evening, ask questions about what s/he had for dinner. Or if a participant knows that s/he took the devices off, but can't remember when, ask questions about bathing (especially since bathing often will follow a bout of physical activity).

5 It is generally recommended to start with the most recent day of data and work backwards. For example, if you have data from Monday through Friday, start in the evening on Friday since this will usually be the easiest for a participant to recall.

Please make sure you document *at least* the following information on the attached PASTA record sheet (for each day):

1 What time were the devices first put on?

2 What time were the devices last taken off?

3 Were the devices removed for any reason during the day?
Record time on, time off for each occurrence

4 Confirm Trips as listed in Google Earth

- *Start and end times and location*
- *Type of activity*
- *Route*
- *Trip purpose (i.e., exercise, walk to dinner, etc.)*
- *Trip mode*

5 Indicate when participant was indoors or outdoors

6 Ask if participant was exposed to any type of smoke during trips (i.e., cigarette smoke, fireplace, etc.)

7 Record any bus trips and purpose

8 Record any data anomalies (i.e., HR drop out)

9 Record any other notes and comments on PASTA record sheet

Other suggestions for data collection:

1 Record specific bouts of activity

2 Record who the participant was with during specific activities of interest

3 Ask participant about specific cycling activity (this is most applicable when only collecting accelerometer data since cycling is often not accurately identified).

PALMS PASTA

Participant ID 31

Date of Assessment 28-Jul

Date **7/26/2009**

Day **Sunday**

**Time**	**Activity Description** (i.e., Walking, Driving to Market, etc, devices removed for shower)	**Trip #**	Notes/Comments/Questions (i.e., indoor/outdoor, etc.)
6:30:00 AM	Put on devices		Indoor
6:45:00 AM	Went for walk.	50	Outdoor: Went down the hill from Casa de Campanas, stopped at Bird Creek (at 7:30 and 7:50) and turned around at an Oak tree to follow the same route back.
8:30:00 AM	Packed up to go to craft room for saxophone practice	51/52	Indoors: home to craft room
9:40:00 AM	Saxophone practice	51/52	Craft room (Location **of activity does not match up with actual location)**
10:00:00 AM	Finished saxophone practice	52/53	Indoors: craft room to home
10:50:00 AM	Prepared lunch and then ate lunch at 11:00 AM	54?	Indoors: home (**location of activity did not match reported home location)**
11:40:00 AM	Read outside on home porch.	54/55?	Indoors: home (**activity did not match reported home location)**
11:50:00 AM	Napped		Indoors: bedroom
1:30:00 PM	Worked on computer and visited a friend in the Health Center (between hours of 1 and 3)	57/58	Indoors: home to health center
2:15:00 PM	Visited a friend at the Health Center	57/58	
3:30:00 PM	Returned from Health Center and sat on porch and had snack	58?	Indoors: porch at home **(activity did not match location at home; data indicates one building away from home)**
4:00:00 PM	Walked to dining hall and ate dinner	59	Indoors: home to dining hall
4:50:00 PM	Sat/relaxed in dining hall	No trip detected	Indoors; dining hall
5:10:00 PM	Returned home	No trip detected	Indoors
6:50:00 PM	Watched TV	No trip detected	Indoors: (a trip was detected again at approximately 9:02 PM)
9:16:00 PM	Took off device		

PALMS PASTA

Participant ID ______________

Date of Assessment ______________

**Date**	
**Day**	

**Time**	Activity Description (i.e., Walking, Driving to Market, etc, devices removed for shower)	Trip #	Notes/Comments/Questions (i.e., indoor/outdoor, etc.)

Appendix G. Parameter Quick Reference Guide

G.1 General Information Section

Parameter name	Usage
Participant	Selects one individual participant or all participants to be included in the calculation
Start Date	Earliest date to be included
End Date	Latest date to be included
Interval (in seconds)	Length of interval used to summarize the data
Result Name	Name under which the results of the calculation will be stored

G.2 GPS Tab

Functional block	Parameter name	Usage
Invalid values filter		
	Filter invalid	Values: 0 = filter (remove) invalid points from the calculation, *1 = mark points*, 2 = do not filter or mark
	Max speed (Km/hr)	invalid if fix has speed greater than this value
	Max change in elevation (meters)	invalid if fix as elevation delta greater than this value over the interval
	Max change in distance (meters)	invalid if change in distance greater than this value over interval *Not Currently Used*
	Min change in distance (meters)	discard if change in distance is less than this value over the interval
	Max loss of signal (in seconds)	declare loss of signal when time between fixes exceeds this value
	Filter lone fixes	When checked, filters fixes when the time between fixes indicates loss of signal.
Indoors detection		
	Detect indoors	When checked, mark fixes as indoors or outdoors
	Max satellite ratio when indoors	Maximum ratio of satellites-used / satellites-in-view to be considered indoors. Values greater than this are marked as outdoors.
	Max SNR value when indoors	Maximum signal-noise value to be considered indoors. Values greater than this are marked as outdoors.

Apply averages to		
	Compute average speed	When checked, computes and presents speed as a moving average over N GPS samples.
	Compute average elevation	When checked, computes and presents elevation delta as a moving average over N GPS samples
	Number of GPS samples to average	Number of samples (N) used to compute the moving average
Location detection		
	Detect location clusters before trips	When checked, location clusters are detected before trips detection (Trips are considered was occurring between locations. However, this does not work well in restricted geographic spaces, such as school campus and retirement communities.)
	Include trip pause locations	When check, pauses occurring during a trip are included as locations.
	Trap points within location	When checked, all GPS fixes within a given radius of the location will be set the the center of the location.
	Cluster radius (meters)	Defines radius of location in which fixes are trapped
	Min time at location	Minimum amount of time that must be spent at a location for it to be considered a location
Trip detection		
	Detect trips	When checked, trips are detected, numbered and classified
	Min change in distance (meters)	Minimum distance that must be traveled over one minute to indicate the start of a trip. (Note: 1 km/h = 17 meters/minute)
	Min trip length (meters)	Trips less than this distance in length are not classified as trips.
	Min duration for trips (seconds)	Trips less than this duration are not classified as trips.
	Min pause time (seconds)	When duration at point exceeds this value, point is marked as a pause point.
	Max pause time (seconds)	When duration of pause exceeds this value, point is marked as a end point.
	Allow trips within a single locations.	When checked, allow a trip to occur solely within one location. (By default, such trips are removed because such trips are hard to distinguish from GPS "noise" .
Speed cutoff values		
	Vehicle speed cutoff (KMph)	Speeds greater than this are marked as vehicle
	Bicycle speed cutoff (KMph)	Speeds greater than this are marked as bicycle
	Running speed cutoff (KMph)	Speeds greater than this are marked as running - not yet implemented
	Jogging speed cutoff (KMph)	Speeds greater than this are marked as jogging - not yet implemented
	Walking speed cutoff (KMph)	Speeds greater than this are marked as walking - not yet implemented
	Sedentary speed cutoff (KMph)	Speeds greater than this are marked as pedestrian

G.3 Accelerometer Tab

Functional block	Parameter name	Usage
Not wearing time		
	Mark not wearing	When checked, mark not wearing time
	Minutes of zeros in a row	Number of consecutive minutes of zeros that must be present for period to be marked as not wearing.
Bouts detection		
	Detect activity bouts	When checked, detect bouts of activity
	Activity bout length (minutes)	Minimum duration of bout
	Activity bout upper limit (counts)	Maximum limit of activity to be considered part of a bout
	Activity bout lower limit (counts)	Minimum level of activity to be considered part of a bout
	Activity bout tolerance (minutes)	Number of consecutive minutes allowed outside of the activity limits that will still be considered part of a bout
Sedentary bouts detection		
	Detect sedentary bouts	When checked, detect bouts of sedentary periods (non-activity)
	Sedentary bout length (minutes)	Minimum duration on sedentary bout
	Sedentary bout upper limit (counts)	Maximum level of activity to be considered sedentary
	Sedentary bout tolerance (minutes)	Number of consecutive minutes of activity allowed during sedentary bout
Cutoff values		
	Very hard (counts)	Counts greater than this value will be classified as very hard (very vigorous)
	Hard (counts)	Counts greater than this value and less than the above value will be classified as hard (vigorous)
	Moderate (counts)	Counts greater than this value and less than the above value will be classified as moderate
	Light (counts)	Counts greater than this value and less than the above value will be classified as light. NOTE: Counts less than this value will be classified as sedentary.

G.4 Heart Rate Tab

Functional block	Parameter name	Usage
Include heart rate calculation		
	Include heart rate	When checked, heart rate is included in calculation.
Invalid values filter		
	Filter invalid values	When checked, replace invalid heart rate with moving average or last valid heart rate
	Max number replaced	Maximum number of samples that would be replaced before declaring invalid
	Min valid rate (bpm)	Minimum heart rate allowed in beats per minute
	Max valid rate (bpm)	Maximum hear rate allowed in beats per minute
Moving average		
	Compute moving average	When checked, computes and presents heart rate as a moving average over N samples
	Number of samples to average	Computes moving average over this number of sample (N)
Bouts detection		
	Detect bouts	If checked, detect bouts of elevated heart rate
	Bout length (minutes)	Minimum duration of bout
	Upper limit (percentage of max HR)	Maximum heart rate to be considered part of a bout
	Lower limit (percentage of max HP)	Minimum heart rate to be considered part of a bout
	Bout tolerance (minutes)	Number of consecutive minutes outside of the limits allowed in the bout
Max HR formula		
	Max HR Formula	Formula used to compute maximum heart rate based on participant's age<br>Possible choices are:<br>Default, Londeree, Miller, Kavonen
	Default age	Age to be used when participant's age is not known (defaults to 30)
Cutoff values		
	Heart rate max effort (percentage of maximum heart rate)	Percentages greater that this number will be classified as max effort
	Heart rate anaerobic (percentage)	Percentages greater than this number and less than the above will be classified as anaerobic
	Heart rate aerobic (percentage)	Percentages greater than this number and less than the above will be classified as aerobic
	Heart rate weight control	Percentages greater than this number and less than the above will be

(percentage)	classified as weight control
Heart rate moderate (percentage)	Percentages greater than this number and less than the above will be classified as moderate
Heart rate light (percentage)	Percentages greater than this number and less than the above will be classified as light.
Heart rate resting (percentage)	Percentages greater than this number and less than the above will be classified as resting. Percentages less that this value will be marked as invalid.

G.5 EE (Energy Expenditure) Estimate Tab

Functional block	Parameter name	Usage
EE Formula		
	Formula	Select formula used to estimate energy expenditure:<br>None -- do not estimate EE<br>Device Default -- use the EE value estimated by the device's software and uploaded to PALMS (accelerometer / heart rate monitor)<br>Freedson -- (for ActiGraph devices only) - use Freedson formula based on counts and partipicant's weight<br>Work-Energy Theorem -- (for ActiGraph devices only) - use Work-Energy Theorem formula based on counts and participant's weight<br>Combination -- (for ActiGraph devices only) - use a combination of the Freedson and WET formula based on the combination cutoff value (see below).
	Combination Cutoff Value	When Combination Formula is selected:<br>For counts below this value, use WET. For counts greater or equal to this value, use Freedson
	Branched Equation	*Not yet implemented*

G.6 Merge Options Tab

Functional block	Parameter name	Usage
Merge Options		
	Merge data to GPS	When checked, activity and heart-rate data is aligned to the GPS timeline. Data without GPS fixes are discarded.
	Start first day at midnight	When checked, the timeline for the first day of data starts at midnight. Otherwise, it starts at the timestamp of the first data sample.
	Insert missing fixes	When checked, gaps in GPS fixes are replaced by the last valid fix.

Appendix H. PALMS CSV Output Dictionary

GPS, Activity, HR – Process and Merge (R2) Calculation

Variable Name	Values and Descriptions
activity	-2 – non-wear time<br>-1 – missing data<br>0 - ∞ - Count values from device
activityBoutNumber	Sequential Integer values for each participant based on the bout occurrence (ie. The first bout for a participant will be assigned a value of one).<br><br>Zeros represents no bout detected
activityIntensity	Activity intensity categories:<br>-2 – non-wear time<br>-1 – missing<br>0 – sedentary activity<br>1 – light activity<br>2 – moderate activity<br>3 – hard activity<br>4 – very hard activity
dateTime	Date and time for the data point<br>Format: mm/dd/yyyy hh:mm:ss
distance	Distance traveled in meters between consecutive points (current row and previous row)
duration	Time between consecutive points in seconds.
ee	Energy Expenditure estimate<br><br>-1 – missing
ele	Elevation (meters)
fixType	String that show different classification by PALMS. These classification are not mutually exclusive and are concatenated with a '+' symbol.

Value	Classification
firstfix	marked as first fix
lastfix	marked as last fix
lastvalidfix	point is inserted from a last fix
indoors	marked as indoors
outdoors	marked as outdoors
stationary	marked as stationary
startpoint	marked as the start of a trip
midpoint	marked as part of a trip
pausepoint	marked as a pause during a trip
endpoint	marked as the end of a trip
clustered	point is part of location and the latitude and longitude has been changed to the cluster_center point
cluster_center	Point is part of location and is the center of the location. It's latitude and longitude will be used

	<table><tr><td></td><td>for points that are part of the same location, when the trap point parameter is marked.</td></tr><tr><td>inserted</td><td>GPS raw data point was missing or removed during the calculation and the insert missing fixes parameter was marked, so a nearest previous valid point has been inserted.</td></tr></table>
heartrate	Heart rate from monitor<br>-1 – Missing value
hrBoutNumber	Sequential number indicating a bout of activity based on heart rate<br>-1 – Missing value<br>0 – Point is not part of a bout
hrIntensity	Heart rate Intensity<br>-1 – missing data<br>0 – Resting<br>1 – Light<br>2 – Moderate<br>3 – Weight Control<br>4 – Aerobic<br>5 – Anaerobic<br>6 – Max Effort
identifier	Participant Identifier
lat	Latitude coordinate<br>Northern hemisphere – positive values<br>Southern hemisphere – negative values
locationNumber	Sequential number indicating a location<br>-1 – when on a trip<br>Location and trip numbers are not mutually exclusive.
lon	Longitude coordinate<br>Eastern hemisphere – positive values<br>Western hemisphere – negative values
nsatUsed	Number of satellites used to calculate coordinates
nsatView	Number of satellites in view when coordinates where calculated
snrUsed	Signal-to-Noise Ratio (SNR) of the satellites used to calculated coordinates
snrView	Signal-to-Noise Ratio (SNR) of the satellites in view when the coordinates were calculated
speed	Traveling Speed in kmph between consecutive points (current row and previous row)
tripMode	Transportation designation based on average speed of trip:<br>stationary<br>pedestrian<br>bicycle<br>vehicle
tripNumber	Sequential number indicating a trip for each participant.<br>0 – Trip was not detected.
