# Supplementary material for: Location-specific psychosocial and environmental correlates of physical activity and sedentary time in young adolescents: preliminary evidence for location-specific approaches from a cross-sectional observational study
Source: Int J Behav Nutr Phys Act. 2022 Aug 26;19:108. doi: 10.1186/s12966-022-01336-7 (PMC9419353; doi:10.1186/s12966-022-01336-7)
Supplement: Supplementary file 2 — Additional file 2: Supplementary Table 1. Items categorized by scales and indices. Includes table which was too long to submit in the manuscript text. [file 12966_2022_1336_MOESM2_ESM.docx]

| Supplementary Table 1.  *Items categorized by scales and indices* | | |
| --- | --- | --- |
| Scale/Index | Item Stems and Scale of Responses | Items |
| **General variables** |  |  |
| Physical activity self-efficacy | - HOW SURE are you that you can do physical activity in each situation? - 5-point Likert Scale (1 = *I’m sure I can’t*, 5 = *I’m sure I can*) | - Do physical activity even when you feel sad or stressed - Do physical activity even when your family or friends want you to do something else - Do physical activity even when it is raining or really hot outside |
| Physical activity enjoyment | - No stem - 5-point Likert Scale (1 = *Strongly disagree*, 5 = *Strongly agree*) | - I enjoy doing physical activity. |
| Physical activity pros | - Please circle the answer that best applies to you when deciding whether or not to do physical activity. - 5-point Likert Scale (1 = *Strongly disagree*, 5 = *Strongly agree*) | - Physical activity would help me stay fit. - My parents would be happy if I did physical activity. - I would feel better about myself if I did physical activity. - I would have fun doing physical activity or playing sports with my friends. - I would have more energy if I did physical activity. |
| Physical activity cons | - Please circle the answer that best applies to you when deciding whether or not to do physical activity. - 5-point Likert Scale (1 = *Strongly disagree*, 5 = *Strongly agree*) | - I would feel embarrassed if people saw me doing physical activity. - There is too much I would have to learn to do physical activity. - I do not like the way physical activity and exercise makes me feel. - Physical activity takes time away from being with my friends. |
| Physical activity social support | - During a typical week: - 5-point Likert Scale (0 = *Never*, 4 = *Very Often*) | - How often does an adult in your household encourage you to do sports or physical activity? - How often do your brothers/sisters or friends do physical activity or play sports with you? |
| General physical activity psychosocial index | Index of the scales above | |
| **Location-specific school variables** |  |  |
| PE and recess time | - No stem - Minutes of PE per week - Minutes of recess per week | - How many days per week do you have gym or PE class at school? If you have PE, on average, how long is each PE period? - How many days per week do you have recess at school? If you have recess, how long is the total time spent in recess? |
| After school environment | - No stem - 5-point Likert Scale (0 = *Never*, 4 = *Always*) | - How often does your school have supervised physical activities after school? - How often does your school allow students to use play areas or fields after school? |
| School physical activity equipment | - Do you have any of these at your school? - Dichotomous yes/no scale (0 = *No*, 1 = *Yes*) | - Basketball hoops - Soccer goal posts - Baseball backstop - Running/walking track - Weight-lifting machines - Indoor exercise machines such as treadmills/stair climbers |
| School physical activity environment index | Index of the scales above | |
| **Location-specific non-school variables** |  |  |
| Sedentary reduction self-efficacy | - HOW SURE are you that you can do physical activity in each situation? - 5-point Likert Scale (1 = *I’m sure I can’t*, 5 = *I’m sure I can*) | - Turn off the TV even when there is a program on you enjoy - Limit your online computer time (e.g., emailing, browsing) to 1 hour per day - Leave the room where the TV is on, even if others are watching it - Plan ahead of time what TV shows you will watch during the week - Instead of just sitting listening to music, listen while you are being active (e.g., walking or dancing) - Set limits on how long you plan to talk on the telephone or text message with friends - Limit TV, video and computer games to only 2 hours per day |
| Sedentary reduction pros | - Please circle the answer that best applies to you when deciding whether or not to do sedentary activities. - 5-point Likert Scale (1 = *Strongly disagree*, 4 = *Strongly agree*) | - I think TV and computer/video games are boring. - Watching TV takes time away from doing other, more important things. - I would feel lazy and sluggish if I sat and watched TV for many hours. - I see too many commercials when I watch a lot of TV. - My parents would be pleased if I spent less time playing computer/video games. - Playing computer/video games sometimes hurts my eyes and gives me a headache. |
| Sedentary reduction cons | - Please circle the answer that best applies to you when deciding whether or not to do sedentary activities. - 5-point Likert Scale (1 = *Strongly disagree*, 4 = *Strongly agree*) | - I enjoy playing computer/video games for many hours at a time. - Watching TV or playing computer/video games is my way to escape from the world. - I feel good about myself when I do well at my favorite computer/video games. - Watching TV is one of my favorite forms of entertainment. - I find sitting and watching TV very relaxing. - My friends would be disappointed if I tried to spend less time chatting with them (e.g., talking on the phone, emailing, texting). |
| Sedentary enjoyment | - No stem - 5-point Likert Scale (1 = *Strongly disagree*, 5 = *Strongly agree*) | - I enjoy doing sedentary activities like watching TV or playing computer/video games. |
| Sedentary social support | - During a typical week, how often do you sit and watch TV or play electronic games (do not include time in exercise games like Wii or Dance Dance Revolution) with… - 5-point Likert Scale (0 = *Never*, 4 = *Everyday*) | - How often do you sit and watch TV or play electronic games with brothers/sisters? - How often do you sit and watch TV or play electronic games with a parent/guardian/caregiver? - How often do you sit and watch TV or play electronic games with friends? |
| Non-school sedentary psychosocial index | Index of the scales above | |
| Personal electronics | - Do you have the following items for your own use? - Dichotomous yes/no scale (0 = *No*, 1 = *Yes*) | - Cell phone - Hand-help video game - Personal stereo (iPod) - Social media account |
| Screens in bedroom | - Please indicate whether the following is in your bedroom. - Dichotomous yes/no scale (0 = *No*, 1 = *Yes*) | - TV - DVD - Music - Computer - Video games - Internet |
| Sedentary time rules | - Does your parent or guardian have the following rules, whether they remind you often or not? - Dichotomous yes/no scale (0 = *No*, 1 = *Yes*) | - No TV/DVD/computer before homework - Less than 2 hours TV/DVD/computer per day - No internet use without permission |
| Non-school sedentary environment index | Index of the scales above | |
| Physical activity self-efficacy | - HOW SURE are you that you can do physical activity in each situation? - 5-point Likert Scale (1 = *I’m sure I can’t*, 5 = *I’m sure I can*) | - Set aside time for physical activity on most days of the week - Get up early, even on weekends, to do physical activity - Do physical activity even when you have a lot of homework |
| Physical activity social support | - During a typical week: - 5-point Likert Scale (0 = *Never*, 4 = *Very Often*) | - How often does an adult in your household provide transportation to a place where you can do physical activity or play sports? - How often does an adult in your household do physical activity or play sports with you? - How often do your brothers/sisters or friends ask you to walk or bike to school or to a friend’s house? |
| Non-school physical activity psychosocial index | Index of the scales above | |
| Home physical activity equipment | - How often do you use these items in or around your home (or in a common apartment area)? - 5-point Likert Scale (0 = *Not available (don’t have)*, 4 = *Once a week or more*) | - Bike - Basketball hoop - Jump rope - Active video games - Sports equipment/balls - Swimming pool - Rollerblades/skateboard - Home aerobic equipment - Weight-lifting equipment - Water or snow equipment |
| Home physical activity environment index | Index of the scales above | |
