# Supplementary material for: Location-specific psychosocial and environmental correlates of physical activity and sedentary time in young adolescents: preliminary evidence for location-specific approaches from a cross-sectional observational study
Source: Int J Behav Nutr Phys Act. 2022 Aug 26;19:108. doi: 10.1186/s12966-022-01336-7 (PMC9419353; doi:10.1186/s12966-022-01336-7)
Supplement: Supplementary file 3 — Additional file 3: Supplementary Table 2. Descriptive statistics for psychosocial and environmental subscales comprising the indices. Includes table as supplemental information. [file 12966_2022_1336_MOESM3_ESM.docx]

| Supplementary Table 2.    *Descriptive statistics for psychosocial and environmental subscales comprising the indices* | | | |
| --- | --- | --- | --- |
|  | Mean | SD | Possible Range |
| **General variables** |  |  |  |
| Scales comprising general physical activity psychosocial index |  |  |  |
| Physical activity self-efficacy | 11.05 | (2.92) | 3-15 |
| Physical activity enjoyment | 4.32 | (0.97) | 1-5 |
| Physical activity pros | 17.92 | (2.32) | 5-25 |
| Physical activity cons*^┼^* | 13.72 | (2.14) | 5-20 |
| Physical activity social support | 4.70 | (2.05) | 0-8 |
| **Location-specific school variables** |  |  |  |
| Scales comprising the school physical activity environment index |  |  |  |
| PE and recess time | 148.84 | (133.42) | 0-575* |
| After school environment | 5.69 | (1.93) | 0-8 |
| School physical activity equipment | 4.66 | (1.50) | 0-6 |
| **Location-specific non-school variables** |  |  |  |
| Scales comprising the non-school sedentary psychosocial index |  |  |  |
| Sedentary reduction self-efficacy | 26.89 | (5.64) | 7-35 |
| Sedentary reduction pros | 16.71 | (3.16) | 6-24 |
| Sedentary reduction cons*^┼^* | 14.88 | (3.64) | 6-24 |
| Sedentary enjoyment*^┼^* | 1.90 | (0.92) | 1-5 |
| Sedentary social support*^┼^* | 8.59 | (2.13) | 0-12 |
| Scales comprising the non-school sedentary environment index |  |  |  |
| Personal electronics *^┼^* | 1.03 | (0.94) | 0-4 |
| Screens in bedroom *^┼^* | 3.48 | (1.71) | 0-6 |
| Sedentary time rules | 1.09 | (0.98) | 0-3 |
| Scales comprising the non-school physical activity psychosocial index |  |  |  |
| Physical activity self-efficacy | 10.16 | (3.19) | 3-15 |
| Physical activity social support | 5.80 | (2.76) | 0-12 |
| Scales comprising the home physical activity environment index |  |  |  |
| Home physical activity equipment | 14.72 | (6.87) | 0-40 |
| ^┼^=Variable was reverse coded when calculating index score;  *=observed maximum | | | |
