# Supplementary material for: Location-specific psychosocial and environmental correlates of physical activity and sedentary time in young adolescents: preliminary evidence for location-specific approaches from a cross-sectional observational study
Source: Int J Behav Nutr Phys Act. 2022 Aug 26;19:108. doi: 10.1186/s12966-022-01336-7 (PMC9419353; doi:10.1186/s12966-022-01336-7)
Supplement: Supplementary file 4 — Additional file 4: Supplementary Table 3. Partial correlations between physical activity and sedentary time within locations. Includes partial correlations for outcome variables as supplemental information. [file 12966_2022_1336_MOESM4_ESM.docx]

| Supplementary Table 3.  *Partial Correlations between Physical Activity and Sedentary Time Within Locations* | | | | |
| --- | --- | --- | --- | --- |
|  | School MVPA | Home MVPA | Other MVPA | Overall MVPA |
| School Sedentary Time | -.608** | NA | NA | NA |
| Home Sedentary Time | NA | -.554** | NA | NA |
| Other Sedentary Time | NA | NA | -.570** | NA |
| Overall Sedentary Time | NA | NA | NA | -.604** |
| **Correlation is significant at the p <0.01 level (2-tailed); NA = Correlation not tested; Each partial correlation adjusted for accelerometer wear time in that location | | | | |
