# Supplementary material for: Location-specific psychosocial and environmental correlates of physical activity and sedentary time in young adolescents: preliminary evidence for location-specific approaches from a cross-sectional observational study
Source: Int J Behav Nutr Phys Act. 2022 Aug 26;19:108. doi: 10.1186/s12966-022-01336-7 (PMC9419353; doi:10.1186/s12966-022-01336-7)
Supplement: Supplementary file 5 — Additional file 5. STROBE Statement—checklist of items that should be included in reports of observational studies. STROBE Checklist for the study. [file 12966_2022_1336_MOESM5_ESM.docx]

STROBE Statement—checklist of items that should be included in reports of observational studies

|  | Item No. | Recommendation | Page  No. | Relevant text from manuscript |
| --- | --- | --- | --- | --- |
| **Title and abstract** | 1 | (*a*) Indicate the study’s design with a commonly used term in the title or the abstract | 1,2 | See title (e.g., “correlates”) and abstract (e.g., “cross sectional) |
|  |  | (*b*) Provide in the abstract an informative and balanced summary of what was done and what was found | 2 | See abstract |
| Introduction | | | |  |
| Background/rationale | 2 | Explain the scientific background and rationale for the investigation being reported | See all of introduction section, 3-5 | “It is important to understand whether location-general and location-specific factors differ in their associations with location-specific activity. Few studies have examined location-general and location-specific influences on physical activity and sedentary time within different locations. Therefore, more research of the associations of location-general and location-specific psychosocial attributes and environmental features in relation to adolescents’ activity could inform more targeted and tailored interventions for sustaining physical activity across locations that play a large role in adolescents’ lives, such as their homes and schools.” |
| Objectives | 3 | State specific objectives, including any prespecified hypotheses | 5 | “The purpose of the present study was to investigate the associations of both location-general (i.e., across locations or not specific to any location) and location-specific psychosocial and environmental variables with adolescents’ physical activity and sedentary time at home, school, and all “other” locations. It was hypothesized…” |
| Methods | | | |  |
| Study design | 4 | Present key elements of study design early in the paper | 6 | “Present analyses involved data from the Teen Environment and Neighborhood (TEAN) Study…” |
| Setting | 5 | Describe the setting, locations, and relevant dates, including periods of recruitment, exposure, follow-up, and data collection | 6 | “Participants 12-16 years of age and one of their parents were recruited from 447 census block groups spanning the Baltimore, MD-Washington, DC and Seattle-King County, WA metropolitan areas from 2009-2011. Recruitment was balanced by season and evenly stratified across four quadrants representing combinations of neighborhoods (defined as census block groups) that were high or low neighborhood walkability and high or low median household income. Data collection took place during the school year only.” |
| Participants | 6 | (*a*) *Cohort study*—Give the eligibility criteria, and the sources and methods of selection of participants. Describe methods of follow-up  *Case-control study*—Give the eligibility criteria, and the sources and methods of case ascertainment and control selection. Give the rationale for the choice of cases and controls  *Cross-sectional study*—Give the eligibility criteria, and the sources and methods of selection of participants | 6 | “Potential participants were identified through a purchased list from a marketing company and were contacted by phone to gauge their interest in the study and complete eligibility screening. Adolescents were excluded from the study if they had any physical, medical, or cognitive limitations that would affect their physical activity or impact their ability to complete measures. Eligible and interested adolescents were instructed to wear an accelerometer and GPS tracker for seven days during waking hours.” |
|  |  | (*b*) *Cohort study*—For matched studies, give matching criteria and number of exposed and unexposed  *Case-control study*—For matched studies, give matching criteria and the number of controls per case | NA | NA |
| Variables | 7 | Clearly define all outcomes, exposures, predictors, potential confounders, and effect modifiers. Give diagnostic criteria, if applicable | 6-10 | See “measures” subsection. |
| Data sources/ measurement | 8* | For each variable of interest, give sources of data and details of methods of assessment (measurement). Describe comparability of assessment methods if there is more than one group | 6-10 | See “measures” subsection. |
| Bias | 9 | Describe any efforts to address potential sources of bias | 6-10 | Recruitment was balanced by season and evenly stratified across four quadrants representing combinations of neighborhoods; If participants did not spend ≥30 minutes/day in a location on average across days, the activity variables for those locations were set to missing. This location-specific time requirement aimed to increase the likelihood the data were representative of the adolescent’s typical activity in the location; Adolescents who did not wear both devices for ≥1 valid school day and ≥1 valid weekend day (n = 204) were also excluded to improve the likelihood that data were representative of a typical week of activity. |
| Study size | 10 | Explain how the study size was arrived at | 6 | Present analyses excluded adolescents who did not receive a GPS tracker or record any GPS data (n = 130), whose home address was not available in the geocoding database (n = 29; e.g., P.O. Box or otherwise failed to geocode), or who did not provide their school’s name/address or were homeschooled (n = 93). Adolescents who did not wear both devices for ≥1 valid school day and ≥1 valid weekend day (n = 204) were also excluded to improve the likelihood that data were representative of a typical week of activity. |

Continued on next page

| Quantitative variables | 11 | Explain how quantitative variables were handled in the analyses. If applicable, describe which groupings were chosen and why | 6-10 | Measures section describes quantitative variables for MVPA, sedentary variables, height and weight, location, as well as psychosocial and environmental variables |
| --- | --- | --- | --- | --- |
| Statistical methods | 12 | (*a*) Describe all statistical methods, including those used to control for confounding | 10-11 | See applicable information in the “Data Analytic Plan” section |
|  |  | (*b*) Describe any methods used to examine subgroups and interactions | 11 | “For interactions, we probed those with *P*-values ≤ 0.10. We plotted values at ±1 SD of each independent variable comprising the interaction to determine the relation of “high” (+1 SD) and “low” (-1 SD) values on each interacting indices with the activity variable. Original metrics (e.g., minutes/day) of the activity variables were used when probing interactions. We centered the Y axis of these plots at the mean value for the dependent variable and adjusted the axis bounds to reflect +1 SD and -1 SD below the mean.” |
|  |  | (*c*) Explain how missing data were addressed | 6-11 | See applicable information in “Methods” section |
|  |  | (*d*) *Cohort study*—If applicable, explain how loss to follow-up was addressed  *Case-control study*—If applicable, explain how matching of cases and controls was addressed  *Cross-sectional study*—If applicable, describe analytical methods taking account of sampling strategy | 11 | “All models were adjusted for study design variables (neighborhood walkability and income categories); the adolescent’s age, sex, and race/ethnicity; parent education; ActiGraph model; number of school and non-school days of device wear; and average minutes/day of wear time in the respective location.” |
|  |  | (*e*) Describe any sensitivity analyses | NA | NA |
| Results | | | | |
| Participants | 13* | (a) Report numbers of individuals at each stage of study—eg numbers potentially eligible, examined for eligibility, confirmed eligible, included in the study, completing follow-up, and analysed | 12 | See Table 1. |
|  |  | (b) Give reasons for non-participation at each stage | NA | NA |
|  |  | (c) Consider use of a flow diagram | NA | NA |
| Descriptive data | 14* | (a) Give characteristics of study participants (eg demographic, clinical, social) and information on exposures and potential confounders | 11-12 | See Table 1 and “Sociodemographic and Subscale Model Results” section |
|  |  | (b) Indicate number of participants with missing data for each variable of interest | 6 | See “participants and procedures” section |
|  |  | (c) *Cohort study*—Summarise follow-up time (eg, average and total amount) | NA | NA |
| Outcome data | 15* | *Cohort study*—Report numbers of outcome events or summary measures over time | NA | NA |
|  |  | *Case-control study—*Report numbers in each exposure category, or summary measures of exposure | NA | NA |
|  |  | *Cross-sectional study—*Report numbers of outcome events or summary measures | 12-14 | See “Results” section |
| Main results | 16 | (*a*) Give unadjusted estimates and, if applicable, confounder-adjusted estimates and their precision (eg, 95% confidence interval). Make clear which confounders were adjusted for and why they were included | 12-14 | See “Results” section and Tables 2-3 as well as Tables in the additional Files. |
|  |  | (*b*) Report category boundaries when continuous variables were categorized | NA | NA |
|  |  | (*c*) If relevant, consider translating estimates of relative risk into absolute risk for a meaningful time period | NA | NA |

Continued on next page

| Other analyses | 17 | Report other analyses done—eg analyses of subgroups and interactions, and sensitivity analyses | NA | NA |
| --- | --- | --- | --- | --- |
| Discussion | | | | |
| Key results | 18 | Summarise key results with reference to study objectives | 15 | See first paragraph of “Discussion” Section |
| Limitations | 19 | Discuss limitations of the study, taking into account sources of potential bias or imprecision. Discuss both direction and magnitude of any potential bias | 20-22 | See “Limitations” section. |
| Interpretation | 20 | Give a cautious overall interpretation of results considering objectives, limitations, multiplicity of analyses, results from similar studies, and other relevant evidence | 22 | See “Conclusions” section |
| Generalisability | 21 | Discuss the generalisability (external validity) of the study results | 20-22 | See “Limitations” section. |
| Other information | |  | | |
| Funding | 22 | Give the source of funding and the role of the funders for the present study and, if applicable, for the original study on which the present article is based | 23 | See “Funding” section in “Declarations” |

*Give information separately for cases and controls in case-control studies and, if applicable, for exposed and unexposed groups in cohort and cross-sectional studies.

**Note:** An Explanation and Elaboration article discusses each checklist item and gives methodological background and published examples of transparent reporting. The STROBE checklist is best used in conjunction with this article (freely available on the Web sites of PLoS Medicine at http://www.plosmedicine.org/, Annals of Internal Medicine at http://www.annals.org/, and Epidemiology at http://www.epidem.com/). Information on the STROBE Initiative is available at www.strobe-statement.org.
